# Global Burden of Colistin-Resistant Bacteria: Mobilized Colistin Resistance Genes Study (1980–2018)

**DOI:** 10.3390/microorganisms7100461

**Published:** 2019-10-16

**Authors:** Mohammed Elbediwi, Yan Li, Narayan Paudyal, Hang Pan, Xiaoliang Li, Shaohua Xie, Andreja Rajkovic, Youjun Feng, Weihuan Fang, Shelley C. Rankin, Min Yue

**Affiliations:** 1 Institute of Preventive Veterinary Sciences, Zhejiang University College of Animal Sciences, Hangzhou 310058, China; m.elbediwi@zju.edu.cn (M.E.); yanli3@zju.edu.cn (Y.L.); narayan.paudyal@outlook.com (N.P.); 11817015@zju.edu.cn (H.P.); xlli@zju.edu.cn (X.L.); whfang@zju.edu.cn (W.F.); 2 Nepal Agricultural Research Council (NARC), Animal Health Research Division (AHRD), Kathmandu 5459, Nepal; 3 Zhejiang Provincial Key Laboratory of Preventive Veterinary Medicine, Hangzhou 310058, China; 4 Upper Gastrointestinal Surgery, Department of Molecular Medicine and Surgery, Karolinska Institute, Karolinska University Hospital, 17176 Stockholm, Sweden; shaohua.xie@ki.se; 5 Department of Food technology, Food safety and Health, Faculty of Bioscience Engineering, Ghent University, 9000 Ghent, Belgium; andreja.rajkovic@ugent.be; 6 Department of Medical Microbiology & Parasitology, Zhejiang University School of Medicine, Hangzhou 310058, China; fengyj@zju.edu.cn; 7 Department of Pathobiology, University of Pennsylvania School of Veterinary Medicine, Philadelphia, PA 19104, USA; srankin@vet.upenn.edu

**Keywords:** mobilized colistin resistance (*mcr*) genes, *Escherichia coli*, reservoir, food-chain, antibiotic resistance, meta-analysis

## Abstract

Colistin is considered to be an antimicrobial of last-resort for the treatment of multidrug-resistant Gram-negative bacterial infections. The recent global dissemination of mobilized colistin resistance (*mcr*) genes is an urgent public health threat. An accurate estimate of the global prevalence of *mcr* genes, their reservoirs and the potential pathways for human transmission are required to implement control and prevention strategies, yet such data are lacking. Publications from four English (PubMed, Scopus, the Cochrane Database of Systematic Reviews and Web of Science) and two Chinese (CNKI and WANFANG) databases published between 18 November 2015 and 30 December 2018 were identified. In this systematic review and meta-analysis, the prevalence of *mcr* genes in bacteria isolated from humans, animals, the environment and food products were investigated. A total of 974 publications were identified. 202 observational studies were included in the systematic review and 71 in the meta-analysis. *mcr* genes were reported from 47 countries across six continents and the overall average prevalence was 4.7% (0.1–9.3%). China reported the highest number of *mcr*-positive strains. Pathogenic *Escherichia coli* (54%), isolated from animals (52%) and harboring an IncI2 plasmid (34%) were the bacteria with highest prevalence of *mcr* genes. The estimated prevalence of *mcr-1* pathogenic *E. coli* was higher in food-animals than in humans and food products, which suggests a role for foodborne transmission. This study provides a comprehensive assessment of prevalence of the *mcr* gene by source, organism, genotype and type of plasmid.

## 1. Introduction

The emergence of antimicrobial-resistant (AMR) bacteria has become one of the major threats to global public health, food safety and social-economic development [1,2,3]. Global estimates of the prevalence of bacteria that are resistant to critically important antimicrobials are required to implement control and prevention strategies, yet such data are lacking.

Carbapenem-resistant bacteria have severely challenged antimicrobial therapy. Colistin (polymyxin E) is a polycationic peptide antimicrobial that was isolated and characterized in 1949 [4]. It has been reintroduced as a “last-line” therapeutic drug, for the treatment of infections in hospitalized patients caused by carbapenem-resistant Gram-negative bacteria [5]. Before 2015, colistin resistance was perceived to be due to regulatory changes mediated by chromosomal genes (*phoPQ*, *pmrAB*, and *mgrB*) [6]. The discovery of plasmid-mediated mobilized colistin resistance (*mcr*) genes has had an obvious influence on established antibiotic therapy regimens [7]. Lack of effective antibiotics will inevitably compromise modern clinical medicine, especially the treatment of infectious diseases and surgery.

There have been an increasing number of reports on the identification of *mcr* genes in many bacterial species across the world since this gene was first characterized [8,9,10]. One retrospective study from China, showed the presence of the *mcr-1* gene in *E. coli* isolated from poultry in the 1980s and suggested that the emergence of the gene may be linked to the use of colistin as a growth promoter in the poultry industry [11]. Multiple *mcr* genes (*mcr-1* to *-8*) in addition to several *mcr-1* variants have been reported [12]. A recent study by Carroll et al. [13] described *mcr-9*, a novel *mcr* homologue in a multidrug-resistant (MDR) *Salmonella* Typhimurium strain isolated from a human patient in United States.

The *mcr* genes have been detected in a wide range of plasmid types such as IncI2, IncHI2, IncX4, IncP, IncF, and IncY. The bacteria that carry these genes have been isolated from pigs, poultry, and cattle and also from food products derived from these animals such as pork, chicken and beef, in addition to many environmental sources such as hospital sewage, rivers, and seas, [8]. Few studies have conducted an integrated analysis among animal hosts, bacterial species or genotypes, and plasmid types. Investigations on the role of the environment and/or a foodborne pathway for *mcr* dissemination are patchy and sometimes vague.

The co-occurrence of *mcr* and other resistance genes, especially those that confer resistance to carbapenem drugs [14], have already been reported. While this highlights the limitation of therapeutic choices, a recent study suggested that treatment with colistin in combination with other antibiotics is still effective against *mcr*-harboring bacteria in an animal infection model [15].

The objectives of this study were to determine (1) the global distribution of *mcr* genes; (2) potential reservoirs including the environment, animal hosts, humans, bacterial species, genotype, and plasmid type and (3) to perform an analysis on antibiotic resistance patterns in isolates obtained from the different reservoirs.

## 2. Methods

### 2.1. Search Strategy and Selection Criteria

**Figure 1:**
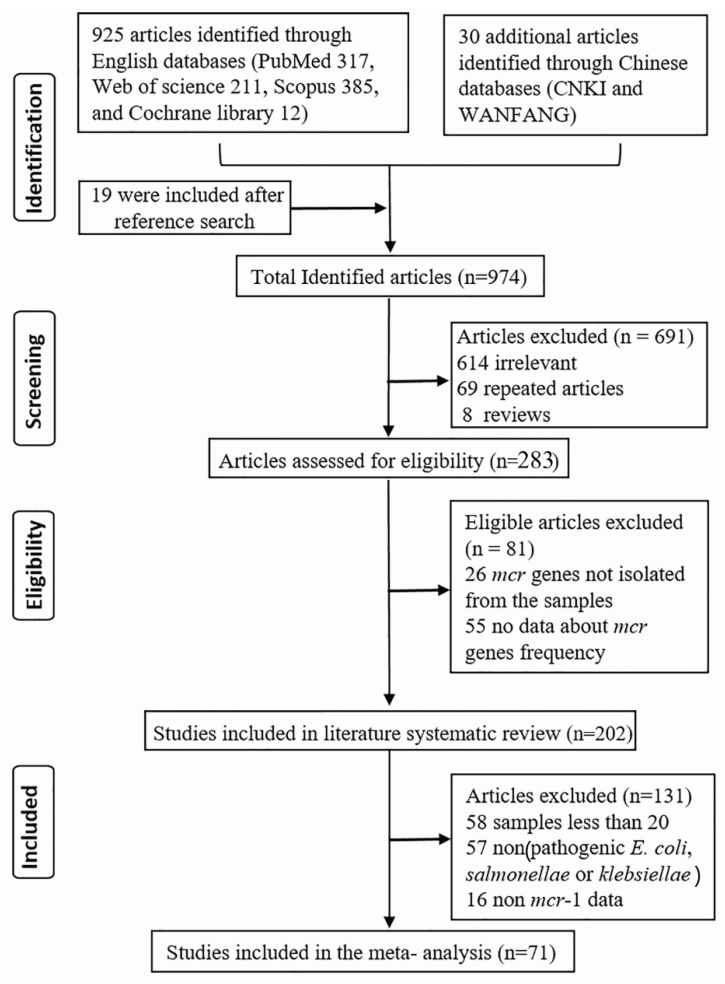
Flow diagram of identification and selection process included in systematic review and meta-analysis.

In compliance with PRISMA recommendations [16] (Figure 1), we conducted a systematic literature review and meta-analysis study. Four English (PubMed, Scopus, the Cochrane Database of Systematic Reviews, Web of Science) and two Chinese (CNKI and WANFANG) databases were searched individually for the published papers. We focused on those published between 18 November, 2015 (first publication on *mcr* gene discovery) and 30 December, 2018. We used some keywords to search relevant publications in both English and Chinese databases. The search key words, or strings, were “*mcr* genes and polymyxins”, “prevalence of *mcr* genes”, “occurrence of *mcr* genes”, “incidence of *mcr* genes”, “*mcr-1* OR *mcr-2* OR *mcr-3* OR *mcr-4* OR *mcr-5* OR *mcr-6* OR *mcr-7* OR *mcr-8* OR variant *mcr* genes” “colistin resistance”, and “plasmid-mediated colistin resistance”.

### 2.2. Study Selection

The systematic review was performed by four reviewers. Two reviewers (ME and NP) evaluated the data from English databases, and two reviewers (YL and HP) evaluated the data from Chinese databases. The search results were imported into the Endnote 7 references manager distributed by Clarivate Analytics and de-duplicated. Initial screening was done on the basis of title and abstract. Full text of the publications selected after initial screening was obtained from the library of Zhejiang University for data extraction. The relevant variables such as title, authors list, country of sampling, period of study, sampling reservoir, animal host, bacteria species and genotype, plasmid type, date of sampling and antimicrobial resistance pattern (if available) were extracted and arranged in a MS Excel spreadsheet specially formatted for the purpose.

### 2.3. Data Categorization

The scheme of data categorization summarized in Table 1 are shown with details in Supplemental Table S1 and Supplemental Table S2. The *mcr* variants among the various bacterial genera and species were also summarized (Table S3). The *E. coli* strains were split into two groups as pathogenic and non-pathogenic *E. coli*. Any known pathogenic serotype or variants such as O157 [17], or those that have been reported as the causative agent of human or animal infection [18] or as a pathogenic *E. coli* (in the selected publication) were categorized into pathogenic *E. coli* group (P. *E. coli*), whereas isolates which were reported from non-clinical cases and did not cause disease were categorized as a non-pathogenic *E. coli*.

### 2.4. Data Analysis

We calculated the frequency of identification of *mcr* genes and their relationship with different reservoirs (human, animal hosts, bacteria, plasmid types) and antibiotic resistance pattern for *mcr*-positive bacteria. Pivot table function of Microsoft Excel 2016 was used to calculate the descriptive analysis (as a percentage), and the prevalence of *mcr*-harboring strains among the total strains including 95% confidence intervals (CIs), the total number of resistant isolates (number of resistant isolates/total number of positive isolates from same species) to each individual antimicrobial drug. Additionally, the sequence type (ST) of pathogenic *E. coli* (P. *E. coli*) harboring *mcr-1* as the most frequent bacteria was also calculated. Student’s T-test and graphs were performed using GraphPad Prism 7. Among 202 articles that were included in the systematic review, only 134 articles mentioned the total number of strains and were included in a cumulative prevalence analysis. We excluded all the case studies, and any articles that did not mention the total number of strains.

The prevalence results are shown in percent with the 95% CI in parenthesis. The meta-analysis and Forest plotting were done using Open Meta-Analyst software. The data were analyzed in binary random model effects at 95% confidence interval. The values of heterogeneity (I^2^) [19] across the studies at 25%, 50%, and 75% were considered a slow, moderate, high heterogeneity, respectively.

## 3. Results

### 3.1. Data Selection

A total of 925 papers resulted from the initial search in English databases, 30 from Chinese databases and 19 additional English articles were included after reference search. After screening, based on the title and abstract, 691 (614 irrelevant, 69 repeated articles, and 8 reviews) were excluded. The remaining 283 publications were assessed for eligibility, and a further 80 (54 had no data about *mcr* genes and 26 publications where bacteria with *mcr* genes were not isolated from the samples) were excluded. We included 202 publications for synthesis of the systematic review. A meta-analysis of P. *E. coli*, *Salmonella*, and *K. pneumonia* harboring the *mcr-1* gene was carried out with 71 publications. One hundred and thirty-one were excluded (58 papers had less than 20 samples, 57 papers were on bacteria other than pathogenic *E. coli*, *Salmonella*, and *K. pneumoniae* and 2 papers were not related to *mcr-1* data). (Figure 1). We included 134 articles in the average prevalence analysis for the countries and the burdens. Ninety-eight of these 134 articles (73.3%) used a targeted PCR method and 36 (26.7%) used a selective culture media method (Table S4). We calculated the total number of isolated strains and the total numbers of *mcr* harboring strains in the selected articles. The prevalence (%) was calculated as (total number of isolated *mcr* harboring strains/the total number of isolates in each article).

### 3.2. Global Dissemination of mcr Genes

**Figure 2:**
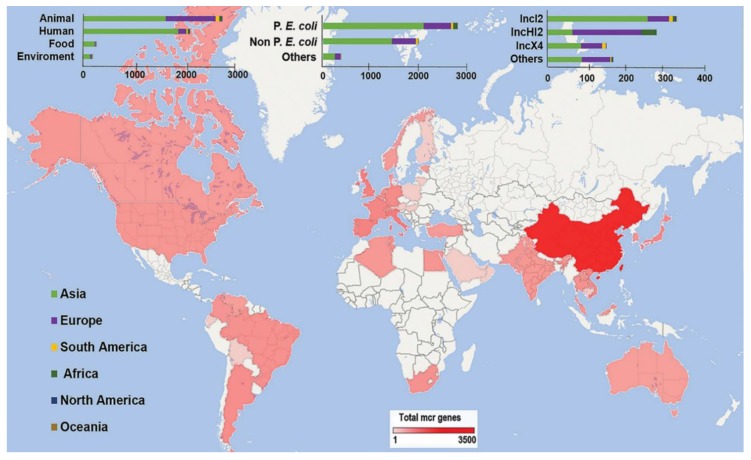
Global view of *mcr* genes along with the various hosts, bacteria and plasmid types. White colored countries refer to countries that have not yet reported studies on *mcr* genes.

The current global distribution of *mcr* genes, in relation to the various hosts, bacteria and plasmid types showed significant geographic segregation (Figure 2). A total of 5191 *mcr*-gene-harboring strains were reported in 202 publications. Of these, 4917 were of the *mcr-1* type while the remaining 274 were other variants. White colored countries in Figure 2 refer to countries that have not yet reported studies on *mcr* genes. P. *E. coli* (53%) among all bacteria, and animals (53%) among all hosts, were the most frequent *mcr* carriers reported globally. IncI2 (34%) plasmids were the most frequently reported plasmids.

### 3.3. Global Prevalence of mcr Genes by Sampling Source as a Reservoir

**Figure 3:**
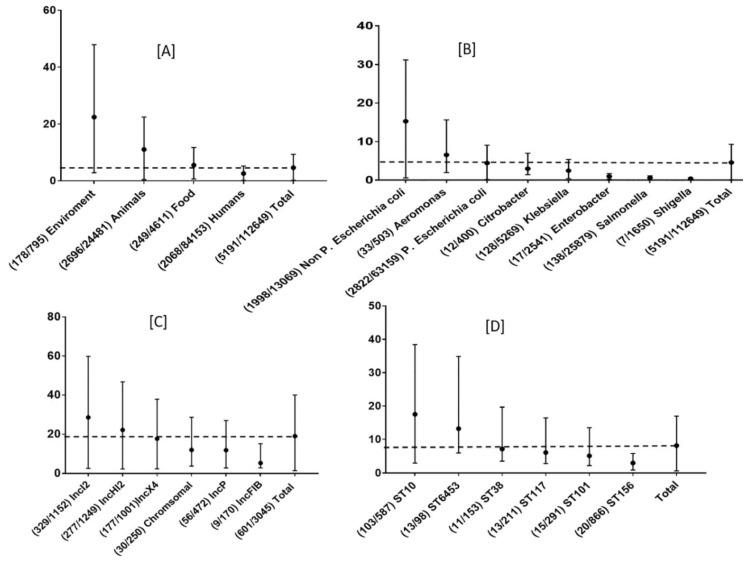
Prevalence of *mcr* genes from multiple sources (**A**), bacteria (**B**), plasmids (**C**) and pathogenic *E. coli* sequence type (ST) (**D**). ( ) contain the positive strains/total strains Values in (**A** and **B**) are the prevalence of positive strains in the total number of strains, in (**C**), the value is the prevalence of plasmid types and in (**D**) P. *E. coli* harboring *mcr*-1 shows the prevalence of different sequence types (ST) of the positive isolates.

The cumulative average prevalence of *mcr* genes was 4.6% (0.1–9.3%), with the highest in environment 22% (2.8–47.8%), followed by animals 11% (0.3–22.4%), food 5.4% (0.6–11.6%), and humans 2.5% (0.1–5.1%) (Figure 3A). China shares a similar pattern with the global picture and showed an overall prevalence in the environment of 39% (8.3–88.5%); animals 14% (0.7–30%); humans 4.5% (0.2–9.3%), and food products 4.9% (0.7–10.7%). Vietnam also has a very similar pattern to China, 14.7% from animals, 4.5% in humans and 4.9% in food products (Figure S1).

### 3.4. Global Prevalence of mcr Genes by Bacterial Genera and Species

There were 15 bacterial genera, mainly of the *Enterobacteriaceae* (11/15) (Supplemental Table S2, Supplemental Table S3), in which the presence of *mcr* has been reported to date. *E. coli* (non-pathogenic and pathogenic types) followed by *Salmonella*, *Klebsiella* and *Aeromonas* were the most common bacteria from which the *mcr* genes have been isolated. Non-pathogenic *E. coli* showed the highest *mcr* prevalence of 15.2%. The isolation rate of *E. coli* varies dramatically; China leads in terms of the numbers of reports made. The *mcr-1* gene was identified in 4917 strains, 274 strains had a variant *mcr* genes.

### 3.5. Types of mcr Gene and Their Associated Reservoir

**Figure 4:**
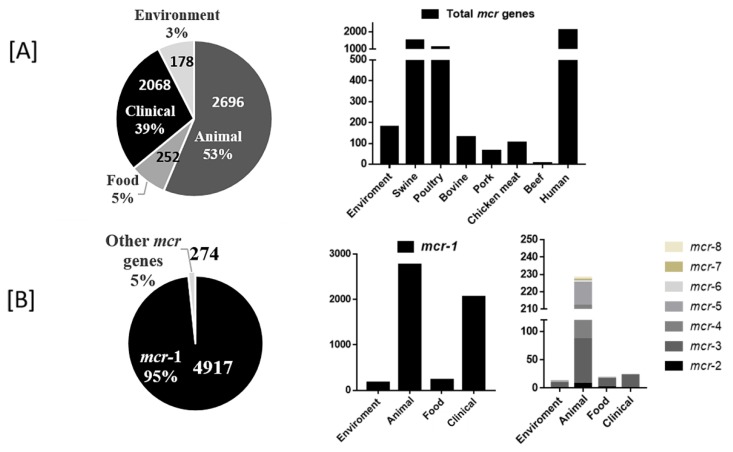
Frequency and distribution of *mcr* genes according to hosts and types. Hosts harboring *mcr* genes (**A**), *mcr* types (**B**).

The environmental samples had the highest prevalence of strains that carried the *mcr*-gene, 22.3% (2.8–47.8), although they had the lowest positive detections (3%, *n* = 178), likely due to lack of studies that looked at environmental sources. 2067 (39%) isolates were from human sources and the lowest prevalence 2.5% (0.1–5.1) was observed in these samples (Figure 3A and Figure 4A). Of the isolates, 62% were from clinical patients with an infectious disease and 38% were from asymptomatic carriers. Seventy-seven percent of the isolates were from feces. Isolates from pigs (among the animals) and chicken meat (among the foods) had the highest rate of *mcr* gene detection (Figure 4A). Live animals (swine, poultry, and bovine) had a higher frequency of *mcr* genes than isolates from meat samples (pork, chicken meat and beef). Concerning the entire host community, P. *E. coli* with *mcr-1* was the most common pathogen-*mcr* association (Figure 3A, Figure 4A). IncI2 was the most prevalent plasmid type at 28.5% (2.5–95.8) as well as the most frequent type associated with poultry, human, environmental and food isolates. The IncHI2 plasmid type was associated with isolates from animals (Figure 3C, Supplemental Figure S2B). There was no significant difference between the hosts in total number of isolates and the total number of plasmids (*p* > 0.05, Student T-test).

As compared to 4917 (95%) isolates that carried *mcr-1*, only 274 (5%) carried one of the other variant genes (*mcr-2* to *mcr-8*). Supplemental Figure S3 showed that *mcr-3* had a wide distribution in water, animal, food, and human isolates. P. *E. coli* had the highest frequency of *mcr* genes, except for *mcr-5* which was mainly isolated from *Salmonella* species. In addition, *mcr-7* and *mcr-8* were only reported in *K. pneumoniae* isolated from animals. IncI2 was the most common plasmid type in all hosts except animals. IncHI2, IncX4, CoIE and IncI2 plasmid types, had the highest frequency in *mcr-2*, *3*, *5* and *7* respectively.

### 3.6. Antimicrobial Resistance in mcr-Positive Bacteria

The results from antimicrobial susceptibility tests were different in the various studies because of the different antimicrobial agents tested, so we included only the resistance profiles of *mcr*-harboring bacteria in our analysis. Eighty-four percent (170/202) of the articles documented the antimicrobial resistance assays, and a high occurrence of co-resistance was observed (Table 2). All water and food isolates were susceptible to the two carbapenem drugs (imipenem and meropenem). P. *E. coli* isolated from poultry and bovine were susceptible to tigecycline, unlike those from swine, which were resistant to tigecycline. Human P. *E. coli* were susceptible to tigecycline and showed low resistance (2.76%) to the carbapenem drugs. *Klebsiella pneumoniae* strains from humans were resistant to carbapenem drugs (53.85%) and none of the isolates from animals were resistant. All of the *Salmonella* isolates were susceptible to carbapenems.

### 3.7. Role of mcr-1 Pathogenic E. coli, Salmonella, and Klebsiella in mcr Gene Dynamics

**Figure 5:**
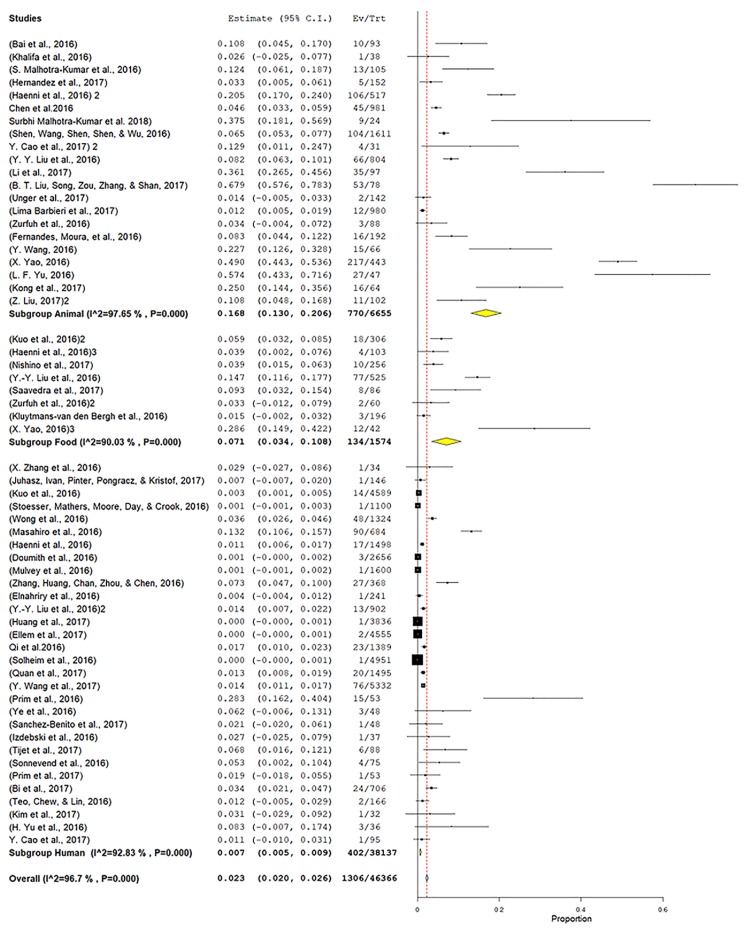
Prevalence of pathogenic *E. coli* carrying *mcr-1* in animals, foods and humans. *X*-axis is the proportion of the bacteria reported in individual studies as listed along the *Y*-axis, with the range of proportion in 95% confidence interval. Studies given higher weights are indicated by larger markers. The parallelograms in yellow and the square markers in black represent the pooled point estimate for the sub-group category and individual study, respectively. The horizontal lines of the parallelograms and the square markers represent the 95% confidence interval of this combined point estimate.

**Figure 6:**
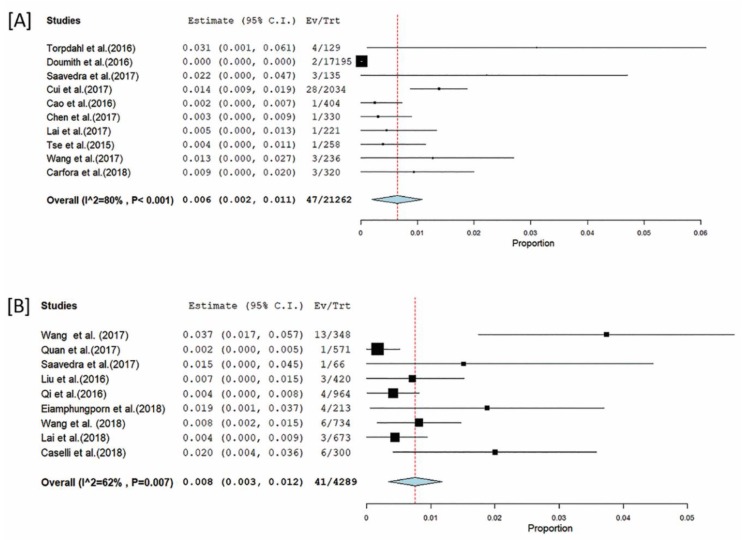
Prevalence of *Salmonella* (**A**) and *Klebsiella* (**B**) carrying *mcr-1*. *X*-axis is the proportion of the bacteria reported in individual studies as listed along the *Y*-axis, with the range of proportion in 95% confidence interval. Studies given higher weights are indicated by larger markers. The parallelograms in yellow and the square markers in black represent the pooled point estimate for the sub-group category and individual study, respectively. The horizontal lines of the parallelograms and the square markers represent the 95% confidence interval of this combined point estimate.

Because of their public health significance, we focused on pathogenic *E. coli*, *Salmonella,* and *Klebsiella*, in the meta-analysis studies. The average prevalence of *mcr-1*-harboring bacteria was calculated by a meta-analysis of 71 publications (Figure 5 and Figure 6). The analysis showed more heterogeneity in pathogenic *E. coli* (I^2^ = 97.65%) with an average prevalence of 23% (20–26, *p* < 0.001) (Figure 5) than *Salmonella* (I^2^ = 80%) with an average prevalence of 6% (2–11, *p* < 0.001) (Figure 6A) and *Klebsiella* (I^2^ = 62%) with an average prevalence of 8% (3–12, *p* < 0.001) (Figure 6B). Additionally, the average prevalence of pathogenic *E. coli* in humans was 0.7% (0.5–0.9, *p* < 0.001), in animals it was 16.8% (13–20.6, *p* < 0.001) and in foods it was 7.1% (3.4–10.8, *p* < 0.001). The highest heterogeneity was among the papers that dealt with animals’ isolates (I2 = 97.65%) and the lowest was in those isolates from food (I^2^ = 90.03%), and the humans (I^2^ = 92.83%) (Figure 5). The comparative analysis has not been made for humans, animals and foods of *Salmonella* and *Klebsiella* because of unavailability of their comparative data. Several studies included data on multiple hosts.

### 3.8. Common ST of P. E coli with mcr-1

**Figure 7:**
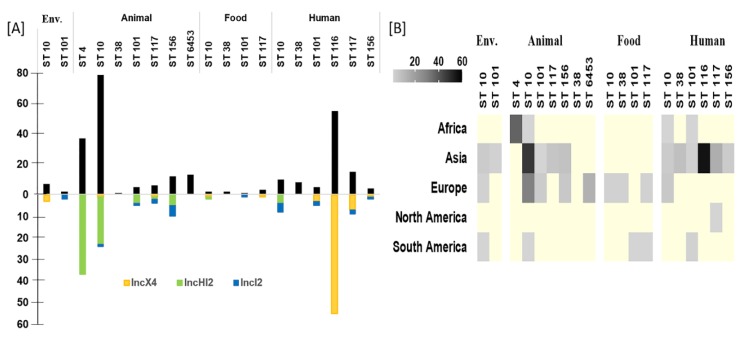
Role of P. *E. coli* STs in *mcr-1* gene dissemination. Particular P. *E. coli* STs that harbored *mcr-1* gene frequency (**A**). The global distribution of P. *E. coli* harboring *mcr-1* gene (**B**). Yellow cells in (**B**) refer to blank, Env. refers to environmental isolates. The scales refer to number of isolates.

The frequency of P. *E. coli* STs harboring *mcr-1* varied among different sources (Figure 7). ST10, the dominant ST in both animals and water, is the most common ST of those that carrying *mcr*-1 globally, also has the highest prevalence 17.5% (2.9–39.4) (Figure 3D). ST116 the most abundant ST in humans, has been reported only from China (Figure 7A).

## 4. Discussion

Since the first isolation of *mcr-1* in China in 2011 [7], *mcr*-harboring bacterial isolates have been reported from six continents (Asia, Europe, Africa, North America, South America, and Oceania) and over 27 bacterial species. The majority of studies have been conducted in China but *mcr*-harboring isolates were also reported from many European countries such as the UK, Spain, and Italy. The reason for the increasing reports may be due to long-term use of polymyxins in veterinary medicine in these countries. It should be noted that before 2005 there were no reports that identified the *mcr*-harboring isolates and most of the isolates currently reported to be *mcr* positive are historical isolates, dating back to as early as 1980. Similarly, global trade and travel either to countries with high or unknown prevalence (Canada [20], U.S. [21] and Japan [22]), importation of food from infected countries (Japan [23] and Tunisia [24]), and over-prescription of colistin in human medicine to treat highly resistant clinical pathogens (i.e., Argentina [25]) are also the suggested causes. To date, several other *mcr* gene variants have been identified, including *mcr-2, -3, -4, -5, -6, -7,* and *-8*, which share 81%, 32%, 34%, 36%, 83%, 35%, and 31% amino acid sequence identity, respectively, with *mcr-1* [26]. Recently *mcr-9* which is closely related to *mcr-3* was reported [13]. Minor variants have been reported for each of *mcr-2*, *-4,* and *-5*, whereas there are more than 10 *mcr-3* variants [26]. Among other *mcr* genes, the earliest *mcr-3* was discovered in 2005 in Germany [27]; whereas *mcr-2* (2009), *-4* (2013), *-5* (2011), *-6* (2015), and *-7* (2014) [26,27,28,29], were identified in strains collected over the past decade. This supports the hypothesis that *mcr* genes existed far earlier than first reported. Moreover, all *mcr* genes except (*mcr-6* and *mcr-9*) have been detected in samples from China. Thus far, *mcr-2* (Belgium and Spain), *mcr-3* (Brazil, Denmark, France, Germany, Japan, Spain and Thailand), *mcr-4* (Italy and Spain), *mcr-5* (Colombia, Japan, Spain and Germany), and *mcr-9* (US) [27,29,30,31,32,33,34,35,36,37].

A report by Biswas et al. [38] demonstrated that *mcr-3*-harboring *Salmonella* Typhimurium ST34 is highly likely to be linked with international travel. Two recent studies reported that *Moraxella* spp. a common pathogen associated with animals, but that may also be human pathogens, has been identified as a potential source of *mcr*-like genes [36,39]. A recent study described that the newly found *mcr-9* gene in *Salmonella* Typhimurium, from a clinical isolate in USA, was capable of conferring phenotypic resistance to colistin in *Enterobacteriaceae*, making it a relevant concern to public health [13]. Most of the studies where the *mcr* genes were reported included a screening approach for MDR strains (a pre-selected population of strains). Different screening methods to isolate *mcr* positive organisms were conducted in each study, the bibliography analyses may be biased by the size of the countries, missing data and their scientific intentions according to their priorities. These limitations might influence the global and individual country prevalence interpreted in current study.

### 4.1. Role of mcr-Positive Bacteria via Food-Chain Transmission

Colistin use in animal production is well-recognized as a driver for the emergence of *mcr*-positive bacteria [7]. Bacteria from animal gut and feces play an important role in persistence and transmission of bacteria that contain *mcr* genes to humans [40,41,42,43,44]. Bacteria with *mcr* genes have also been detected in food products [23], wastewater [45], rivers, seawater [46], and humans [47]. The estimated overall prevalence in the animal and food samples were higher than in the human samples (Figure 5, Figure 3A), and this supports the hypothesis of that the food-chain plays a role in *mcr* transmission. Additional large-scale investigations in China indicates that aquaculture could be another important reservoir for food-chain transmission of *mcr*-harboring bacteria to humans [48,49,50,51].

### 4.2. Role of Plasmids in mcr Dissemination

The *mcr* genes are generally hosted on bacterial plasmids that are highly mobile and this may accelerate the spread of resistance under selection pressure [52]. About 61% (123/202) of the articles have reported the plasmid type which our results were based on. The other articles that have not mentioned were case reports and surveys. The IncI2, IncX4 and IncHI2 are the most common plasmid types that carry *mcr* genes. While the plasmid IncI2 is common in Asia [53], Oceania [52], North and South America [54], IncHI2 is more frequently reported in Europe [47] and Africa [24] (Figure 2). The IncI2 plasmid usually carried the partitioning genes (*yafA*/*yafB*), type IV pilus and shufflon, acid resistance and biofilm production, which enhance the conjugation and adherence of the bacteria [55] with this plasmid to the epithelial cells. Moreover, IncHI2 plasmids are renowned by their ability to transfer by conjugation in a wide range of temperatures [56]. IncHI2 plasmids are known to carry several resistance genes, this information shows that even if a change of antimicrobial therapy occurs during treatment, there will still be a chance to co select for the *mcr-1* gene. Also, Several genes, i.e., *relE* and *relB* (antitoxin system), *mucAB* (mutagenesis induction system), *phi* (bacteriophage inhibition), and colicin, tellurite, and heavy metals together, likely play a key role in the stability of IncHI2 plasmids [57]. The *mcr-1* gene is associated with insertion sequence IS*Apl1* which belongs to IS*30* family [58]. A previous study of *mcr-1* sequences demonstrated that *mcr-1* was part of a ~2609-bp region flanked by one copy, two copies, or no copies of IS*Apl1*. Recently, Snesrud et al. [59] demonstrated that all *mcr-1* structures can be explained by loss of one, or both, copies of IS*Apl1* from an ancestral Tn*6330* [60]. Transferable plasmids that harbor *mcr* genes are detected in isolates of same clonal backgrounds. The plasmids belonging to the same incompatibility group are likely to be involved in the dissemination of *mcr* via the food-chain [61].

### 4.3. Role of P. E. coli Genotype for mcr Dissemination

Our results show that *E. coli* ST10 (the most frequent in many continents) and ST101 (widely distributed among multiple hosts) are the major clones that harbor *mcr-1* genes. Our analysis revealed that ST10 is the dominant ST in both animals and water as mentioned earlier [62]. ST10 and ST101 belong to phylogroups A and B1. These two groups produce enzymes such as ESBLs [63], ampC [64], carbapenemase, and mBLs [65] that degrade beta lactam antimicrobials. The presence of beta-lactamase resistance can accelerate the dissemination and persistence of these clones. ST116, with an IncX4 plasmid, is the most abundant clone in human patients, and has not yet been reported in any other hosts. The IncX4 plasmid in ST116 *E. coli* strains isolated from humans is likely the result of conjugation from ST10 strains thatoriginated from water or food [40]. A high rate of conjugation may be enhanced by the genes essential for synthesis and assembly of the pilus, conjugative function [66,67], and *mrk* fimbria [68,69,70]).

### 4.4. Rational Antibiotic Choice for mcr-Carrying Bacterial Infections

Isolates that carry *mcr* genes, and show resistance to colistin, were also frequently shown to possess other antibiotic-resistant determinants [47]. Interestingly, a few studies reported that *mcr*-harboring P. *E. coli* from human [53] and pig [71] are phenotypically susceptible to *colistin in vitro.* This might be attributed to suppression of *mcr* genes [72], or unknown reasons [73]. Some reports demonstrated the effectiveness of carbapenem drugs to treat infections caused by colistin-resistant isolates (Figure 3) [46,47]. The new combination regimen of a carbapenem and tigecycline [74] has also shown effectiveness against human infections. The resistance of most human clinical *K. pneumonia* isolates to multiple antimicrobials is a serious concern [75]. P. *E. coli* from animal samples did not show any resistance to tigecycline, except the isolates from pigs. Recently, some studies have reported that several antibiotics, in combination with colistin, display growth-inhibition at levels below their corresponding clinical breakpoints such as the combination of colistin and clarithromycin [15] and colistin and amikacin [76]. Further investigation on effective antibiotic therapies, including combination therapy, is needed to treat *mcr*-carrying bacterial infection.

## 5. Summary

We provide a comprehensive picture of *mcr* genes from animal hosts, bacterial species, bacterial genotype, and plasmid types in 47 countries across six continents. The significant role of the food-chain and/or the environment in *mcr* gene dissemination and their relationship with other suitable vectors (animal hosts, bacteria species, bacterial genotype, plasmid type) warrants further investigations. Co-resistance of *mcr*-positive pathogen with other antimicrobials that are critical for the treatment of drug resistant bacterial infections is an increasing concern, both in human or veterinary medicine.

## Figures and Tables

**Table 1:** Scheme of the data categorization.

Categories	Includes	Sub-Includes
Environmental	Sewage, river, and sea water samples	
Animal	Pig, poultry, cattle, and other animal samples	
	Poultry	Chicken, turkey, and muscovy duck
	Other animals	Migratory birds, penguins, reptiles, kelp gulls and dogs
Food	Pork, chicken meat, beef, and other foods samples	
	Other foods	Vegetables and foods from unknown sources
Human	Fecal and other samples	
	Other samples	Blood, urine, pus, sperm, abdominal and peritoneal fluids, sputum, and ascites
Harboring bacteria	Pathogenic *E. coli* (P. *E. coli*), *Salmonella* spp., *Klebsiella pneumoniae*, other bacteria	
	*Salmonella* spp.	*S.* Typhimurium, *S.* Paratyphi B, *S.* Virchow, *S.* Rissen, *S.* Indiana and *S.* Derby, *S.* London
	Other bacteria	*Citrobacter freundii*, non-pathogenic. *E. coli*, *Citrobacter braakii, Enterobacter cloacae, Shigella sonnei, Aeromonas veronii, Enterobacter aerogenes, Cronobacter sakazakii, Kluyvera ascorbata, Acinetobacter, Klebsiella variicola, Moraxella pluranimalium, Klebsiella oxytoca, Providencia alcalifaciens,* and *Raoultella ornithinolytica*
Plasmid types	IncI2, IncHI2, IncX4	
	Other plasmids	IncF, IncFIB, Inck, Incx, IncP, IncFII, and ColE

**Table 2:** Antibiotic resistance of isolates that harbor *mcr* genes.

Sources		CST	TGC	GEN	CIP	AMK	CTX	CFX	TET	TMP-SMX	FOS	MEM	IMP	AMP	KAN
Human	P. *E. coli*	98.69% (993)	3.39% (59)	59.55% (178)	44.09% (973)	22.60% (146)	79.61% (152)	83.72% (43)	96.43% (28)	83.08% (65)	48.62% (109)	2.76% (979)	2.76% (979)	93.33% (150)	95.00% (20)
Swine		99.37% (158)	100.00% (3)	86.67% (15)	90.91% (11)	6.06% (33)	47.06% (34)	100.00% (3)	100.00% 98)	98.98% (98)	100.00% (27)	43.64% (55)	40.38% (52)	43.75% (48)	80.65% (33)
Poultry		100.00% (125)	0.00% (38)	61.90% (21)	97.58% (124)	16.67% (18)	100.00% (55)	100.00% (3)	96.43% (56)	98.04% (51)	100.00% (54)	0.00% (3)	0.00% (3)	94.59% (74)	26.32% (19)
Bovine		100.00% (17)	0% (5)	80.00% (5)	100.00% (6)	NA	83.33% (6)	83.33% (6)	100.00% (6)	100.00% (5)	0.00% (5)	0.00% (1)	0.00% (1)	100.00% (6)	100.00% (1)
Food		97.83% (45)	100.00% (1)	33.33% (18)	69.57% (7)	100.00% (1)	81.25% (16)	100.00% (5)	84.21% (19)	87.50% (24)	100.00% (3)	0.00% (7)	0.00% (7)	81.25% (16)	40.00% (5)
Environmental		87.10% (31)	33.33% (24)	60.00% (5)	36.00% (25)	72.73% (11)	100.00% (1)	100.00% (2)	100.00% (3)	100.00% (1)	100.00% (1)	9.09% (30)	9.09% (33)	100.00% (3)	NA
Human	*K. pneumoniae*	100.00% (36)	21.40% (7)	91.67% (12)	63.64% (11)	33.33% (6)	91.67% (12)	100.00% (6)	100.00% (4)	60.00% (5)	66.67% (3)	53.85% (13)	53.85% (13)	90.00% (10)	33.3% (6)
Animals		100.00% (13)	NA	100.00% (4)	100.00% (4)	100.00% (1)	100.00% (4)	100.00% (4)	91.67% (11)	100.00% (9)	NA	0.00% (5)	0.00% (5)	100.00% (4)	0.00% (1)
Human	*Salmonella*	100.00% (32)	NA	43.59% (39)	24.14% (29)	NA	65.52% (29)	100.00% (1)	80.65% (31)	63.16% (19)	NA	0.00% (2)	0.00% (2)	90.00% (30)	90.00% (30)
Animals		100.00% (36)	NA	96.55% (29)	97.30% (36)	96.4% (26)	87.50% (8)	0.00% (36)	100.00% (10)	100.00% (34)	96.15% (26)	0.00% (2)	0.00% (2)	100.00% (54)	100.00% (3)
Human	Other isolates	60.00% (5)	100.00% (1)	100.00% (3)	75.00% (4)	66.67% (3)	100.00% (1)	0.00% (2)	100.00% (2)	NA	NA	14.2% (6)	75.00% (5)	100.00% (2)	NA
Animals		96.34% (273)	0.00% (152)	31.84% (223)	76.50% (234)	1.94% (155)	93.33% (165)	99.34% (152)	96.41% (232)	NA	100.00% (3)	0.86% (233)	48.07% (231)	100.00% (71)	100.00% (3)

( ) contain the number of isolates. Antibiotics: CST—Colistin, TGC—Tigecycline, GEN—Gentamicin, CIP—Ciprofloxacin, AMK—Amikacin, CTX—Cefotaxime, CFX—Cefoxitin, TET—Tetracycline, TMP-SMX—Trimethoprim-sulphamethoxazole, Fos—Fosfomycin, MEM—Meropenem, IMP—Imipenem, AMP—Ampicillin, KAN—Kanamycin.

## Supplementary Materials

The following are available online at https://www.mdpi.com/2076-2607/7/10/461/s1.

## Data Sharing

All of our results are available for further investigation in the appendix and online.
