# Supplementary material for: Global Burden of Colistin-Resistant Bacteria: Mobilized Colistin Resistance Genes Study (1980–2018)
Source: Microorganisms. 2019 Oct 16;7(10):461. doi: 10.3390/microorganisms7100461 (PMC6843232; doi:10.3390/microorganisms7100461)
Supplement: Supplementary file 1 [file microorganisms-07-00461-s001.pdf]

## Contents

**Tables and Figures....................................................................................................................1**

Supplemental table 1. Extracted data summary……………………………………….................2

Supplemental table 2. Data included in the systematic review ...................................................5

Supplemental table 3. Frequency of bacteria harboring different *mcr* variants .......................... 48

Supplemental table 4. Numbers of articles that mentioned the *mcr* carrying strains isolation methods ………………………………………………………………..........................49

Supplemental table 5. Country-level frequency of bacteria harboring *mcr* gene ....................... 50

Supplemental table 6. Estimated clinical *mcr* -carrying isolates .............................................. 52

Supplemental figure 1. Prevalence of *mcr* genes sources in China and Vietnam........................ 53

Supplemental figure 2. Harboring bacteria and carrying plasmids of *mcr* genes.........................54

Supplemental figure 3. Distribution of *mcr* by types, sources, harboring bacteria and carrying plasmids. ………………………………………………………….................................55

Supplemental references................................................................................................... 56

**Supplemental table 1. Extracted data summary**

| Host | | Bacteria | No. of papers | *mcr* types | Plasmid types | Countries |
|---|---|---|---|---|---|---|
| **Water** | Sewage rivers and seas | P. *E. coli* | 4 | *mcr*-1 | IncX4, IncHI2, IncI2, others | Brazil[1], China[2], Italy[3], Spain[4] |
| | | *Salmonella* spp. | 1 | *mcr*-5 | ColE | Germany[5] |
| | | *K. pneumoniae* | 3 | *mcr*-1 | IncHI2, IncI2, IncP | China[6,7], Spain[4] |
| | | Other bacteria | 15 | *mcr*-1 *mcr*-3 | IncHI2, IncI2 | Algeria[8], China[7,9-11], China[10,12-16], Norway[17], Malaysia[18], Switzerland[19] |
| **Animal** | Swine | P. *E. coli* | 26 | *mcr*-1 *mcr*-2 *mcr*-3 *mcr*-4 | IncX4, IncHI2, IncI2, others | Belgium[20-22], Brazil[23], China[13,22,24-35], Estonia[36], Germany[37], Laos[38], Portugal[39], South Korea[40]Spain[22,41,42], United Kingdom[43,44], U.S[45], Venezuela[46], Vietnam[47] |
| | | *Salmonella* spp. | 4 | *mcr*-1 *mcr*-4 | IncHI2, IncI2 | China[48], Italy[22], Spain[41], United Kingdom[43] |
| | | *K. pneumoniae* | 2 | *mcr*-1 *mcr-8* | IncHI2, IncI2, IncP | China[49] Portugal[39] |
| | | Other Bacteria | 12 | *mcr*-1 *mcr*-2 *mcr*-3 *mcr*-5 *mcr*-6 | IncI2 | China[50-53], England[54,55], Japan[56], France[57,58], Lebanon[59], Germany[60]Spain[58,61], Vietnam[47] |
| | Poultry | P. *E. coli* | 25 | *mcr*-1 *mcr*-3 | IncX4, IncHI2, IncI2, others | Algeria[38,62], Argentina[63], Brazil [23], China[12,14,31,64-70], Egypt[64,71], Italy[69,72], Pakistan[73], Portugal[74], Spain[41], Tunisia[75,76], Vietnam[77] |
| | | *Salmonella* spp. | 7 | *mcr*-1 *mcr*-5 | IncI2, IncP, ColE | China[78-82], Germany[5], Italy[83] |
| | | *K. pneumoniae* | 4 | *mcr*-1 *mcr-7* | | China[49,78,144,145] |
| | | | 12 | *mcr*-1 | IncI2 | |

| | | | | | | |
|---|---|---|---|---|---|---|
| | | Other Bacteria | | | | Brazil[84], China[85,86], France[57,58], Germany[87], Lebanon[88], Netherland[58], Japan[56], Pakistan[89] |
| | Cattle | P. *E. coli* | 5 | *mcr*-1<br>*mcr*-2<br>*mcr*-3 | IncHI2, IncX4, others | Belgium[20,21], Egypt[90], France[91], Spain[92] |
| | | Other Bacteria | 1 | *mcr*-1 | IncI2 | Japan[56] |
| | Other animals | P. *E. coli* | 10 | *mcr*-1 | IncHI2, IncX4, others | Argentina[87], Brazil[1], China [14,16], France[93], Germany[94], Italy[69,94], Lithuania[95], Vietnam[96] |
| | | Other Bacteria | 1 | *mcr*-1 | IncX | China[97] |
| **Food** | Pork | P. *E. coli* | 3 | *mcr*-1 | IncX4 | China[26,98], Japan[99] |
| | | *Salmonella* spp. | 1 | *mcr*-1<br>*mcr*-2 | IncX4 | Belgium[100] |
| | | Other Bacteria | 1 | *mcr*-1 | NA | China[50] |
| | Chicken meat | P. *E. coli* | 11 | *mcr*-1 | IncX4, incI2, others | China[12,14,26,98,101,102], Japan[99], Italy[83], Netherland[103], Switzerland[104], Turkey[105] |
| | | *Salmonella* spp. | 3 | *mcr*-1<br>*mcr*-5 | IncHI2, IncX4, others | China[82], England and wales[106], Germany[5], Italy[83] |
| | | Other Bacteria | 1 | *mcr*-3 | Chromosome, Others | China[107] |
| | Beef | P. *E. coli* | 2 | *mcr*-1 | other | Canada[108], China[98] |
| | Other foods | P. *E. coli* | 6 | *mcr*-1 | UN | China[.109,110], Colombia[111], Switzerland[19], Vietnam[112] |
| | | *Salmonella* spp. | 2 | *mcr*-1 | IncHI2 | China[113,114],Colombia[111], Portugal[115] |
| | | *K. pneumoniae* | 1 | *mcr*-1 | UN | Colombia[111] |
| | | Other Bacteria | 2 | *mcr*-1 | IncHI2 | Bolivia[116], China[110] |
| **Human** | Fecal | P. *E. coli* | 24 | *mcr*-1 | IncX4, IncHI2, IncI2 | Austria[117], Cambodia[118], China[98,119-126], Japan[127-130], Finland[131], Netherland[132,133], South Korea[134], Spain[135], U.S.[136,137], Switzerland[104,138,139], |

| | | | | | |
|---|---|---|---|---|---|
| | | *Salmonella* spp. | 5 | *mcr*-1 | IncX4, IncHI2, IncI2, others | China[142-48,81,140] |
| | | *K. pneumoniae* | 4 | *mcr*-1 | IncX4 | Italy[143], France[144], U.S[145], Japan[129] |
| | | Other Bacteria | 3 | *mcr*-1<br>*mcr-3* | IncX4<br>IncI2 | China[146,147] Vietnam [148] |
| | Other samples | P. *E. coli* | 53 | *mcr*-1<br>*mcr-3* | IncX4, IncI2, others | Algeria[149], Australia[150], Argentina and Canada[151], Belgium [152], Brazil[1,153], Bahrain, K.S.A and U.A.E[154], China[7,32,98,119,155-170], Ecuador[171], Egypt[172], Hungary[173], Italy[3,8,174-176], Malaysia[18], Norway [177],Oman[178], Poland[179], Portugal[180,181], Spain[182-184], Singapore[185], South Africa[186-188], South Korea[189], Thailand[190,191], U.S.[192-194], Vietnam[195] |
| | | *Salmonella* spp. | 3 | *mcr*-1 | IncX4, IncI2 | China[32], Denmark[196], U.S[145] |
| | | *K. pneumoniae* | 7 | *mcr*-1 | IncX4 | Brazil[197], China[32,156,161], Thailand[198], India[199], Portugal[200] |
| | | Other Bacteria | 5 | *mcr*-1<br>*mcr-3* | NA | China[11,168,201], Italy[166], Singapore[202], South Korea[189] |

- N.B: About 63 articles included data on more than one host.
- NA: Data are not available.

## Supplemental table 2. Data included in the systematic review

| N | DOI | The title of the reference | Year | Country | Continent | Host | Sample type | Species | No. of Strain | Total *mcr*-1 gene | Total sum of other *mcr* genes | Other *mcr* types | Total *mcr* genes | Plasmids Count | Plasmid Types | ST of *E.coli* carrying *mcr*-1 | Count of ST of E.coli carrying mcr-1 |
|---|---|---|---|---|---|---|---|---|---|---|---|---|---|---|---|---|---|
| **1** | 10.1128/AAC.01075-16 | *mcr -1.2, a New mcr Variant Carried on a Transferable Plasmid from a Colistin-Resistant KPC Carbapenemase-Producing Klebsiella pneumoniae Strain of Sequence Type 512* | 2014 | Italy | Europe | Human | Fecal | *Klebsiella pneumoniae* | 1 | 2 | | mcr1.2 | 2 | 1 | IncX4 | | |
| **2** | 10.3201/eid2305.161942 | *mcr*-1 Colistin Resistance in ESBL-Producing *Klebsiella pneumoniae*, France. | 2016 | France | Europe | Human | Fecal | *Klebsiella pneumoniae* | | 1 | | | 1 | 1 | IncX4 | | |
| **3** | 10.1128/AAC.01272-17 | Chromosome-Mediated *mcr*-3 Variants in *Aeromonas veronii* from Chicken Meat | 2015 | China | Asia | Poultry | Chicken meat | *Aeromonas veronii* | 16 | | 2 | mcr3.2 | 2 | 2 | Chromsomal | | |
| **4** | 10.1128/AAC.00234-17 | Colistin-Resistant *mcr*-1-Positive *Escherichia coli* on Public Beaches, an Infectious Threat Emerging in Recreational Waters. | 2016 | Brazil | South America | Water | Water | P. *Escherichia coli* Serotype (ONT:H55, O9:H4, O54:H32) | | 3 | | | 3 | 3 | IncX4 | 10 | 2 |
| | | Colistin-Resistant *mcr*-1-Positive *Escherichia coli* on Public Beaches, an Infectious Threat Emerging in Recreational Waters. | 2016 | Brazil | South America | Poultry | Migratory penguin | P. *Escherichia coli* Serotype (ONT:H32) | | 1 | | | 1 | 1 | IncX4 | 46 | 1 |
| | | Colistin-Resistant *mcr*-1-Positive *Escherichia coli* on Public Beaches, an Infectious Threat Emerging in Recreational Waters. | 2016 | Brazil | South America | Human | Blood , Urine, Abdomen | P. *Escherichia coli* Serotype (ONT:H9) | | 1 | | | 1 | 1 | IncX4 | 1638 | 1 |
| | | Colistin-Resistant *mcr*-1-Positive *Escherichia coli* on Public Beaches, an Infectious Threat Emerging in Recreational Waters. | | | | | | | | | | | | | | 46 | 1 |
| | | Colistin-Resistant *mcr*-1-Positive *Escherichia coli* on Public Beaches, an Infectious Threat Emerging in Recreational Waters. | | | | | | | | | | | | | | 101 | 1 |
| **5** | 10.3201/eid2308.170162 | *mcr*-1 and blaKPC-3 in *Escherichia coli* Sequence Type 744 after Meropenem and Colistin Therapy, Portugal | 2016 | Portugal | Europe | Human | Urine | P. *Escherichia coli* | | 1 | | | 1 | 1 | IncX4 | 744 | 1 |
| **6** | 10.1016/j.jgar.2017.01.007 | Green-based real-time PCR method for improved detection of *mcr*-1-mediated colistin resistance in human stool samples. | 2015 | Switzerland | Europe | Human | Fecal | P. *Escherichia coli* | | 3 | | | 3 | | | | |
| **7** | 10.1016/j.diagmicrobio.2017.07.005 | Case report of transient *mcr*-1-haboring Escherichia coli with concurrent Staphylococcus aureus bacteremia in Long Beach, California. | 2016 | United States | North America | Human | Blood and urine | P. *Escherichia coli* | | 1 | | | 1 | | | | |

| | | | | | | | | | | | | | | | | | |
|---|---|---|---|---|---|---|---|---|---|---|---|---|---|---|---|---|---|
| **8** | 10.1016/j.ijantimicag.2017.06.011 | Colistin resistance gene *mcr*-1 in gut flora of children. | 2016 | China | Asia | Human | Fecal | P. *Escherichia coli* | 14 | 16 | | | 16 | 16 | IncX4 | 117 | 1 |
| | | Colistin resistance gene *mcr*-1 in gut flora of children. | 2016 | China | Asia | Human | Fecal | *Citrobacter freundii* | | 1 | | | 1 | 1 | IncX4 | 101 | 1 |
| | | Colistin resistance gene *mcr*-1 in gut flora of children. | | | | | | | | | | | | | | 131 | 1 |
| | | Colistin resistance gene *mcr*-1 in gut flora of children. | | | | | | | | | | | | | | 1638 | 1 |
| | | Colistin resistance gene *mcr*-1 in gut flora of children. | | | | | | | | | | | | | | 2967 | 1 |
| | | Colistin resistance gene *mcr*-1 in gut flora of children. | | | | | | | | | | | | | | 1193 | 1 |
| | | Colistin resistance gene *mcr*-1 in gut flora of children. | | | | | | | | | | | | | | 583 | 1 |
| | | Colistin resistance gene *mcr*-1 in gut flora of children. | | | | | | | | | | | | | | 1485 | 1 |
| | | Colistin resistance gene *mcr*-1 in gut flora of children. | | | | | | | | | | | | | | 3944 | 1 |
| | | Colistin resistance gene *mcr*-1 in gut flora of children. | | | | | | | | | | | | | | 773 | 1 |
| **9** | 10.1128/mBio.00543-17 | Novel Plasmid-Mediated Colistin Resistance Gene *mcr*-3 in *Escherichia col*. | 2015 | China | Asia | Swine | Fecal | P. *Escherichia coli* | | | 1 | mcr3 | 1 | 1 | IncHI2 | | |
| **10** | 10.1371/journal.pone.0172997 | *mcr*-1 identified in Avian Pathogenic *Escherichia coli* (APEC). | 2010 | Egypt | Africa | Poultry | N.A | P. *Escherichia coli* | 980 | 4 | | | 4 | | | | |
| | | *mcr*-1 identified in Avian Pathogenic *Escherichia coli* (APEC). | 2012-2015 | China | Asia | Poultry | N.A | P. *Escherichia coli* | | 8 | | | 8 | | | | |
| **11** | 10.1016/j.ijantimicag.2016.12.018 | Prevalence of the *mcr*-1 colistin resistance gene in extended-spectrum β-lactamase-producing *Escherichia coli* from human faecal samples collected in 2012 in rural villages in Shandong Province, China. | 2012 | China | Asia | Human | Fecal | P. *Escherichia coli* | 706 | 24 | | | 24 | | | | |
| **12** | 10.1016/S1473-3099(16)30527-8 | Prevalence, risk factors, outcomes, and molecular epidemiology of *mcr*-1-positive *Enterobacteriaceae* in patients and healthy adults from China: an epidemiological and clinical study. | 2007-2015 | China | Asia | Human | Fecal | P. *Escherichia coli* | 5332 | 76 | | | 76 | 13 | IncX4 | 116 | 55 |
| | | Prevalence, risk factors, outcomes, and molecular epidemiology of *mcr*-1-positive *Enterobacteriaceae* in patients and healthy adults from China: an epidemiological and clinical study. | 2007-2015 | China | Asia | Human | Fecal | *Klebsiella pneumoniae* | 348 | 13 | | | 13 | 14 | IncI2 | 602 | 1 |
| | | Prevalence, risk factors, outcomes, and molecular epidemiology of *mcr*-1-positive *Enterobacteriaceae* in patients and healthy adults from China: an epidemiological and clinical study. | 2007-2015 | China | Asia | Human | Fecal | *Enterobacter cloacae* | 890 | 1 | | | 1 | 7 | INCf | 101 | 1 |

| | | | | | | | | | | | | | | | | | |
|---|---|---|---|---|---|---|---|---|---|---|---|---|---|---|---|---|---|
| | | Prevalence, risk factors, outcomes, and molecular epidemiology of *mcr*-1-positive *Enterobacteriaceae* in patients and healthy adults from China: an epidemiological and clinical study. | 2007-2015 | China | Asia | Human | Fecal | *Enterobacter aerogenes* | 162 | 1 | | | 1 | 6 | IncFIB | 410 | 1 |
| | | Prevalence, risk factors, outcomes, and molecular epidemiology of *mcr*-1-positive *Enterobacteriaceae* in patients and healthy adults from China: an epidemiological and clinical study. | | | | | | | | | | | | 6 | Inck | 95 | 1 |
| | | Prevalence, risk factors, outcomes, and molecular epidemiology of *mcr*-1-positive *Enterobacteriaceae* in patients and healthy adults from China: an epidemiological and clinical study. | | | | | | | | | | | | | | 10 | 2 |
| | | Prevalence, risk factors, outcomes, and molecular epidemiology of *mcr*-1-positive *Enterobacteriaceae* in patients and healthy adults from China: an epidemiological and clinical study. | | | | | | | | | | | | | | 117 | 1 |
| | | Prevalence, risk factors, outcomes, and molecular epidemiology of *mcr*-1-positive *Enterobacteriaceae* in patients and healthy adults from China: an epidemiological and clinical study. | | | | | | | | | | | | | | 405 | 1 |
| | | Prevalence, risk factors, outcomes, and molecular epidemiology of *mcr*-1-positive *Enterobacteriaceae* in patients and healthy adults from China: an epidemiological and clinical study. | | | | | | | | | | | | | | 131 | 1 |
| | | Prevalence, risk factors, outcomes, and molecular epidemiology of *mcr*-1-positive *Enterobacteriaceae* in patients and healthy adults from China: an epidemiological and clinical study. | | | | | | | | | | | | | | 354 | 1 |
| | | Prevalence, risk factors, outcomes, and molecular epidemiology of *mcr*-1-positive *Enterobacteriaceae* in patients and healthy adults from China: an epidemiological and clinical study. | | | | | | | | | | | | | | 648 | 1 |
| | | Prevalence, risk factors, outcomes, and molecular epidemiology of *mcr*-1-positive *Enterobacteriaceae* in patients and healthy adults from China: an epidemiological and clinical study. | | | | | | | | | | | | | | 617 | 1 |

| | | | | | | | | | | | | | | | | | |
|---|---|---|---|---|---|---|---|---|---|---|---|---|---|---|---|---|---|
| | | Prevalence, risk factors, outcomes, and molecular epidemiology of *mcr*-1-positive *Enterobacteriaceae* in patients and healthy adults from China: an epidemiological and clinical study. | | | | | | | | | | | | | | 457 | 1 |
| | | Prevalence, risk factors, outcomes, and molecular epidemiology of *mcr*-1-positive *Enterobacteriaceae* in patients and healthy adults from China: an epidemiological and clinical study. | | | | | | | | | | | | | | 410 | 1 |
| | | Prevalence, risk factors, outcomes, and molecular epidemiology of *mcr*-1-positive *Enterobacteriaceae* in patients and healthy adults from China: an epidemiological and clinical study. | | | | | | | | | | | | | | 1193 | 1 |
| | | Prevalence, risk factors, outcomes, and molecular epidemiology of *mcr*-1-positive *Enterobacteriaceae* in patients and healthy adults from China: an epidemiological and clinical study. | | | | | | | | | | | | | | 354 | 1 |
| | | Prevalence, risk factors, outcomes, and molecular epidemiology of *mcr*-1-positive *Enterobacteriaceae* in patients and healthy adults from China: an epidemiological and clinical study. | | | | | | | | | | | | | | 156 | 1 |
| **13** | 10.1016/S1473-3099(16)30528-X | Prevalence of *mcr*-1 in *Escherichia coli* and *Klebsiella pneumoniae* recovered from bloodstream infections in China: a multicentre longitudinal study | 2013-2014 | China | Asia | Human | Blood | P. *Escherichia coli* | 1495 | 20 | | | 20 | 15 | IncI2 | 167 | 1 |
| | | Prevalence of *mcr*-1 in *Escherichia coli* and *Klebsiella pneumoniae* recovered from bloodstream infections in China: a multicentre longitudinal study | 2013-2014 | China | Asia | Human | Blood | *Klebsiella pneumoniae* | 571 | 1 | | | 1 | 1 | IncX4 | 10 | 2 |
| | | Prevalence of *mcr*-1 in *Escherichia coli* and *Klebsiella pneumoniae* recovered from bloodstream infections in China: a multicentre longitudinal study | | | | | | | | | | | | 4 | IncX4 | 2973 | 1 |
| | | Prevalence of *mcr*-1 in *Escherichia coli* and *Klebsiella pneumoniae* recovered from bloodstream infections in China: a multicentre longitudinal study | | | | | | | | | | | | | | 354 | 1 |
| | | Prevalence of *mcr*-1 in *Escherichia coli* and *Klebsiella pneumoniae* recovered from bloodstream infections in China: a multicentre longitudinal study | | | | | | | | | | | | | | 3028 | 1 |
| | | Prevalence of *mcr*-1 in *Escherichia coli* and *Klebsiella pneumoniae* recovered from bloodstream infections in China: a multicentre longitudinal study | | | | | | | | | | | | | | 354 | 1 |

| | | | | | | | | | | | | | | | | | |
|---|---|---|---|---|---|---|---|---|---|---|---|---|---|---|---|---|---|
| | | Prevalence of *mcr*-1 in *Escherichia coli* and *Klebsiella pneumoniae* recovered from bloodstream infections in China: a multicentre longitudinal study | | | | | | | | | | | | | | 156 | 1 |
| | | Prevalence of *mcr*-1 in *Escherichia coli* and *Klebsiella pneumoniae* recovered from bloodstream infections in China: a multicentre longitudinal study | | | | | | | | | | | | | | 1011 | 1 |
| | | Prevalence of *mcr*-1 in *Escherichia coli* and *Klebsiella pneumoniae* recovered from bloodstream infections in China: a multicentre longitudinal study | | | | | | | | | | | | | | 393 | 1 |
| | | Prevalence of *mcr*-1 in *Escherichia coli* and *Klebsiella pneumoniae* recovered from bloodstream infections in China: a multicentre longitudinal study | | | | | | | | | | | | | | 69 | 1 |
| | | Prevalence of *mcr*-1 in *Escherichia coli* and *Klebsiella pneumoniae* recovered from bloodstream infections in China: a multicentre longitudinal study | | | | | | | | | | | | | | 218 | 1 |
| | | Prevalence of *mcr*-1 in *Escherichia coli* and *Klebsiella pneumoniae* recovered from bloodstream infections in China: a multicentre longitudinal study | | | | | | | | | | | | | | 1193 | 1 |
| | | Prevalence of *mcr*-1 in *Escherichia coli* and *Klebsiella pneumoniae* recovered from bloodstream infections in China: a multicentre longitudinal study | | | | | | | | | | | | | | 853 | 1 |
| | | Prevalence of *mcr*-1 in *Escherichia coli* and *Klebsiella pneumoniae* recovered from bloodstream infections in China: a multicentre longitudinal study | | | | | | | | | | | | | | 58 | 1 |
| | | Prevalence of *mcr*-1 in *Escherichia coli* and *Klebsiella pneumoniae* recovered from bloodstream infections in China: a multicentre longitudinal study | | | | | | | | | | | | | | 44 | 1 |
| | | Prevalence of *mcr*-1 in *Escherichia coli* and *Klebsiella pneumoniae* recovered from bloodstream infections in China: a multicentre longitudinal study | | | | | | | | | | | | | | 131 | 1 |
| | | Prevalence of *mcr*-1 in *Escherichia coli* and *Klebsiella pneumoniae* recovered from bloodstream infections in China: a multicentre longitudinal study | | | | | | | | | | | | | | 117 | 2 |
| | | Prevalence of *mcr*-1 in *Escherichia coli* and *Klebsiella pneumoniae* recovered from bloodstream infections in China: a multicentre longitudinal study | | | | | | | | | | | | | | 457 | 1 |
| **14** | 10.1128/AAC.02623-16 | Detection of the *mcr*-1 Gene in a Multidrug-Resistant *Escherichia coli* Isolate from an Austrian Patient. | 2016 | Austria | Europe | Human | Fecal | P. *Escherichia coli* | | 1 | | | 1 | 1 | IncHI2 | 10 | 1 |

| | | | | | | | | | | | | | | | | | |
|---|---|---|---|---|---|---|---|---|---|---|---|---|---|---|---|---|---|
| **15** | 10.1093/jac/dkw533 | Spread of *mcr*-1-carrying *Enterobacteriaceae* in sewage water from Spain | 2013 | Spain | Europe | Water | Water | P. *Escherichia coli* | 195 | 29 | | | 29 | 29 | IncI2 | 1196 | 28 |
| | | Spread of *mcr*-1-carrying *Enterobacteriaceae* in sewage water from Spain | | | | | | | | | | | | | | 224 | 1 |
| | | Spread of *mcr*-1-carrying *Enterobacteriaceae* in sewage water from Spain | 2013 | Spain | Europe | Water | Water | *Klebsiella pneumoniae* | | 1 | | | 1 | 1 | IncI2 | | |
| **16** | 10.1016/j.ijantimicag.2016.11.010 | Detection of mcr-1-encoding plasmid-mediated colistin-resistant Salmonella isolates from human infection in Denmark. | 2008-2015 | Denmark | Europe | Human | Blood | *Salmonella enterica* serovar Typhimurum | 129 | 4 | | | 4 | 1 | IncI2 | | |
| | | Detection of mcr-1-encoding plasmid-mediated colistin-resistant Salmonella isolates from human infection in Denmark. | | | | | | | | | | | | 3 | IncX4 | | |
| **17** | 10.1093/jac/dkw411 | Genetic characterization of *mcr*-1-bearing plasmids to depict molecular mechanisms underlying dissemination of the colistin resistance determinant. | 2014-2015 | China | Asia | Swine | Fecal | P. *Escherichia coli* | 97 | 15 | | | 15 | | | | |
| | | Genetic characterization of *mcr*-1-bearing plasmids to depict molecular mechanisms underlying dissemination of the colistin resistance determinant | | | | | | | | | | | | 6 | IncX4 | | |
| | | Genetic characterization of *mcr*-1-bearing plasmids to depict molecular mechanisms underlying dissemination of the colistin resistance determinant | | | | | | | | | | | | 7 | IncI2 | | |
| | | Genetic characterization of *mcr*-1-bearing plasmids to depict molecular mechanisms underlying dissemination of the colistin resistance determinant | | | | | | | | | | | | 7 | IncHI2 | | |
| **18** | 10.1128/AAC.02347-16 | High Incidence of *Escherichia coli* Strains Coharboring *mcr*-1 and blaNDM from Chickens | 2015 | China | Asia | Poultry | Fecal | P. *Escherichia coli* | 78 | 53 | | | 53 | 53 | IncI2 | 2944 | 1 |
| | | High Incidence of *Escherichia coli* Strains Coharboring *mcr*-1 and blaNDM from Chickens | | | | | | | | | | | | | | 1011 | 1 |
| | | High Incidence of *Escherichia coli* Strains Coharboring *mcr*-1 and blaNDM from Chickens | | | | | | | | | | | | | | 10 | 1 |
| | | High Incidence of *Escherichia coli* Strains Coharboring *mcr*-1 and blaNDM from Chickens | | | | | | | | | | | | | | 101 | 1 |
| | | High Incidence of *Escherichia coli* Strains Coharboring *mcr*-1 and blaNDM from Chickens | | | | | | | | | | | | | | 117 | 2 |
| | | High Incidence of *Escherichia coli* Strains Coharboring *mcr*-1 and blaNDM from Chickens | | | | | | | | | | | | | | 2973 | 2 |

| | | | | | | | | | | | | | | | | | |
|---|---|---|---|---|---|---|---|---|---|---|---|---|---|---|---|---|---|
| | | High Incidence of *Escherichia coli* Strains Coharboring *mcr*-1 and blaNDM from Chickens | | | | | | | | | | | | | | 2847 | 2 |
| | | High Incidence of *Escherichia coli* Strains Coharboring *mcr*-1 and blaNDM from Chickens | | | | | | | | | | | | | | 297 | 5 |
| | | High Incidence of *Escherichia coli* Strains Coharboring *mcr*-1 and blaNDM from Chickens | | | | | | | | | | | | | | 156 | 5 |
| **19** | 10.1099/jmm.0.000425 | Detection and dissemination of the colistin resistance gene, mcr-1, from isolates and faecal samples in China. | 1998-2015 | China | Asia | Human | Fecal | P. *Escherichia coli* | 1161 | 3 | | | 3 | | | | |
| | | Detection and dissemination of the colistin resistance gene, mcr-1, from isolates and faecal samples in China. | 1998-2015 | China | Asia | Poultry | N.A | P. *Escherichia coli* | 981 | 45 | | | 45 | | | | |
| | | Detection and dissemination of the colistin resistance gene, mcr-1, from isolates and faecal samples in China. | 1998-2015 | China | Asia | Poultry | Chicken meat | P. *Escherichia coli* | 103 | 4 | | | 4 | | | | |
| | | Detection and dissemination of the colistin resistance gene, mcr-1, from isolates and faecal samples in China. | 1998-2015 | China | Asia | Water | Water | P. *Escherichia coli* | 9 | 2 | | | 2 | | | | |
| | | Detection and dissemination of the colistin resistance gene, mcr-1, from isolates and faecal samples in China. | 1998-2015 | China | Asia | Human | Fecal | P. *Escherichia coli* | 229 | 8 | | | 8 | | | | |
| | | Detection and dissemination of the colistin resistance gene, mcr-1, from isolates and faecal samples in China. | 1998-2015 | China | Asia | Human | Fecal | P. *Escherichia coli* | 108 | 6 | | | 6 | | | | |
| **20** | 10.1016/S1473-3099(16)00009-8 | Colistin resistance genemcr-1in extended-spectrum β-lactamase-producing andcarbapenemase-producingGram-negative bacteriain Germany | 2009 | Germany | Europe | Human | N.A | P. *Escherichia coli* | 557 | 1 | | | 1 | 1 | IncHI2 | | |
| | | Colistin resistance genemcr-1in extended-spectrum β-lactamase-producing andcarbapenemase-producingGram-negative bacteriain Germany | 2009 | Germany | Europe | Swine | N.A | P. *Escherichia coli* | | 3 | | | 3 | 3 | IncX4 | | |
| **21** | 10.1128/AAC.02167-16 | Various Sequence Types of Escherichia coli Isolates Coharboring blaNDM-5 and mcr-1 Genes from a Commercial Swine Farm in China. | 2015 | China | Asia | Swine | Fecal | P. *Escherichia coli* | 64 | 16 | | | 16 | 16 | IncX4 | 48 | 1 |
| | | Various Sequence Types of Escherichia coli Isolates Coharboring blaNDM-5 and mcr-1 Genes from a Commercial Swine Farm in China. | | | | | | | | | | | | | | 2439 | 1 |
| | | Various Sequence Types of Escherichia coli Isolates Coharboring blaNDM-5 and mcr-1 Genes from a Commercial Swine Farm in China. | | | | | | | | | | | | | | 1437 | 1 |
| | | Various Sequence Types of Escherichia coli Isolates Coharboring blaNDM-5 and mcr-1 Genes from a Commercial Swine Farm in China. | | | | | | | | | | | | | | 1178 | 1 |

| | | | | | | | | | | | | | | | | | |
|---|---|---|---|---|---|---|---|---|---|---|---|---|---|---|---|---|---|
| | | Various Sequence Types of Escherichia coli Isolates Coharboring blaNDM-5 and mcr-1 Genes from a Commercial Swine Farm in China. | | | | | | | | | | | | | | 165 | 1 |
| | | Various Sequence Types of Escherichia coli Isolates Coharboring blaNDM-5 and mcr-1 Genes from a Commercial Swine Farm in China. | | | | | | | | | | | | | | 3331 | 1 |
| | | Various Sequence Types of Escherichia coli Isolates Coharboring blaNDM-5 and mcr-1 Genes from a Commercial Swine Farm in China. | | | | | | | | | | | | | | 4436 | 1 |
| | | Various Sequence Types of Escherichia coli Isolates Coharboring blaNDM-5 and mcr-1 Genes from a Commercial Swine Farm in China. | | | | | | | | | | | | | | 54 | 1 |
| | | Various Sequence Types of Escherichia coli Isolates Coharboring blaNDM-5 and mcr-1 Genes from a Commercial Swine Farm in China. | | | | | | | | | | | | | | 156 | 1 |
| | | Various Sequence Types of Escherichia coli Isolates Coharboring blaNDM-5 and mcr-1 Genes from a Commercial Swine Farm in China. | | | | | | | | | | | | | | 4656 | 1 |
| | | Various Sequence Types of Escherichia coli Isolates Coharboring blaNDM-5 and mcr-1 Genes from a Commercial Swine Farm in China. | | | | | | | | | | | | | | 4429 | 1 |
| | | Various Sequence Types of Escherichia coli Isolates Coharboring blaNDM-5 and mcr-1 Genes from a Commercial Swine Farm in China. | | | | | | | | | | | | | | 90 | 1 |
| | | Various Sequence Types of Escherichia coli Isolates Coharboring blaNDM-5 and mcr-1 Genes from a Commercial Swine Farm in China. | | | | | | | | | | | | | | 167 | 1 |
| | | Various Sequence Types of Escherichia coli Isolates Coharboring blaNDM-5 and mcr-1 Genes from a Commercial Swine Farm in China. | | | | | | | | | | | | | | 410 | 1 |
| | | Various Sequence Types of Escherichia coli Isolates Coharboring blaNDM-5 and mcr-1 Genes from a Commercial Swine Farm in China. | | | | | | | | | | | | | | 1114 | 1 |
| **22** | 10.1016/j.ijantimicag.2016.10.003 | Detection of the high-risk clone ST131 of Escherichia coli carrying the colistin resistance genemcr-1 and causing acuteperitonitis | 2016 | Spain | Europe | Human | Periotneal fluid | P. *Escherichia coli* | | 1 | | | 1 | 1 | IncX | 131 | 1 |
| **23** | 10.1016/j.ijantimicag.2016.10.007 | Imported reptiles as a risk factor for theglobal distribution of Escherichia coli harbouring the colistin resistance gene mcr-1 | 2013-2014 | Vietnam | Asia | Reptiles | N.A | P. *Escherichia coli* | 142 | 2 | | | 2 | | | | |
| | | | | | | | | | | | | | | 1 | IncHI2 | 117 | 1 |

| | | | | | | | | | | | | | | | | | |
|---|---|---|---|---|---|---|---|---|---|---|---|---|---|---|---|---|---|
| | | | | | | | | | | | | | | 1 | Chromsomal | | 1011 |
| **24** | 10.1128/AAC.02229-16 | IncP Plasmid Carrying Colistin Resistance Gene mcr-1 in Klebsiella pneumoniae from Hospital Sewage. | 2015 | China | Asia | Water | Water | *Klebsiella pneumoniae* | | 1 | | | 1 | 1 | IncP | | |
| **25** | 10.1016/j.diagmicrobio.2016.11.005 | Emergence of plasmid-mediated colistin resistance and New Delhi metallo-β-lactamase genes in extensively drug-resistant *Escherichia coli* isolated from a patient in Thailand. | 2016 | Thailand | Asia | Human | Urine | P. *Escherichia coli* | | 1 | | | 1 | | | | |
| **26** | 10.1016/S1473-3099(16)00061-X | Early emergence of *mcr*-1 in *Escherichia coli* from food-producing animals | 1980-2014 | China | Asia | Poultry | N.A | P. *Escherichia coli* | 1611 | 104 | | | 104 | | | | |
| **27** | 10.1016/S1473-3099(16)00007-4 | Co-occurrence of extended spectrum β lactamase and *MCR*-1 encoding genes on plasmids | 2005-2014 | France | Europe | Bovine | Fecal | P. *Escherichia coli* | 517 | 106 | | | 106 | 106 | IncHI2 | | |
| **28** | 10.1016/S1473-3099(16)00014-1 | Colistin-resistant *Escherichia coli* harbouring *mcr*-1 isolated from food animals in Hanoi, Vietnam | 2014-2015 | Vietnam | Asia | Swine | Fecal | Non P. Escherichia coli | 7 | 6 | | | 6 | 6 | IncI2 | | |
| | | Colistin-resistant *Escherichia coli* harbouring *mcr*-1 isolated from food animals in Hanoi, Vietnam | 2014-2015 | Vietnam | Asia | Swine | N.A | Non P. Escherichia coli | 4 | 3 | | | 3 | 3 | IncI2 | | |
| **29** | 10.2807/1560-7917.ES.2016.21.6.30135 | Prevalence of *mcr*-1 in commensal Escherichia coli from French livestock, 2007 to 2014 | 2011 | France | Europe | Swine | Fecal | Non P. Escherichia coli | 200 | 1 | | | 1 | | | | |
| | | Prevalence of *mcr*-1 in commensal Escherichia coli from French livestock, 2007 to 2014 | 2013 | France | Europe | Swine | N.A | Non P. Escherichia coli | 196 | 1 | | | 1 | | | | |
| | | Prevalence of *mcr*-1 in commensal Escherichia coli from French livestock, 2007 to 2014 | 2013 | France | Europe | Poultry | N.A | Non P. Escherichia coli | 193 | 3 | | | 3 | | | | |
| | | Prevalence of *mcr*-1 in commensal Escherichia coli from French livestock, 2007 to 2014 | 2014 | France | Europe | Poultry | N.A | Non P. Escherichia coli | 239 | 14 | | | 14 | | | | |
| | | Prevalence of *mcr*-1 in commensal Escherichia coli from French livestock, 2007 to 2014 | 2014 | France | Europe | Poultry | N.A | Non P. Escherichia coli | 227 | 4 | | | 4 | | | | |
| **30** | 10.1016/S1473-3099(16)00012-8 | Colistin resistance gene *mcr*-1 harboured on a multidrug resistant plasmid | 2011-2012 | Belgium | Europe | Bovine | Fecal | P. *Escherichia coli* | 52 | 6 | | | 6 | 6 | IncFII | 100 | 2 |
| | | Colistin resistance gene *mcr*-1 harboured on a multidrug resistant plasmid | 2011-2012 | Belgium | Europe | Swine | Fecal | P. *Escherichia coli* | 53 | 7 | | | 7 | 7 | IncFII | 90 | 2 |
| | | Colistin resistance gene *mcr*-1 harboured on a multidrug resistant plasmid | | | | | | | | | | | | | | 10 | 9 |
| **31** | 10.1093/jac/dkx225 | MCR-2-mediated plasmid-borne polymyxin resistance most likely originates from *Moraxella pluranimalium* | 2017 | spain | Europe | Swine | Nose, Pleura, Peritoneal Cavity fluids | *Moraxella pluranimalium* | | 1 | 1 | mcr2.2 | 2 | 1 | Chromsomal | | |
| **32** | 10.1128/AAC.01444-16 | Emergence of Colistin Resistance Gene *mcr*-1 in Cronobacter sakazakii | 2015 | China | Asia | Poultry | Fecal | *Cronobacter sakazakii* | | 2 | | | 2 | 2 | IncI2 | | |

| | | | | | | | | | | | | | | | | | |
|---|---|---|---|---|---|---|---|---|---|---|---|---|---|---|---|---|---|
| | | Producing NDM-9 and in *Escherichia coli* from the Same Animal. | | | | | | | | | | | | | | | |
| **33** | 10.1128/AAC.02057-16 | Prevalence of Colistin Resistance Gene *mcr*-1 and Absence of *mcr*-2 in *Escherichia coli* Isolated from Healthy Food-Producing Animals in Japan. | 2000-2014 | Japan | Asia | Bovine | N.A | Non P. Escherichia coli | 3134 | 5 | | | 5 | 5 | IncI2 | | |
| | | Prevalence of Colistin Resistance Gene *mcr*-1 and Absence of *mcr*-2 in *Escherichia coli* Isolated from Healthy Food-Producing Animals in Japan. | 2000-2014 | Japan | Asia | Swine | N.A | Non P. Escherichia coli | 2052 | 20 | | | 20 | 20 | IncI2 | | |
| | | Prevalence of Colistin Resistance Gene *mcr*-1 and Absence of *mcr*-2 in *Escherichia coli* Isolated from Healthy Food-Producing Animals in Japan. | 2000-2014 | Japan | Asia | Poultry | N.A | Non P. Escherichia coli | 2017 | 14 | | | 14 | 14 | IncI2 | | |
| **34** | 10.1128/AAC.02244-16 | Prevalence of mcr-1 in the Cecal Contents of Food Animals in the United States. | 2016 | United States | North America | Swine | Fecal | P. *Escherichia coli* | | 2 | | | 2 | 2 | IncI2 | 234 | 1 |
| | | Prevalence of mcr-1 in the Cecal Contents of Food Animals in the United States. | | | | | | | | | | | | | | 132 | 1 |
| | | Prevalence of mcr-1 in the Cecal Contents of Food Animals in the United States. | | | | | | | | | | | | | | | |
| | | Prevalence of mcr-1 in the Cecal Contents of Food Animals in the United States. | | | | | | | | | | | | | | | |
| **35** | 10.1128/AAC.01165-16 | *Kluyvera ascorbata* Strain from Hospital Sewage Carrying the *mcr*-1 Colistin Resistance Gene. | 2015 | China | Asia | Water | Water | *Kluyvera ascorbata* | | 1 | | | 1 | 1 | IncI2 | | |
| **36** | 10.1016/j.ijantimicag.2016.09.003 | First report of the plasmid-mediated colistin resistance gene *mcr*-1 in a clinical *Escherichia coli* isolate in Algeria | 2011 | Algeria | Africa | Human | Sperm | P. *Escherichia coli* O29 | | 1 | | | 1 | 1 | IncFIB | 405 | 1 |
| **37** | 10.1016/j.ijantimicag.2016.04.004 | Emergence of *mcr*-1-mediated colistin resistance in *Escherichia coli* in Malaysia | 2009 | Malaysia | Asia | Poultry | Chicken liver | P. *Escherichia coli* | 15 | 1 | | | 1 | | | | |
| | | Emergence of *mcr*-1-mediated colistin resistance in *Escherichia coli* in Malaysia | 2009 | Malaysia | Asia | Poultry | Chicken liver and heart | P. *Escherichia coli* | 16 | 3 | | | 3 | | | | |
| | | Emergence of *mcr*-1-mediated colistin resistance in *Escherichia coli* in Malaysia | 2009 | Malaysia | Asia | Human | Urine | P. *Escherichia coli* | 95 | 1 | | | 1 | | | | |
| | | Emergence of *mcr*-1-mediated colistin resistance in *Escherichia coli* in Malaysia | 2009 | Malaysia | Asia | Water | Water | P. *Escherichia coli* | 5 | 2 | | | 2 | | | | |
| **38** | 10.15585/mmwr.mm6536e3 | Investigation of *Escherichia coli* Harboring the *mcr*-1 Resistance Gene - Connecticut, 2016. | 2016 | United States | North America | Human | Fecal | P. *Escherichia coli* O157 | | 1 | | | 1 | | | | |
| **39** | 10.15585/mmwr.mm6536e2 | Investigation of First Identified *mcr*-1 Gene in an Isolate from a U.S. Patient - Pennsylvania, 2016. | 2016 | United States | North America | Human | Urine | P. *Escherichia coli* | | 1 | | | 1 | | | | |

| | | | | | | | | | | | | | | | | | |
|---|---|---|---|---|---|---|---|---|---|---|---|---|---|---|---|---|---|
| **40** | 10.1017/S0950268816001369 | Colistin-resistant *Escherichia coli* clinical isolate harbouring the *mcr*-1 gene in ecuador | 2016 | Ecuador | South America | Human | Sperm | P. *Escherichia coli* | | 1 | | | 1 | | | 609 | 1 |
| **41** | 10.1128/mBio.01191-16 | Colistin- and Carbapenem-Resistant *Escherichia coli* Harboring *mcr*-1 and blaNDM-5, Causing a Complicated Urinary Tract Infection in a Patient from the United States. | 2014 | United States | North America | Human | Urine | P. *Escherichia coli* | | 1 | | | 1 | 1 | IncX4 | 405 | 1 |
| **42** | 10.1128/AAC.00443-16 | Plasmid with Colistin Resistance Gene *mcr*-1 in Extended-Spectrum-β-Lactamase-Producing *Escherichia coli* Strains Isolated from Pig Slurry in Estonia. | 2011-2014 | Estonia | Europe | Swine | Fecal | P. *Escherichia coli* | 88 | 3 | | | 3 | 3 | IncX4 | | |
| **43** | 10.1016/j.ijid.2016.07.007 | Plasmid-mediated colistin resistance in *Escherichia coli* from the Arabian Peninsula. | 2015 | Bahrain | Asia | Human | N.A | P. *Escherichia coli* | | 1 | | | 1 | 1 | IncI2 | 648 | 1 |
| | | Plasmid-mediated colistin resistance in *Escherichia coli* from the Arabian Peninsula. | 2015 | Bahrain | Asia | Human | N.A | P. *Escherichia coli* | | 1 | | | 1 | 1 | IncI2 | 224 | 1 |
| | | Plasmid-mediated colistin resistance in *Escherichia coli* from the Arabian Peninsula. | 2012 | Saudi Arabia | Asia | Human | N.A | P. *Escherichia coli* | | 1 | | | 1 | 1 | IncHI2 | 68 | 1 |
| | | Plasmid-mediated colistin resistance in *Escherichia coli* from the Arabian Peninsula. | 2013 | United Arab Emirates | Asia | Human | N.A | P. *Escherichia coli* | | 1 | | | 1 | 1 | IncI2 | 131 | 1 |
| **44** | 10.1093/jac/dkw328 | Detection of the plasmid-mediated colistin-resistance gene *mcr*-1 in faecal metagenomes of Dutch travellers. | 2010-2012 | Netherland | Europe | Human | Fecal | P. *Escherichia coli* | 6 | 6 | | | 6 | | | 1011 | 1 |
| | | Detection of the plasmid-mediated colistin-resistance gene *mcr*-1 in faecal metagenomes of Dutch travellers. | | | | | | | | | | | | | | 744 | 1 |
| | | Detection of the plasmid-mediated colistin-resistance gene *mcr*-1 in faecal metagenomes of Dutch travellers. | | | | | | | | | | | | | | 80 | 1 |
| **45** | 10.1038/emi.2016.85 | Transmissible colistin resistance encoded by *mcr*-1 detected in clinical *Enterobacteriaceae* isolates in Singapore | 2016 | Singapore | Asia | Human | Urine | P. *Escherichia coli* | 166 | 2 | | | 2 | | | 460 | 1 |
| | | Transmissible colistin resistance encoded by *mcr*-1 detected in clinical *Enterobacteriaceae* isolates in Singapore | | | | | | | | | | | | | | 156 | 1 |
| **46** | 10.1016/S1473-3099(15)00540-X | Dissemination of the *mcr-1* colistin resistance gene (corrospondance 1) | 2016 | Laos | Asia | Human | Fecal | P. *Escherichia coli* | 6 | 6 | | | 6 | | | | |
| | | Dissemination of the *mcr-1* colistin resistance gene (corrospondance 1) | 2016 | Laos | Asia | Swine | Fecal | P. *Escherichia coli* | 4 | 3 | | | 3 | | | | |
| | | Dissemination of the *mcr-1* colistin resistance gene (corrospondance 1) | 2016 | Thailand | Asia | Human | Fecal | P. *Escherichia coli* | 3 | 2 | | | 2 | | | | |

| | | | | | | | | | | | | | | | | | |
|---|---|---|---|---|---|---|---|---|---|---|---|---|---|---|---|---|---|
| | | Dissemination of the *mcr-1* colistin resistance gene (corrospondance 1) | 2016 | Algeria | Africa | Poultry | Fecal | P. *Escherichia coli* | 2 | 1 | | | 1 | | | | |
| **47** | 10.1016/j.ijantimicag.2016.06.021 | Characterisation of multidrug-resistant Shiga toxin-producing Escherichia coli cultured from pigs in China: co-occurrence of extended-spectrum β-lactamase- and mcr-1-encoding genes on plasmids. | 2011-2012 | China | Asia | Swine | Fecal | P. *Escherichia coli* | 93 | 10 | | | 10 | | | | |
| **48** | 10.1128/AAC.01325-16 | First Report of the Globally Disseminated IncX4 Plasmid Carrying the mcr-1 Gene in a Colistin-Resistant Escherichia coli Sequence Type 101 Isolate from a Human Infection in brazil | 2016 | Brazil | South America | Human | Wound | P. *Escherichia coli* | | 1 | | | 1 | 1 | IncX4 | 101 | 1 |
| **49** | 10.1128/AAC.01319-16 | Coexistence of mcr-1 and blaNDM-1 in Escherichia coli from Venezuela. | 2015 | Venezuela | South America | Human | Fecal | P. *Escherichia coli* | 16 | 1 | | | 1 | 1 | IncI2 | 19 | 1 |
| | | Coexistence of mcr-1 and blaNDM-1 in Escherichia coli from Venezuela. | 2015 | Venezuela | South America | Swine | Fecal | P. *Escherichia coli* | 17 | 1 | | | 1 | 1 | IncI2 | 452 | 1 |
| **50** | 10.2807/1560-7917.ES.2016.21.26.30267 | Letter to the editor: Escherichia coli harbouring mcr-1 gene isolated from poultry not exposed to polymyxins in Brazil | 2015 | Brazil | South America | Poultry | Fecal | Non P. Escherichia coli | 10 | 10 | | | 10 | | | | |
| | | Letter to the editor: Escherichia coli harbouring mcr-1 gene isolated from poultry not exposed to polymyxins in Brazil | | | | | | | | | | | | | | | |
| **51** | 10.1016/j.ijantimicag.2016.06.001 | Plasmid-mediated colistin-resistant Escherichia coli detected from 2014 in Norway. | 2006-2015 | Norway | Europe | Human | N.A | P. *Escherichia coli* | 4951 | 1 | | | 1 | 1 | IncI2 | | |
| **52** | 10.1128/mBio.00177-16 | Diversified mcr-1-Harbouring Plasmid Reservoirs Confer Resistance to Colistin in Human Gut Microbiota | 2015 | China | Asia | Human | Fecal | P. *Escherichia coli* | 48 | 3 | | | 3 | 3 | IncI2 | 40 | 1 |
| | | Diversified mcr-1-Harbouring Plasmid Reservoirs Confer Resistance to Colistin in Human Gut Microbiota | | | | | | | | | | | | | | 642 | 1 |
| | | Diversified mcr-1-Harbouring Plasmid Reservoirs Confer Resistance to Colistin in Human Gut Microbiota | | | | | | | | | | | | | | 648 | 1 |
| **53** | 10.1128/AAC.00731-16 | Travelers Can Import Colistin-Resistant *Enterobacteriaceae*, Including Those Possessing the Plasmid-Mediated *mcr-1* Gene | 2015 | Switzerland | Europe | Human | Fecal | P. *Escherichia coli* | | 3 | | | 3 | 3 | IncHI2 | 10 | 3 |
| **54** | 10.1093/jac/dkw173 | Inducible colistin resistance via a disrupted plasmid-borne mcr-1 gene in a 2008 Vietnamese Shigella sonnei isolate. | 2008 | Vietnam | Asia | Human | Fecal | *Shigella sonnei* | | 1 | | | 1 | 1 | IncI2 | | |
| **55** | 10.1128/AAC.00440-16 | Detection of the mcr-1 Colistin Resistance Gene in Carbapenem-Resistant Enterobacteriaceae from Different Hospitals in China. | 2014-2015 | China | Asia | Human | N.A | P. *Escherichia coli* | 36 | | | | | | | | |
| | | Detection of the mcr-1 Colistin Resistance Gene in Carbapenem- | 2014-2015 | China | Asia | Human | Urine | P. *Escherichia coli* | | 1 | | | 1 | 1 | IncI2 | 167 | 1 |

| | | | | | | | | | | | | | | | | | |
|---|---|---|---|---|---|---|---|---|---|---|---|---|---|---|---|---|---|
| | | Resistant Enterobacteriaceae from Different Hospitals in China. | | | | | | | | | | | | | | | |
| | | Detection of the mcr-1 Colistin Resistance Gene in Carbapenem-Resistant Enterobacteriaceae from Different Hospitals in China. | 2014-2015 | China | Asia | Human | Ascites | P. *Escherichia coli* | | 1 | | | 1 | 1 | IncX4 | 156 | 1 |
| | | Detection of the mcr-1 Colistin Resistance Gene in Carbapenem-Resistant Enterobacteriaceae from Different Hospitals in China. | 2014-2015 | China | Asia | Human | Bile | P. *Escherichia coli* | | 1 | | | 1 | 1 | Chromsomal | 457 | 1 |
| **56** | 10.2807/1560-7917.ES.2016.21.17.30214 | Silent dissemination of colistin-resistant Escherichia coli in South America could contribute to the global spread of the mcr-1 gene. | 2012 | Brazil | South America | Swine | Fecal | P. *Escherichia coli* | 113 | 2 | | | 2 | | | | |
| | | Silent dissemination of colistin-resistant Escherichia coli in South America could contribute to the global spread of the mcr-1 gene. | 2013 | Brazil | South America | Poultry | Fecal | P. *Escherichia coli* | 79 | 14 | | | 14 | | | | |
| **57** | 10.1128/AAC.00444-16 | Genetic Features of MCR-1-Producing Colistin-Resistant Escherichia coli Isolates in South Africa. | 2014-2015 | South Africa | Africa | Human | Blood, Pus, Urine, Wound | P. *Escherichia coli* | | 7 | | | 7 | 4 | IncI2 | 10 | 1 |
| | | Genetic Features of MCR-1-Producing Colistin-Resistant Escherichia coli Isolates in South Africa. | | | | | | | | | | | | 1 | IncHI2 | 1007 | 1 |
| | | Genetic Features of MCR-1-Producing Colistin-Resistant Escherichia coli Isolates in South Africa. | | | | | | | | | | | | 1 | IncX4 | 624 | 1 |
| | | Genetic Features of MCR-1-Producing Colistin-Resistant Escherichia coli Isolates in South Africa. | | | | | | | | | | | | | | 57 | 1 |
| | | Genetic Features of MCR-1-Producing Colistin-Resistant Escherichia coli Isolates in South Africa. | | | | | | | | | | | | | | 101 | 1 |
| | | Genetic Features of MCR-1-Producing Colistin-Resistant Escherichia coli Isolates in South Africa. | | | | | | | | | | | | | | 624 | 1 |
| | | Genetic Features of MCR-1-Producing Colistin-Resistant Escherichia coli Isolates in South Africa. | | | | | | | | | | | | | | 226 | 1 |
| **58** | 10.1093/jac/dkw149 | Colistin resistance in Salmonella and Escherichia coli isolates from a pig farm in Great Britain. | 2016 | United Kingdom | Europe | Swine | Small intestine sample | *Salmonella enterica* serovar Typhimurim variant copenhagen | | 1 | | | 1 | 1 | IncI2 | | |
| | | Colistin resistance in Salmonella and Escherichia coli isolates from a pig farm in Great Britain. | 2016 | United Kingdom | Europe | Swine | Small intestine sample | P. *Escherichia coli* | | 1 | | | 1 | 1 | IncI2 | | |
| **59** | 10.1093/jac/dkw093 | Detection of the plasmid-mediated mcr-1 gene conferring colistin resistance in human and food isolates | 2012-2015 | United Kingdom | Europe | Human | Fecal | *Salmonella enterica* serovar Typhimurium | 17195 | 2 | | | 2 | 2 | IncX4 | | |

| | | | | | | | | | | | | | | | | | |
|---|---|---|---|---|---|---|---|---|---|---|---|---|---|---|---|---|---|
| | | of Salmonella enterica and Escherichia coli in England and Wales. | | | | | | | | | | | | | | | |
| | | Detection of the plasmid-mediated mcr-1 gene conferring colistin resistance in human and food isolates of Salmonella enterica and Escherichia coli in England and Wales. | 2012-2015 | United Kingdom | Europe | Human | Fecal | *Salmonella enterica* serovar Paratyphi B var Java | | 4 | | | 4 | 4 | IncI2 | | |
| | | Detection of the plasmid-mediated mcr-1 gene conferring colistin resistance in human and food isolates of Salmonella enterica and Escherichia coli in England and Wales. | 2012-2015 | United Kingdom | Europe | Human | Fecal | *Salmonella enterica* serovar Virchow | | 3 | | | 3 | 3 | IncHI2 | | |
| | | Detection of the plasmid-mediated mcr-1 gene conferring colistin resistance in human and food isolates of Salmonella enterica and Escherichia coli in England and Wales. | 2012-2015 | United Kingdom | Europe | Poultry | Chicken meat | *Salmonella enterica* serovar Paratyphi B var Java phage type Colindale | 489 | 2 | | | 2 | 2 | IncHI2 | | |
| | | Detection of the plasmid-mediated mcr-1 gene conferring colistin resistance in human and food isolates of Salmonella enterica and Escherichia coli in England and Wales. | 2012-2015 | United Kingdom | Europe | Human | Fecal | P. *Escherichia coli* | 2656 | 3 | | | 3 | 3 | IncI2 | 457 | 3 |
| **60** | 10.1016/j.rvsc. 2016.02.003 | Detection of plasmid mediated colistin resistance (MCR-1) in Escherichia coli and Salmonella enterica isolated from poultry and swine in Spain | 2009-2011 | spain | Europe | Poultry | Fecal | P. *Escherichia coli* | | 3 | | | 3 | 3 | IncI2 | | |
| | | Detection of plasmid mediated colistin resistance (MCR-1) in Escherichia coli and Salmonella enterica isolated from poultry and swine in Spain | 2009-2011 | spain | Europe | Swine | Fecal | P. *Escherichia coli* | | 2 | | | 2 | 2 | IncI2 | | |
| | | Detection of plasmid mediated colistin resistance (MCR-1) in Escherichia coli and Salmonella enterica isolated from poultry and swine in Spain | 2009-2011 | spain | Europe | Swine | Fecal | *Salmonella enterica* serovar Typhimurium | | 1 | | | 1 | 1 | IncI2 | | |
| | | Detection of plasmid mediated colistin resistance (MCR-1) in Escherichia coli and Salmonella enterica isolated from poultry and swine in Spain | 2009-2011 | spain | Europe | Swine | Fecal | *Salmonella enterica* serovar Typhimurium | | 2 | | | 2 | 2 | IncI2 | | |
| | | Detection of plasmid mediated colistin resistance (MCR-1) in Escherichia coli and Salmonella enterica isolated from poultry and swine in Spain | 2009-2011 | spain | Europe | Swine | Fecal | *Salmonella enterica* serovar Rissen | | 1 | | | 1 | 1 | IncI2 | | |
| **61** | 10.7196/SAMJ. 2016.v106i5.10 710 | Emergence of plasmid-mediated colistin resistance (MCR-1) among Escherichia coli isolated from South African patients | 2014-2016 | South Africa | Africa | Human | Blood, Pus, Urine, Wound | P. *Escherichia coli* | | 9 | | | 9 | | | | |
| **62** | 10.2807/1560-7917.ES.2016.2 1.13.30183 | Detection of mcr-1 colistin resistance gene in polyclonal Escherichia coli isolates in Barcelona, Spain, 2012 to 2015. | 2012-2015 | spain | Europe | Human | Fecal | P. *Escherichia coli* | 53 | 15 | | | 15 | | | | |

| | | | | | | | | | | | | | | | | | |
|---|---|---|---|---|---|---|---|---|---|---|---|---|---|---|---|---|---|
| **63** | 10.2807/1560-7917.ES.2016.21.8.30144 | Impact of food animal trade on the spread of mcr-1-mediated colistin resistance, Tunisia, July 2015. | 2015 | Tunisia | Africa | Poultry | Fecal | P. *Escherichia coli* | 37 | 37 | | | 37 | 37 | IncHI2 | 4 | 37 |
| **64** | 10.2807/1560-7917.ES.2016.21.9.30149 | Presence of mcr-1-positive Enterobacteriaceae in retail chicken meat but not in humans in the Netherlands since 2009 | 2009-2014 | Netherland | Europe | Poultry | Chicken meat | P. *Escherichia coli* | 196 | 3 | | | 3 | | | | |
| **65** | 10.1128/AAC.00066-16. Print 2016 Apr | Occurrence of the Plasmid-Borne mcr-1 Colistin Resistance Gene in Extended-Spectrum-β-Lactamase-Producing Enterobacteriaceae in River Water and Imported Vegetable Samples in Switzerland | 2012 | Switzerland | Europe | Water | Water | Non P. Escherichia coli | 74 | 1 | | | 1 | | | 359 | 1 |
| | | Occurrence of the Plasmid-Borne mcr-1 Colistin Resistance Gene in Extended-Spectrum-β-Lactamase-Producing Enterobacteriaceae in River Water and Imported Vegetable Samples in Switzerland | 2012 | Switzerland | Europe | Vegetables | N.A | Non P. Escherichia coli | 60 | 1 | | | 1 | | | 167 | 1 |
| | | Occurrence of the Plasmid-Borne mcr-1 Colistin Resistance Gene in Extended-Spectrum-β-Lactamase-Producing Enterobacteriaceae in River Water and Imported Vegetable Samples in Switzerland | 2012 | Switzerland | Europe | Vegetables | N.A | Non P. Escherichia coli | | 1 | | | 1 | | | 4683 | 1 |
| **66** | 10.1016/j.ijantimicag.2016.02.011 | Characterisation of the plasmid-mediated colistin resistance gene mcr-1 in Escherichia coli isolated from animals in Egypt | 2016 | Egypt | Africa | Bovine | Blood | P. *Escherichia coli* | 38 | 1 | | | 1 | 1 | IncHI2 | 10 | 1 |
| **67** | 10.1016/S1473-3099(16)00067-0 | Dissemination of the *mcr-1* colistin resistance gene (corrospondance 2) | 2011 | Canada | North America | Human | N.A | P. *Escherichia coli* | 1600 | 1 | | | 1 | 1 | IncI2 | | |
| | | Dissemination of the *mcr-1* colistin resistance gene (corrospondance 2) | 2010 | Canada | North America | Bovine | Ground beef | P. *Escherichia coli* | | 2 | | | 2 | 2 | IncHI2A | | |
| **68** | 10.1016/s1473-3099(16)00533-2 | Dissemination of the *mcr-1* colistin resistance gene (corrospondance 3) | 2015 | China | Asia | Human | N.A | P. *Escherichia coli* | 368 | 27 | | | 27 | | | | |
| **69** | 10.1016/s1473-3099(16)00062-1 | Dissemination of the *mcr-1* colistin resistance gene (corrospondance 4) | 2015 | China | Asia | Human | Fecal | P. *Escherichia coli* | 234 | 5 | | | 5 | | | | |
| **70** | 10.1016/S1473-3099(16)00057-8 | Carbapenem-resistant and colistin-resistant Escherichia coli co-producing NDM-9 and MCR-1 | 2014 | China | Asia | Poultry | Chicken meat | P. *Escherichia coli* | 1 | 1 | | | 1 | 1 | IncI2 | 167 | 1 |
| **71** | 10.1016/S1473-3099(16)00056-6 | Emergence of the mcr-1 colistin resistance gene in carbapenem-resistant Enterobacteriaceae | 2013-2015 | China | Asia | Human | Blood | P. *Escherichia coli* | 2 | 2 | | | 2 | | | | |
| | | Emergence of the mcr-1 colistin resistance gene in carbapenem-resistant Enterobacteriaceae | 2013-2015 | China | Asia | Human | Surgery wound, and drainage fluid | *Klebsiella pneumoniae* | 4 | 2 | | | 2 | | | | |

| | | | | | | | | | | | | | | | | | |
|---|---|---|---|---|---|---|---|---|---|---|---|---|---|---|---|---|---|
| **72** | 10.1016/S1473-3099(16)00010-4 | Colistin resistance genemcr-1 and pHNSHP45plasmid in humanisolates of Escherichiacoli and Klebsiellapneumoniae | 2012 | Cambodia | Asia | Human | Fecal | P. *Escherichia coli* | 1100 | 1 | | | 1 | | | 354 | 1 |
| **73** | 10.3389/fmicb.2017.02094 | Remarkable Diversity of Escherichia coli Carrying mcr-1 from Hospital Sewage with the Identification of Two New mcr-1 Variants | 2015 | China | Asia | Water | Water | P. *Escherichia coli* | 9 | | | | | | | 10 | 1 |
| | | Remarkable Diversity of Escherichia coli Carrying mcr-1 from Hospital Sewage with the Identification of Two New mcr-1 Variants | 2015 | China | Asia | Water | Water | P. *Escherichia coli* | | 2 | | | 2 | 2 | Chromsomal | 34 | 1 |
| | | Remarkable Diversity of Escherichia coli Carrying mcr-1 from Hospital Sewage with the Identification of Two New mcr-1 Variants | 2015 | China | Asia | Water | Water | P. *Escherichia coli* | | 5 | | mcr1.1 | 5 | 2 | IncX4 | 48 | 1 |
| | | Remarkable Diversity of Escherichia coli Carrying mcr-1 from Hospital Sewage with the Identification of Two New mcr-1 Variants | 2015 | China | Asia | Water | Water | P. *Escherichia coli* | | 1 | | mcr1.4 | 1 | 1 | IncI2 | 1196 | 1 |
| | | Remarkable Diversity of Escherichia coli Carrying mcr-1 from Hospital Sewage with the Identification of Two New mcr-1 Variants | 2015 | China | Asia | Water | Water | P. *Escherichia coli* | | 1 | | mcr1.7 | 1 | 1 | IncP | 7086 | 1 |
| | | Remarkable Diversity of Escherichia coli Carrying mcr-1 from Hospital Sewage with the Identification of Two New mcr-1 Variants | 2015 | China | Asia | Water | Water | P. *Escherichia coli* | | | | | | 1 | IncHI2 | 7087 | 1 |
| **74** | 10.2807/1560-7917.ES.2017.22.31.30586 | Co-occurrence of colistin-resistance genes mcr-1 and mcr-3 among multidrug-resistant Escherichia coli isolated from cattle, Spain, September 2015 | 2015 | spain | Europe | Bovine | Fecal | P. *Escherichia coli* | 77 | 5 | 1 | mcr3 | 6 | 5 | IncHI2 | 533 | 1 |
| **75** | 10.1016/j.vetmic.2017.08.024 | Complete genetic analysis of a Salmonella enterica serovar Indiana isolate accompanying four plasmids carrying mcr-1, ESBL and other resistance genes in China. | 2012 | China | Asia | Poultry | N.A | *Salmonella enterica* serovar Indiana | 4 | 1 | | | 1 | 1 | IncI2 | | |
| **76** | 10.1093/jac/dkx327 | Identification of a novel transposon-associated phosphoethanolamine transferase gene, mcr-5, conferring colistin resistance in d-tartrate fermenting Salmonella enterica subsp. enterica serovar Paratyphi B. | 2011-2016 | Germany | Europe | Poultry | Chicken meat | *Salmonella enterica* serovar Paratyphi B | | | 3 | mcr5 | 3 | 3 | ColE | | |
| | | Identification of a novel transposon-associated phosphoethanolamine transferase gene, mcr-5, conferring colistin resistance in d-tartrate fermenting Salmonella enterica subsp. enterica serovar Paratyphi B. | 2011-2016 | Germany | Europe | Water | Water | *Salmonella enterica* serovar Paratyphi B | | | 4 | mcr5 | 4 | 4 | ColE | | |
| | | Identification of a novel transposon-associated phosphoethanolamine transferase gene, mcr-5, conferring colistin resistance in d-tartrate | 2011-2016 | Germany | Europe | Poultry | N.A | *Salmonella enterica* serovar Paratyphi B | | | 7 | mcr5 | 7 | 7 | ColE | | |

| | | | | | | | | | | | | | | | | | |
|---|---|---|---|---|---|---|---|---|---|---|---|---|---|---|---|---|---|
| | | fermenting Salmonella enterica subsp. enterica serovar Paratyphi B. | | | | | | | | | | | | | | | |
| **77** | 10.1016/j.jgar.2017.10.012 | Detection of the colistin resistance gene mcr-1 in avian pathogenic Escherichia coli in Pakistan | 2017 | Pakistan | Asia | Poultry | Carcass | P. *Escherichia coli* | 10 | 1 | | | 1 | | IncX4 | 135 | |
| **78** | 10.3201/eid2312.170883 | High Rate of MCR-1–Producing Escherichia coli and Klebsiella pneumoniae among Pigs, Portugal | 2016 | Portugal | Europe | Swine | Fecal | P. *Escherichia coli* | 90 | 90 | | | 90 | 48 | IncHI2 | 10 | 21 |
| | | High Rate of MCR-1–Producing Escherichia coli and Klebsiella pneumoniae among Pigs, Portugal | | | | | | | | | | | | 34 | IncP | 23 | 9 |
| | | High Rate of MCR-1–Producing Escherichia coli and Klebsiella pneumoniae among Pigs, Portugal | | | | | | | | | | | | 8 | IncX4 | 38 | 1 |
| | | High Rate of MCR-1–Producing Escherichia coli and Klebsiella pneumoniae among Pigs, Portugal | 2016 | Portugal | Europe | Swine | N.A | *Klebsiella pneumoniae* | 17 | 8 | | | 8 | 4 | IncHI2 | 46 | 1 |
| | | High Rate of MCR-1–Producing Escherichia coli and Klebsiella pneumoniae among Pigs, Portugal | | | | | | | | | | | | | | 101 | 4 |
| | | High Rate of MCR-1–Producing Escherichia coli and Klebsiella pneumoniae among Pigs, Portugal | | | | | | | | | | | | | | 156 | 5 |
| | | High Rate of MCR-1–Producing Escherichia coli and Klebsiella pneumoniae among Pigs, Portugal | | | | | | | | | | | | | | 6453 | 13 |
| | | High Rate of MCR-1–Producing Escherichia coli and Klebsiella pneumoniae among Pigs, Portugal | | | | | | | | | | | | | | 45 | 6 |
| | | High Rate of MCR-1–Producing Escherichia coli and Klebsiella pneumoniae among Pigs, Portugal | | | | | | | | | | | | | | 1563 | 2 |
| **79** | 10.1016/s1473-3099(15)00063-3 | Dissemination of the mcr-1 colistin resistance gene (corrospondance 5) | | China | Asia | Poultry | | *Salmonella enterica* | 27 | | | | | | | | |
| | | Dissemination of the mcr-1 colistin resistance gene (corrospondance 5) | 2012 | China | Asia | Poultry | Chicken meat | *Salmonella enterica* seorvar Derby | | 1 | | | 1 | 1 | IncP | | |
| | | Dissemination of the mcr-1 colistin resistance gene (corrospondance 5) | 2012 | China | Asia | Poultry | Chicken meat | *Salmonella enterica* serovar Paratyphi B | | 1 | | | 1 | 1 | IncX4 | | |
| | | Dissemination of the mcr-1 colistin resistance gene (corrospondance 5) | 2012 | China | Asia | Poultry | Chicken meat | *Salmonella enterica* serovar Paratyphi B | | 1 | | | 1 | 1 | IncX4 | | |
| | | Dissemination of the mcr-1 colistin resistance gene (corrospondance 5) | 2013 | China | Asia | Poultry | N.A | *Salmonella enterica* serovar 1,4,[5],12:i:- | | 1 | | | 1 | 1 | IncP | | |
| **80** | 10.1016/s1473-3099(16)00532-0 | Dissemination of the mcr-1 colistin resistance gene (corrospondance 6) | 2011-2012 | Portugal | Europe | Bovine | Meat by product | *Salmonella enterica* | 258 | 1 | | | 1 | 1 | IncHI2 | | |

| | | | | | | | | | | | | | | | | | |
|---|---|---|---|---|---|---|---|---|---|---|---|---|---|---|---|---|---|
| | | | | | | | | serovar Typhimurium | | | | | | | | | |
| **81** | 10.1128/AAC.00269-16 | Emergence of Plasmid-Mediated Colistin Resistance Gene mcr-1 in aClinical Escherichia coli Isolate from Egypt | 2016 | Egypt | Africa | Human | Sputum | P. *Escherichia coli* | 241 | 1 | | | 1 | | | 1011 | 1 |
| **82** | 10.1093/jac/dkw122 | Colistin resistance gene mcr-1 in Escherichia coli isolates from humans and retail meats, Taiwan | 2010 | China | Asia | Human | Fecal | P. *Escherichia coli* | 1136 | 1 | | | 1 | | | 38 | 8 |
| | | Colistin resistance gene mcr-1 in Escherichia coli isolates from humans and retail meats, Taiwan | 2012 | China | Asia | Human | N.A | P. *Escherichia coli* | 1752 | 3 | | | 3 | | | 117 | 5 |
| | | Colistin resistance gene mcr-1 in Escherichia coli isolates from humans and retail meats, Taiwan | 2014 | China | Asia | Human | N.A | P. *Escherichia coli* | 1701 | 11 | | | 11 | | | 701 | 1 |
| | | Colistin resistance gene mcr-1 in Escherichia coli isolates from humans and retail meats, Taiwan | 2012 | China | Asia | Bovine | Beef | P. *Escherichia coli* | 89 | 1 | | | 1 | | | 7044 | 1 |
| | | Colistin resistance gene mcr-1 in Escherichia coli isolates from humans and retail meats, Taiwan | 2013 | China | Asia | Poultry | Chicken meat | P. *Escherichia coli* | 91 | 6 | | | 6 | | | 428 | 1 |
| | | Colistin resistance gene mcr-1 in Escherichia coli isolates from humans and retail meats, Taiwan | 2015 | China | Asia | Swine | Pork | P. *Escherichia coli* | 126 | 11 | | | 11 | | | | |
| **83** | 10.1016/j.ijfoodmicro.2017.10.007 | New insights into resistance to colistin and third-generation cephalosporinsofEscherichia coliin poultry, Portugal: NovelblaCTX-M-166andblaESACgenes | 2014 | Portugal | Europe | Poultry | Fecal | P. *Escherichia coli* | 185 | 2 | | | 2 | | | | |
| **84** | 10.1016/j.vetmic.2017.11.014 | *mcr*-1-like detection in commensal Escherichia coli and Salmonella spp. fromfood-producing animals at slaughter in Europe | 2008-2014 | France | Europe | Swine | Fecal | Non P. Escherichia coli | | 9 | | | 9 | | | | |
| | | *mcr*-1-like detection in commensal Escherichia coli and Salmonella spp. fromfood-producing animals at slaughter in Europe | 2008-2014 | France | Europe | Poultry | Fecal | Non P. Escherichia coli | 4 | 2 | | | 2 | | | | |
| | | *mcr*-1-like detection in commensal Escherichia coli and Salmonella spp. fromfood-producing animals at slaughter in Europe | 2008-2014 | Germany | Europe | Poultry | Fecal | Non P. Escherichia coli | 58 | 38 | | | 38 | | | | |
| | | *mcr*-1-like detection in commensal Escherichia coli and Salmonella spp. fromfood-producing animals at slaughter in Europe | 2008-2014 | Spain | Europe | Swine | Fecal | Non P. Escherichia coli | 47 | 23 | | | 23 | | | | |
| | | *mcr*-1-like detection in commensal Escherichia coli and Salmonella spp. fromfood-producing animals at slaughter in Europe | 2008-2014 | Netherland | Europe | Poultry | Fecal | Non P. Escherichia coli | 5 | 5 | | | 5 | | | | |
| **85** | 10.1128/AAC.00841-17 | Genomic and Molecular Characterizationof Clinical Isolates of EnterobacteriaceaeHarboring mcr-1 in Colombia, 2002 to2016 | 2002-2016 | Colombia | South America | Human | N.A | P. *Escherichia coli* | 86 | 8 | | | 8 | 2 | Chromsomal | 37 | 1 |

| | | | | | | | | | | | | | | | | | |
|---|---|---|---|---|---|---|---|---|---|---|---|---|---|---|---|---|---|
| | | Genomic and Molecular Characterizationof Clinical Isolates of EnterobacteriaceaeHarboring mcr-1 in Colombia, 2002 to2016 | 2002-2016 | Colombia | South America | Human | N.A | *Salmonella enterica* seorvar Typhimurium | 135 | 3 | | | 3 | 1 | IncFIB | 101 | 1 |
| | | Genomic and Molecular Characterizationof Clinical Isolates of EnterobacteriaceaeHarboring mcr-1 in Colombia, 2002 to2016 | 2002-2016 | Colombia | South America | Human | N.A | *Klebsiella pneumoniae* | 66 | 1 | | | 1 | | | 744 | 1 |
| | | Genomic and Molecular Characterizationof Clinical Isolates of EnterobacteriaceaeHarboring mcr-1 in Colombia, 2002 to2016 | | | | | | | | | | | | | | 1263 | 1 |
| | | Genomic and Molecular Characterizationof Clinical Isolates of EnterobacteriaceaeHarboring mcr-1 in Colombia, 2002 to2016 | | | | | | | | | | | | | | 6627 | 1 |
| | | Genomic and Molecular Characterizationof Clinical Isolates of EnterobacteriaceaeHarboring mcr-1 in Colombia, 2002 to2016 | | | | | | | | | | | | | | 3056 | 1 |
| | | Genomic and Molecular Characterizationof Clinical Isolates of EnterobacteriaceaeHarboring mcr-1 in Colombia, 2002 to2016 | | | | | | | | | | | | | | 6627 | 1 |
| **86** | 10.3389/fmicb.2017.02232 | Occurrence of Extended Spectrum β-Lactamases, KPC-Type, and MCR-1.2-Producing Enterobacteriaceae from Wells, River Water, and Wastewater Treatment Plants in Oltrepò Pavese Area, Northern Italy | 2014-2015 | Italy | Europe | Water | Water | P. *Escherichia coli* | 132 | 1 | | mcr1.2 | 1 | 1 | IncX4 | 10 | 1 |
| **87** | 10.1128/AAC.01245-17 | Heterogeneous Genetic Location of mcr-1in Colistin-Resistant Escherichia coliIsolates from Humans and Retail ChickenMeat in Switzerland: Emergence of mcr-1-Carrying IncK2 Plasmids | 2014-2016 | Switzerland | Europe | Poultry | Chicken meat | P. *Escherichia coli* | | 8 | | | 8 | 2 | IncI2 | 58 | 1 |
| | | Heterogeneous Genetic Location of mcr-1in Colistin-Resistant Escherichia coliIsolates from Humans and Retail ChickenMeat in Switzerland: Emergence of mcr-1-Carrying IncK2 Plasmids | 2014-2016 | Switzerland | Europe | Human | Fecal | P. *Escherichia coli* | | 2 | | | 2 | 2 | IncHI2 | 1775 | 1 |
| | | Heterogeneous Genetic Location of mcr-1in Colistin-Resistant Escherichia coliIsolates from Humans and Retail ChickenMeat in Switzerland: Emergence of mcr-1-Carrying IncK2 Plasmids | 2014-2016 | Switzerland | Europe | Human | Fecal | P. *Escherichia coli* | | 1 | | mcr1.2 | 1 | 2 | IncX4 | 38 | 1 |
| | | Heterogeneous Genetic Location of mcr-1in Colistin-Resistant Escherichia coliIsolates from Humans and Retail ChickenMeat in Switzerland: Emergence of mcr-1-Carrying IncK2 Plasmids | | | | | | | | | | | | 1 | IncX4 | 226 | 1 |

| | | | | | | | | | | | | | | | | | |
|---|---|---|---|---|---|---|---|---|---|---|---|---|---|---|---|---|---|
| | | Heterogeneous Genetic Location of mcr-1in Colistin-Resistant Escherichia coliIsolates from Humans and Retail ChickenMeat in Switzerland: Emergence of mcr-1-Carrying IncK2 Plasmids | | | | | | | | | | | | 2 | IncK2 | 10 | 1 |
| | | Heterogeneous Genetic Location of mcr-1in Colistin-Resistant Escherichia coliIsolates from Humans and Retail ChickenMeat in Switzerland: Emergence of mcr-1-Carrying IncK2 Plasmids | | | | | | | | | | | | 2 | Chromsomal | 5 | 1 |
| | | Heterogeneous Genetic Location of mcr-1in Colistin-Resistant Escherichia coliIsolates from Humans and Retail ChickenMeat in Switzerland: Emergence of mcr-1-Carrying IncK2 Plasmids | | | | | | | | | | | | | | 38 | 1 |
| | | Heterogeneous Genetic Location of mcr-1in Colistin-Resistant Escherichia coliIsolates from Humans and Retail ChickenMeat in Switzerland: Emergence of mcr-1-Carrying IncK2 Plasmids | | | | | | | | | | | | | | 1049 | 1 |
| | | Heterogeneous Genetic Location of mcr-1in Colistin-Resistant Escherichia coliIsolates from Humans and Retail ChickenMeat in Switzerland: Emergence of mcr-1-Carrying IncK2 Plasmids | | | | | | | | | | | | | | 5 | 1 |
| **88** | 10.1016/j.diagmicrobio.2017.06.020 | Detection and genetic features of MCR-1-producing plasmid in humanEscherichia coliinfection in South Korea | 2014-2015 | South Korea | Asia | Human | Fecal | P. *Escherichia coli* | 2082 | 1 | | | 1 | 1 | IncI2 | 167 | 1 |
| | | | | | | | | | | | | | | | | | |
| **89** | 10.3201/eid2307.161638 | Locally Acquired mcr-1 in Escherichia coli, Australia, 2011 and 2013 | 2011-2013 | Australia | Australia | Human | N.A | P. *Escherichia coli* | 4555 | 2 | | | 2 | 2 | IncI2 | 93 | 1 |
| **90** | 10.1016/j.ijantimicag.2017.05.005 | Escherichia coli ST167 carrying plasmid mobilisable mcr-1 and blaCTX-M-15 resistance determinants isolated from a human respiratory infection | 2016 | spain | Europe | Human | N.A | P. *Escherichia coli* | 48 | 1 | | | 1 | | | 167 | 1 |
| **91** | 10.1128/AAC.00317-17 | Emergence of the Plasmid-Mediated mcr-1 Gene in Clinical KPC-2-Producing Klebsiella pneumoniae Sequence Type 392 in Brazil | 2016 | Brazil | South America | Human | Urine | *Klebsiella pneumoniae* | | 1 | | | 1 | 1 | IncX4 | | |
| **92** | 10.2807/1560-7917.ES.2017.22.31.30589 | Novel plasmid-mediated colistin resistance mcr-4 gene in Salmonella and Escherichia coli, Italy 2013, Spain and Belgium, 2015 to 2016 | 2013 | Italy | Europe | Swine | Fecal | *Salmonella enterica* serovar Typhimurium | | | 1 | mcr4 | 1 | | | | |
| | | Novel plasmid-mediated colistin resistance mcr-4 gene in Salmonella | 2015-2016 | spain | Europe | Swine | Fecal | P. *Escherichia coli* | 43 | | 9 | mcr4 | 9 | | | | |

| | | | | | | | | | | | | | | | | | |
|---|---|---|---|---|---|---|---|---|---|---|---|---|---|---|---|---|---|
| | | and Escherichia coli, Italy 2013, Spain and Belgium, 2015 to 2016 | | | | | | | | | | | | | | | |
| | | Novel plasmid-mediated colistin resistance mcr-4 gene in Salmonella and Escherichia coli, Italy 2013, Spain and Belgium, 2015 to 2016 | 2015-2016 | Belgium | Europe | Swine | Fecal | P. *Escherichia coli* | 48 | | 2 | mcr4 | 2 | | | | |
| **93** | 10.1128/AAC.02632-16 | MCR-1.6, a New MCR Variant Carried by an IncP Plasmid in a Colistin-Resistant Salmonella enterica Serovar Typhimurium Isolate from a Healthy Individual | 2014 | China | Asia | Human | Fecal | *Salmonella enterica* serovar Typhimurum | 1 | 1 | | mcr1.6 | 1 | 1 | IncP | | |
| **94** | 10.1093/jac/dkw261 | Mobile MCR-1-associated resistance to colistin in Poland. | 2015 | Poland | Europe | Human | Urine | P. *Escherichia coli* | 37 | 1 | | | 1 | 1 | IncI2 | 617 | 1 |
| **95** | 10.1016/S1473-3099(15)00424-7 | Emergence of plasmid-mediated colistin resistancemechanism MCR-1 in animals and human beings in China:a microbiological and molecular biological study | 2011-2014 | China | Asia | Swine | N.A | P. *Escherichia coli* | 804 | 166 | | | 166 | | | | |
| | | Emergence of plasmid-mediated colistin resistancemechanism MCR-1 in animals and human beings in China:a microbiological and molecular biological study | 2011-2014 | China | Asia | Poultry | Chicken meat | P. *Escherichia coli* | 299 | 34 | | | 34 | | | | |
| | | Emergence of plasmid-mediated colistin resistancemechanism MCR-1 in animals and human beings in China:a microbiological and molecular biological study | 2011-2014 | China | Asia | Swine | Pork | P. *Escherichia coli* | 226 | 43 | | | 43 | | | | |
| | | Emergence of plasmid-mediated colistin resistancemechanism MCR-1 in animals and human beings in China:a microbiological and molecular biological study | 2011-2014 | China | Asia | Human | N.A | P. *Escherichia coli* | 902 | 13 | | | 13 | | | | |
| | | Emergence of plasmid-mediated colistin resistancemechanism MCR-1 in animals and human beings in China:a microbiological and molecular biological study | 2011-2014 | China | Asia | Human | N.A | *Klebsiella pneumoniae* | 420 | 3 | | | 3 | | | | |
| **96** | 10.1016/j.ijid.2017.07.003 | Emergence of colistin-resistant *Escherichia coli* clinical isolates harboring *mcr-1* in Vietnam | 2014 | Vietnam | Asia | Human | N.A | P. *Escherichia coli* | 18 | 2 | | | 2 | 2 | Chromsomal | 410 | 1 |
| | | Emergence of colistin-resistant *Escherichia coli* clinical isolates harboring *mcr-1* in Vietnam | | | | | | | | | | | | | | 457 | 1 |
| **97** | 10.1016/j.ijid.2017.07.023 | Emergence of a colistin-resistant *Escherichia coli* clinical isolate harboring *mcr-1* in Japan | 2017 | Japan | Asia | Human | Fecal | P. *Escherichia coli* | 4 | 1 | | | 1 | 1 | IncHI2 | 5702 | 1 |
| **98** | 10.3201/eid2207.160234 | **Colistin-Resistant *mcr-1*–Positive Pathogenic *Escherichia coli* in Swine, Japan, 2007–2014** | 2007-2014 | Japan | Asia | Human | Fecal | P. *Escherichia coli* | 684 | 90 | | | 90 | | | | |
| **99** | 10.1093/jac/dkx078 | ***Citrobacter braakii* carrying plasmid-borne *mcr-1* colistin resistance gene** | 2013 | Bolivia | South America | Ready-to-eat food | N.A | Citrobacter braakii | 83 | 1 | | | 1 | | | | |

| | | | | | | | | | | | | | | | | | |
|---|---|---|---|---|---|---|---|---|---|---|---|---|---|---|---|---|---|
| | | **from ready-to-eat food from a market in the Chaco region of Bolivia** | | | | | | | | | | | | | | | |
| **100** | 10.1016/j.jinf.2017.09.008 | **Prevalence of colistin resistance in clinical isolates of Enterobacteriaceae: A four-year cross-sectional study** | 2012-2015 | Spain | Europe | Human | N.A | P. *Escherichia coli* | 53 | 1 | | | 1 | | | | |
| **101** | 10.2807/1560-7917.ES.2017.22.19.30530 | Increasing proportion of carbapenemase-producing Enterobacteriaceae and emergence of a MCR-1 producer through a multicentric study among hospital-based and private laboratories in Belgium from September to November 2015. | 2015 | Belgium | Europe | Human | N.A | P. *Escherichia coli* | 3836 | 1 | | | 1 | | | | |
| **102** | 10.1089/mdr.2017.0131 | **Plasmid-Mediated Colistin Resistance Gene mcr-1 in an Escherichia coli ST10 Bloodstream Isolate in the Sultanate of Oman** | **2014-2016** | Oman | Asia | Human | Blood | P. *Escherichia coli* | 22 | 1 | | | 1 | | | | |
| **103** | 10.1016/j.jgar.2017.08.002 | Colistin resistance among blood culture isolates at a tertiary care centre in Hungary. | 2010-2011 | Hungary | Europe | Human | Blood | P. *Escherichia coli* | 146 | 1 | | | 1 | 1 | IncI2 | 10 | 1 |
| **104** | 10.1186/s13756-017-0234-8 | Plasmid-mediated mcr-1 colistin resistanceinEscherichia coliandKlebsiella spp.clinicalisolates from the Western Cape region of South Africa | 2016 | South Africa | Africa | Human | Urine | P. *Escherichia coli* | | 10 | | | 10 | | | | |
| **105** | 10.2807/1560-7917.ES.2016.21.27.30280 | Identification of a novel plasmid-mediated colistin-resistance gene, mcr-2, in Escherichia coli, Belgium, June 2016. | 2016 | Belgium | Europe | Swine | Fecal | P. *Escherichia coli* | 10 | | | | | | | | |
| | | Identification of a novel plasmid-mediated colistin-resistance gene, mcr-2, in Escherichia coli, Belgium, June 2016. | 2016 | Belgium | Europe | Swine | N.A | P. *Escherichia coli* | | | 2 | mcr2 | 2 | 2 | IncX4 | | |
| | | Identification of a novel plasmid-mediated colistin-resistance gene, mcr-2, in Escherichia coli, Belgium, June 2016. | 2016 | Belgium | Europe | Bovine | N.A | P. *Escherichia coli* | | | 1 | mcr2 | 1 | 1 | IncX4 | | |
| **106** | 10.1089/fpd.2017.2329 | Detection of Plasmid-Mediated Colistin Resistance, *mcr-1* and *mcr-2* Genes, in *Salmonella* spp. Isolated from Food at Retail in Belgium from 2012 to 2015 | 2012-2015 | Belgium | Europe | Mixed food sources | N.A | *Salmonella enterica* serovar Typhimurim | 105 | | | | | | | | |
| | | Detection of Plasmid-Mediated Colistin Resistance, *mcr-1* and *mcr-2* Genes, in *Salmonella* spp. Isolated from Food at Retail in Belgium from 2012 to 2015 | 2012-2015 | Belgium | Europe | Swine | Pork | *Salmonella enterica* serovar Typhimurim | | 1 | | | 1 | 1 | IncX4 | | |
| | | Detection of Plasmid-Mediated Colistin Resistance, *mcr-1* and *mcr-2* Genes, in *Salmonella* spp. Isolated from Food at Retail in Belgium from 2012 to 2015 | 2012-2015 | Belgium | Europe | Swine | Pork | *Salmonella enterica* serovar Derby | | | 1 | mcr2 | 1 | 1 | IncX4 | | |

| | | | | | | | | | | | | | | | | | |
|---|---|---|---|---|---|---|---|---|---|---|---|---|---|---|---|---|---|
| | | Detection of Plasmid-Mediated Colistin Resistance, *mcr-1* and *mcr-2* Genes, in *Salmonella* spp. Isolated from Food at Retail in Belgium from 2012 to 2015 | 2012-2015 | Belgium | Europe | N.A | N.A | *Salmonella enterica* serovar Typhimurim | | 1 | | | 1 | 1 | IncX4 | | |
| **107** | 10.3201/eid2209.160091 | Colistin-Resistant Enterobacteriaceae Carryingthe mcr-1 Gene among Patients in Hong Kong | 2015-2016 | China | Asia | Human | N.A | P. *Escherichia coli* | 1324 | 4 | | | 4 | 4 | IncI2 | | |
| | | Colistin-Resistant Enterobacteriaceae Carryingthe mcr-1 Gene among Patients in Hong Kong | 2015-2016 | China | Asia | Human | N.A | *Acintobacter* | 103 | 1 | | | 1 | 1 | IncI2 | | |
| **108** | 10.2807/1560-7917.ES.2017.22.39.17-00206 | Widespread distribution of mcr-1-bearing bacteria in the ecosystem, 2015 to 2016 | 2015-2016 | China | Asia | Human | N.A | P. *Escherichia coli* | | 84 | | | 84 | | | | |
| | | Widespread distribution of mcr-1-bearing bacteria in the ecosystem, 2015 to 2016 | 2015-2016 | China | Asia | Human | N.A | *Enterobacter cloacae* | | 4 | | | 4 | | | | |
| | | Widespread distribution of mcr-1-bearing bacteria in the ecosystem, 2015 to 2016 | 2015-2016 | China | Asia | Water | Water | P. *Escherichia coli* | | 77 | | | 77 | | | | |
| | | Widespread distribution of mcr-1-bearing bacteria in the ecosystem, 2015 to 2016 | 2015-2016 | China | Asia | Water | Water | *Klebsiella pneumoniae* | | 4 | | | 4 | | | | |
| | | Widespread distribution of mcr-1-bearing bacteria in the ecosystem, 2015 to 2016 | 2015-2016 | China | Asia | Water | Water | *Klebsiella variicola* | | 3 | | | 3 | | | | |
| **109** | 10.1128/AAC.01139-17 | Emergence of mcr-1 in Raoultella ornithinolytica and Escherichia coli Isolates from Retail Vegetables in China | 2015-2016 | China | Asia | Vegetables | N.A | P. *Escherichia coli* | | 4 | | | 4 | | | | |
| | | Emergence of mcr-1 in Raoultella ornithinolytica and Escherichia coli Isolates from Retail Vegetables in China | 2015-2016 | China | Asia | Vegetables | N.A | *Raoultella ornithinolytica* | | 2 | | | 2 | | | | |
| **110** | 10.1093/jac/dkw245 | Detection of the mcr-1 gene in Escherichia coli prevalent in the migratory bird species Larus argentatus. | 2016 | lithuania | Asia | Poultry | N.A | P. *Escherichia coli* | 117 | 1 | | | 1 | | | | |
| **111** | 10.1093/jac/dkw262 | The colistin resistance *mcr*-1 gene is going wild | 2012 | Argentina | South America | Poultry | N.A | P. *Escherichia coli* | | 5 | | | 5 | | | | |
| **112** | thesis:YZU2017YananTian | The analysis of antimicrobial resistance and the plasmid mediated resistant genes in Salmonella Typhimurium isolates | 2015 | China | Asia | Human | Fecal | *Salmonella enterica* serovar Typhimurium | 20 | 2 | | | 2 | | | | |
| | | The analysis of antimicrobial resistance and the plasmid mediated resistant genes in Salmonella Typhimurium isolates | 2012-2015 | China | Asia | Poultry | Saliva, Urine | *Salmonella enterica* serovar Typhimurium | 66 | 15 | | | 15 | | | | |
| **113** | 10.3760/cma.j.issn.1674-2397.2016.05.013 | Analysis of carbapenemases genes and colistin resistance gene mcr-1 in carbapenem-resistant Enterobacteriaceae | 2014-2015 | China | Asia | Human | N.A | P. *Escherichia coli* | 34 | 1 | | | 1 | 1 | IncX4 | | |

| | | | | | | | | | | | | | | | | | |
|---|---|---|---|---|---|---|---|---|---|---|---|---|---|---|---|---|---|
| | | Analysis of carbapenemases genes andcolistin resistance gene mcr-1 in carbapenem-resistant Enterobacteriaceae | 2014-2015 | China | Asia | Human | N.A | *Klebsiella pneumoniae* | 34 | 1 | | | 1 | 1 | IncX4 | | |
| **114** | 10.3760/cma.j.issn.1009-9158.2016.08.014 | Molecular mechanisms of colistin - resistant Klebsiella pneumoniae and Escherichia coli isolates | 2011-2014 | China | Asia | Human | Blood, secreta, phlegm, urine | *Klebsiella pneumoniae* | 964 | 4 | | | 4 | | | | |
| | | Molecular mechanisms of colistin - resistant Klebsiella pneumoniae and Escherichia coli isolates | 2011-2014 | China | Asia | Human | N.A | P. *Escherichia coli* | 1389 | 23 | | | 23 | | | | |
| **115** | thesis:SCAU2016YiyunLiu | Emergence of Plasmid-Mediated Colistin Resistance Gene mcr-1 in Enterobacteriacea | 2012-2014 | China | Asia | Swine | Fecal | P. *Escherichia coli* | 804 | 66 | | | 66 | | | | |
| **116** | thesis:SCAU2016DanYao | Molecular Epidemiology of ESBLs- and Carbapenemases-producing Enterobacteriaceae from Retail Meat and Vegetables | 2014-2015 | China | Asia | Lettuce | N.A | P. *Escherichia coli* | 1 | 1 | | | 1 | | | 877 | 1 |
| **117** | thesis:SCAU2016LinfengYu | Transfer Mechanism of Colistin Resistance Gene mcr-1 among Escherichia coli Isolates from a Livestock Farm of Shanghai | 2013 | China | Asia | Swine | Fecal | P. *Escherichia coli* | 47 | 27 | | | 27 | | | | |
| **118** | thesis:JZMU2017JiananSun | Study on the Drug resistance of Streptococcus in the Main Links of Pig Industry Chain in Western Liaoning province and It strians mission | 2015-2017 | China | Asia | Swine | N.A | | 142 | 6 | | | 6 | | | | |
| | | Study on the Drug resistance of Streptococcus in the Main Links of Pig Industry Chain in Western Liaoning province and It strians mission | 2015-2017 | China | Asia | Swine | Pork | | 22 | 5 | | | 5 | | | | |
| | | Study on the Drug resistance of Streptococcus in the Main Links of Pig Industry Chain in Western Liaoning province and It strians mission | 2015-2017 | China | Asia | Human | N.A | | 8 | 4 | | | 4 | | | | |
| **119** | thesis:CAU2017RongmingZhang | The molecular epidemiology of The NDM and MCR-l positive E. Coli from poultry production chain | 2014 | China | Asia | Poultry | Fecal | P. *Escherichia coli* | 407 | 131 | | | 131 | 6 | Chromsomal | 10 | 46 |
| | | The molecular epidemiology of The NDM and MCR-l positive E. Coli from poultry production chain | 2014-2015 | China | Asia | Poultry | Chicken meat | P. *Escherichia coli* | 42 | 12 | | | 12 | | | 156 | 1 |
| | | The molecular epidemiology of The NDM and MCR-l positive E. Coli from poultry production chain | 2014 | China | Asia | Dog | N.A | P. *Escherichia coli* | 17 | 5 | | | 5 | | | 48 | 1 |
| | | The molecular epidemiology of The NDM and MCR-l positive E. Coli from poultry production chain | 2014 | China | Asia | Water | Water | P. *Escherichia coli* | 5 | 2 | | | 2 | | | 162 | 1 |
| | | The molecular epidemiology of The NDM and MCR-l positive E. Coli from poultry production chain | 2014 | China | Asia | Flies | N.A | P. *Escherichia coli* | 93 | 16 | | | 16 | | | 167 | 1 |
| | | The molecular epidemiology of The NDM and MCR-l positive E. Coli from poultry production chain | | | | | | | | | | | | | | 175 | 1 |

| | | | | | | | | | | | | | | | | | |
|---|---|---|---|---|---|---|---|---|---|---|---|---|---|---|---|---|---|
| | | The molecular epidemiology of The NDM and MCR-l positive E. Coli from poultry production chain | | | | | | | | | | | | | | 206 | 1 |
| | | The molecular epidemiology of The NDM and MCR-l positive E. Coli from poultry production chain | | | | | | | | | | | | | | 114 | 1 |
| | | The molecular epidemiology of The NDM and MCR-l positive E. Coli from poultry production chain | | | | | | | | | | | | | | 354 | 1 |
| | | The molecular epidemiology of The NDM and MCR-l positive E. Coli from poultry production chain | | | | | | | | | | | | | | 359 | 1 |
| | | The molecular epidemiology of The NDM and MCR-l positive E. Coli from poultry production chain | | | | | | | | | | | | | | 602 | 1 |
| **120** | thesis:CAU2017 MingquanChui | Study on the transmission mechanism of mcr-1 gene in Salmonella and the microarray assay for the detection of foodborne pathogens, their resistance and virulence genes | 2012-2014 | China | Asia | Human | Fecal | *Salmonella enterica* serovar Typhimurium | 2034 | 28 | | | 28 | 1 | IncX4 | | |
| | | Study on the transmission mechanism of mcr-1 gene in Salmonella and the microarray assay for the detection of foodborne pathogens, their resistance and virulence genes | 2012-2014 | China | Asia | Human | Fecal | *Salmonella enterica* serovar Typhimurium | | | | | | 1 | IncI2 | | |
| | | Study on the transmission mechanism of mcr-1 gene in Salmonella and the microarray assay for the detection of foodborne pathogens, their resistance and virulence genes | 2012-2014 | China | Asia | Human | Fecal | *Salmonella enterica* serovar Typhimurium | | | | | | 2 | IncHI2 | | |
| | | Study on the transmission mechanism of mcr-1 gene in Salmonella and the microarray assay for the detection of foodborne pathogens, their resistance and virulence genes | 2012-2014 | China | Asia | Swine | Fecal | *Salmonella enterica* serovar Typhimurium | 743 | 26 | | | 26 | 5 | IncHI2 | | |
| **121** | thesis:JLAU2017YixiaoHan | Virulence and resistance analysis of the Echerichia coli isolated from diarrhea piglets | 2015-2016 | China | Asia | Swine | Fecal | P. *Escherichia coli* | 102 | 11 | | | 11 | | | | |
| | | Virulence and resistance analysis of the Echerichia coli isolated from diarrhea piglets | | | | | | P. *Escherichia coli* | | | | | | | | | |
| **122** | 10.1016/j.ebiom.2018.07.027 | Spread of MCR-3 Colistin Resistance in China: An Epidemiological, Genomic and Mechanistic Study | 2016 | China | Asia | Human | Fecal | P. *Escherichia coli* | 9 | | 9 | mcr3 | 9 | 3 | IncP | | |
| | | Spread of MCR-3 Colistin Resistance in China: An Epidemiological, Genomic and Mechanistic Study | 2017 | China | Asia | Swine | Fecal | P. *Escherichia coli* | 40 | | 40 | mcr3 | 40 | 1 | IncHI2 | | |
| **123** | 10.1038/s41426-018-0033-1 | Rapid rise of the ESBL and mcr-1 genes in Escherichia coli of chicken origin in China, 2008–2014 | 2008-2014 | China | Asia | Poultry | N.A | P. *Escherichia coli* | 341 | 44 | | | 44 | 28 | IncI2 | 10 | 3 |
| | | Rapid rise of the ESBL and mcr-1 genes in Escherichia coli of chicken origin in China, 2008–2014 | | | | | | | | | | | | | | 29 | 3 |

| | | | | | | | | | | | | | | | | | |
|---|---|---|---|---|---|---|---|---|---|---|---|---|---|---|---|---|---|
| | | Rapid rise of the ESBL and mcr-1 genes in Escherichia coli of chicken origin in China, 2008–2014 | | | | | | | | | | | | | | 101 | 3 |
| | | Rapid rise of the ESBL and mcr-1 genes in Escherichia coli of chicken origin in China, 2008–2014 | | | | | | | | | | | | | | 354 | 3 |
| | | Rapid rise of the ESBL and mcr-1 genes in Escherichia coli of chicken origin in China, 2008–2014 | | | | | | | | | | | | | | 1268 | 4 |
| | | Rapid rise of the ESBL and mcr-1 genes in Escherichia coli of chicken origin in China, 2008–2014 | | | | | | | | | | | | | | 46 | 9 |
| **124** | 10.1093/cid/ciy683 | Transmission of Mobile Colistin Resistance (mcr-1) by Duodenoscope | 2017 | United States | North America | Human | Fecal | *Klebsiella pneumoniae* | 14 | 2 | | | 2 | | | | |
| **125** | 10.1080/21505594.2018.1462060 | Discovery of a mcr-1-bearing plasmid in commensal colistin-resistant Escherichia coli from healthy broilers in Faisalabad, Pakistan | 2016-2017 | Pakistan | Asia | Poultry | Fecal | Non P. Escherichia coli | | 8 | | | 8 | 8 | IncI2 | 10 | 1 |
| | | Discovery of a mcr-1-bearing plasmid in commensal colistin-resistant Escherichia coli from healthy broilers in Faisalabad, Pakistan | | | | | | | | | | | | | | 155 | 1 |
| | | Discovery of a mcr-1-bearing plasmid in commensal colistin-resistant Escherichia coli from healthy broilers in Faisalabad, Pakistan | | | | | | | | | | | | | | 2847 | 1 |
| | | Discovery of a mcr-1-bearing plasmid in commensal colistin-resistant Escherichia coli from healthy broilers in Faisalabad, Pakistan | | | | | | | | | | | | | | 6395 | 3 |
| | | Discovery of a mcr-1-bearing plasmid in commensal colistin-resistant Escherichia coli from healthy broilers in Faisalabad, Pakistan | | | | | | | | | | | | | | 361 | 4 |
| **126** | 10.1016/j.ijantimicag.2018.02.019 | Multidrug-resistant Shigella sonnei carrying the plasmid-me diate d mcr - 1 gene in China | 2010-2012 | China | Asia | Human | Fecal | *Shigella sonnei* | 1650 | 6 | | | 6 | 6 | IncI2 | | |
| **127** | 10.1128/JCM.01562-17 | mcr-3 and mcr-4 Variants in Carbapenemase-Producing Clinical Enterobacteriaceae Do Not Confer Phenotypic PolymyxinResistance | | Singapore | Asia | Human | N.A | P. *Escherichia coli* | 500 | | 1 | mcr3 | 1 | | | | |
| | | mcr-3 and mcr-4 Variants in Carbapenemase-Producing Clinical Enterobacteriaceae Do Not Confer Phenotypic PolymyxinResistance | | Singapore | Asia | Human | N.A | P. *Escherichia coli* | 500 | | 1 | mcr4.2 | 1 | | | | |
| **128** | 10.3760/cma.j.issn.0254-5101.2018.01.007 | Detection and epidemiology of mcr-1-positive Escherichia coli strains in Dongyang area of Zhejiang Province | 2016 | China | Asia | Human | N.A | P. *Escherichia coli* | 315 | 5 | | | 5 | | | 43 | 2 |
| | | Detection and epidemiology of mcr-1-positive Escherichia coli strains in Dongyang area of Zhejiang Province | | | | | | | | | | | | | | 69 | 1 |

| | | | | | | | | | | | | | | | | | |
|---|---|---|---|---|---|---|---|---|---|---|---|---|---|---|---|---|---|
| | | Detection and epidemiology of mcr-1-positive Escherichia coli strains in Dongyang area of Zhejiang Province | | | | | | | | | | | | | | 131 | 1 |
| | | Detection and epidemiology of mcr-1-positive Escherichia coli strains in Dongyang area of Zhejiang Province | | | | | | | | | | | | | | 349 | 1 |
| | | Detection and epidemiology of mcr-1-positive Escherichia coli strains in Dongyang area of Zhejiang Province | | | | | | | | | | | | | | | |
| **129** | 10.1093/cid/cix885 | High Rates of Human Fecal Carriage of mcr-1-Positive Multidrug-Resistant Enterobacteriaceae Emerge in China in Association With Successful Plasmid Families | 2011-2016 | China | Asia | Human | Fecal | P. *Escherichia coli* | 497 | 321 | | | 321 | 27 | IncI2 | 156 | 1 |
| | | High Rates of Human Fecal Carriage of mcr-1-Positive Multidrug-Resistant Enterobacteriaceae Emerge in China in Association With Successful Plasmid Families | 2011-2016 | China | Asia | Human | Fecal | P. *Escherichia coli* | | 27 | | | 27 | 15 | IncX4 | 131 | 1 |
| | | High Rates of Human Fecal Carriage of mcr-1-Positive Multidrug-Resistant Enterobacteriaceae Emerge in China in Association With Successful Plasmid Families | 2011-2016 | China | Asia | Human | Fecal | P. *Escherichia coli* | | 15 | | | 15 | 22 | IncHI2 | 155 | 1 |
| | | High Rates of Human Fecal Carriage of mcr-1-Positive Multidrug-Resistant Enterobacteriaceae Emerge in China in Association With Successful Plasmid Families | 2011-2016 | China | Asia | Human | Fecal | P. *Escherichia coli* | | 22 | | | 22 | | | 405 | 1 |
| | | High Rates of Human Fecal Carriage of mcr-1-Positive Multidrug-Resistant Enterobacteriaceae Emerge in China in Association With Successful Plasmid Families | 2011-2016 | China | Asia | Human | Fecal | *Klebsiella pneumoniae* | | 13 | | | 13 | | | | |
| | | High Rates of Human Fecal Carriage of mcr-1-Positive Multidrug-Resistant Enterobacteriaceae Emerge in China in Association With Successful Plasmid Families | 2011-2016 | China | Asia | Human | Fecal | *Enterobacter cloacae* | | 1 | | | 1 | | | | |
| **130** | 10.1093/jac/dky111 | Novel plasmid-mediated colistin resistance gene mcr-7.1 in Klebsiella pneumoniae | 2015 | China | Asia | Poultry | N.A | *Klebsiella pneumoniae* | 183 | 7 | | | 7 | | | | |
| | | Novel plasmid-mediated colistin resistance gene mcr-7.1 in Klebsiella pneumoniae | 2015 | China | Asia | Poultry | N.A | *Klebsiella pneumoniae* | | | 1 | mcr7.1 | 1 | 8 | IncI2 | | |
| **131** | 10.1093/femsle/fny100 | The presence of colistin resistance gene mcr-1 and -3 in ESBL producing Escherichia coli isolated from food in Ho Chi Minh City, Vietnam | 2012-2014 | Vietnam | Asia | N.A | N.A | Non P. Escherichia coli | 261 | | | | | | | | |
| | | The presence of colistin resistance gene mcr-1 and -3 in ESBL producing Escherichia coli isolated from food in Ho Chi Minh City, Vietnam | 2012-2014 | Vietnam | Asia | N.A | N.A | Non P. Escherichia coli | | 60 | | | 60 | | | | |

| | | | | | | | | | | | | | | | | | |
|---|---|---|---|---|---|---|---|---|---|---|---|---|---|---|---|---|---|
| | | The presence of colistin resistance gene mcr-1 and -3 in ESBL producing Escherichia coli isolated from food in Ho Chi Minh City, Vietnam | 2012-2014 | Vietnam | Asia | N.A | N.A | Non P. Escherichia coli | | | 2 | mcr3 | 2 | | | | |
| **132** | 10.3389/fmicb.2018.00745 | Evidence for Environmental Dissemination of Antibiotic Resistance Mediated by Wild Birds | 2015 | China | Asia | Water | Water | Non P. Escherichia coli | 5 | 3 | | | 3 | | | | |
| | | Evidence for Environmental Dissemination of Antibiotic Resistance Mediated by Wild Birds | 2015 | China | Asia | Poultry | Fecal | Non P. Escherichia coli | 5 | 1 | | | 1 | | | | |
| **133** | 10.1016/j.vetmic.2018.04.002 | Emergence of the colistin resistance gene mcr-1 and its variant in several uncommon species of Enterobacteriaceae from commercial poultry farm surrounding environments | 2016 | China | Asia | Poultry | Fecal | Non P. Escherichia coli | 22 | 2 | | | 2 | 2 | IncI2 | | |
| | | Emergence of the colistin resistance gene mcr-1 and its variant in several uncommon species of Enterobacteriaceae from commercial poultry farm surrounding environments | 2016 | China | Asia | Poultry | Fecal | Non P. Escherichia coli | | 20 | | | 20 | | | | |
| | | Emergence of the colistin resistance gene mcr-1 and its variant in several uncommon species of Enterobacteriaceae from commercial poultry farm surrounding environments | 2016 | China | Asia | Poultry | Fecal | *Pseudomonas alcalifaciens* | | 1 | | | 1 | | | | |
| | | Emergence of the colistin resistance gene mcr-1 and its variant in several uncommon species of Enterobacteriaceae from commercial poultry farm surrounding environments | 2016 | China | Asia | Poultry | Fecal | *Raoultella planticola* | | 1 | | mcr1.3 | 1 | 1 | IncI2 | | |
| | | Emergence of the colistin resistance gene mcr-1 and its variant in several uncommon species of Enterobacteriaceae from commercial poultry farm surrounding environments | 2016 | China | Asia | Poultry | Fecal | *Enterobacter cloacae* | | 1 | | | 1 | 1 | IncX4 | | |
| | | Emergence of the colistin resistance gene mcr-1 and its variant in several uncommon species of Enterobacteriaceae from commercial poultry farm surrounding environments | 2016 | China | Asia | Poultry | Fecal | *Salmonella enterica* | | 5 | | | 5 | | | | |
| **134** | 10.1016/j.ijid.2018.08.011 | Emergence of IncX4 plasmids encoding mcr-1 in a clinical isolate of Klebsiella pneumoniae in Japan | 2017 | Japan | Asia | Human | Fecal | *Klebsiella pneumoniae* | 5 | 4 | | | 4 | 2 | IncX4 | | |
| | | Emergence of IncX4 plasmids encoding mcr-1 in a clinical isolate of Klebsiella pneumoniae in Japan | | | | | | | | | | | | 2 | IncI2 | | |

| | | | | | | | | | | | | | | | | | |
|---|---|---|---|---|---|---|---|---|---|---|---|---|---|---|---|---|---|
| | | Emergence of IncX4 plasmids encoding mcr-1 in a clinical isolate of Klebsiella pneumoniae in Japan | 2017 | Japan | Asia | Human | Fecal | *Klebsiella pneumoniae* | | 1 | | | 1 | 1 | IncX4 | | |
| **135** | 10.3389/fmed. 2018.00099 | alkaline Peptone Water-Based enrichment Method for mcr-3 From acute Diarrheic Outpatient gut samples | 2017 | China | Asia | Human | Fecal | P. *Escherichia coli* | | 29 | | | 29 | | | | |
| | | alkaline Peptone Water-Based enrichment Method for mcr-3 From acute Diarrheic Outpatient gut samples | 2017 | China | Asia | Human | Fecal | *Aeromonas veronii* | | | 8 | mcr3 | 8 | | | | |
| **136** | 10.1089/mdr.2 017.0400 | Genomic Characterization of Nonclonal *mcr*-1-Positive Multidrug-Resistant *Klebsiella pneumoniae* from Clinical Samples in Thailand | 2017 | Thailand | Asia | Human | N.A | *Klebsiella pneumoniae* | | 2 | | | 2 | 2 | IncX4 | | |
| **137** | 10.1128/AAC.0 1885-17 | Emergence of Chromosome-Borne Colistin Resistance Gene mcr-1 in Clinical Isolates of Klebsiella pneumoniae from India | 2016 | India | Asia | Human | N.A | *Klebsiella pneumoniae* | | 4 | | | 4 | | | | |
| | | Emergence of Chromosome-Borne Colistin Resistance Gene mcr-1 in Clinical Isolates of Klebsiella pneumoniae from India | | China | Asia | Swine | Pork | *Aeromonas* | | | 1 | mcr3 | 1 | | | | |
| | | Emergence of Chromosome-Borne Colistin Resistance Gene mcr-1 in Clinical Isolates of Klebsiella pneumoniae from India | | China | Asia | Poultry | Chicken meat | *Aeromonas* | | | 3 | mcr3 | 3 | | | | |
| | | Emergence of Chromosome-Borne Colistin Resistance Gene mcr-1 in Clinical Isolates of Klebsiella pneumoniae from India | | China | Asia | Aquatic environm ent | N.A | *Aeromonas* | | | 4 | mcr3 | 4 | | | | |
| **138** | 10.1111/jam.13 907 | Longitudinal study on the occurrence in pigs of colistin-resistant Escherichia coli carrying mcr-1 following the cessation of use of colistin | 2015 | United Kingdom | Europe | Swine | Fecal | P. *Escherichia coli* | 9 | 8 | | | 8 | | | | |
| | | Longitudinal study on the occurrence in pigs of colistin-resistant Escherichia coli carrying mcr-1 following the cessation of use of colistin | 2015 | United Kingdom | Europe | Swine | Fecal | *Salmonella enterica* | | 1 | | | 1 | | | | |
| **139** | 10.2147/IDR.S1 60489 | Multicenter prospective study on the prevalence of colistin resistance in Escherichia coli: relevance of mcr-1-positive clinical isolates in lombardy, northern Italy | 2016 | italy | Europe | Human | N.A | P. *Escherichia coli* | 18 | 10 | | | 10 | | | | |
| **140** | 10.1128/AAC.0 1021-18 | Screening and Characterization of Multidrug-Resistant GramNegative Bacteria from a Remote African Area, São Tomé and Príncipe | 2016-2017 | Portugal | Europe | Human | N.A | P. *Escherichia coli* | 30 | 1 | | | 1 | | | | |
| **141** | 10.3201/eid240 4.171787 | mcr-1 in Carbapenemase-Producing Klebsiella pneumoniae in Hospitalized Patients, Portugal, 2016–2017 | 2016-2017 | Portugal | Europe | Human | N.A | *Klebsiella pneumoniae* | 359 | 24 | | | 24 | 24 | IncX4 | | |

| | | | | | | | | | | | | | | | | | |
|---|---|---|---|---|---|---|---|---|---|---|---|---|---|---|---|---|---|
| **142** | 10.1016/j.ijantimicag.2018.08.008 | Mobile Colistin Resistance Genes in Escherichia coli from Pigs Affected by Colibacillosis | 2015-2016 | Italy | Europe | Swine | N.A | P. *Escherichia coli* | 33 | 17 | | | 17 | | | | |
| | | Mobile Colistin Resistance Genes in Escherichia coli from Pigs Affected by Colibacillosis | 2015-2016 | Italy | Europe | Swine | N.A | P. *Escherichia coli* | | | 3 | mcr2 | 3 | | | | |
| | | Mobile Colistin Resistance Genes in Escherichia coli from Pigs Affected by Colibacillosis | 2015-2016 | Italy | Europe | Swine | N.A | P. *Escherichia coli* | | | 10 | mcr4 | 10 | | | | |
| **143** | 10.1016/j.ijfoodmicro.2018.01.017 | Emergence of plasmid-mediated colistin-resistance in CMY-2-producing Escherichia coli of lineage ST2197 in a Tunisian poultry farm | 2013 | Tunisia | Africa | Poultry | Fecal | P. *Escherichia coli* | 48 | 2 | | | 2 | 2 | IncF | 2197 | 2 |
| **144** | 10.1093/jac/dky110 | Mobile colistin resistance gene mcr-5 in porcine Aeromonas hydrophila | 2017 | China | Asia | Swine | Fecal | *Aeromonas hydrophila* | 8 | | 1 | mcr5 | 1 | 1 | IncFIB | | |
| **145** | 10.1128/AAC.02378-17 | Detection of mcr-1 Gene among Escherichia coli Isolates from Farmed Fish and Characterization of mcr-1-Bearing IncP Plasmids | 2016-2017 | China | Asia | Fish | N.A | Non P. Escherichia coli | 192 | 7 | | | 7 | 2 | IncI2 | 48 | 1 |
| | | Detection of mcr-1 Gene among Escherichia coli Isolates from Farmed Fish and Characterization of mcr-1-Bearing IncP Plasmids | | | | | | | | | | | | | | 4014 | 1 |
| | | Detection of mcr-1 Gene among Escherichia coli Isolates from Farmed Fish and Characterization of mcr-1-Bearing IncP Plasmids | | | | | | | | | | | | 2 | Inck | 7508 | 1 |
| | | Detection of mcr-1 Gene among Escherichia coli Isolates from Farmed Fish and Characterization of mcr-1-Bearing IncP Plasmids | | | | | | | | | | | | 1 | IncX4 | 101 | 1 |
| | | Detection of mcr-1 Gene among Escherichia coli Isolates from Farmed Fish and Characterization of mcr-1-Bearing IncP Plasmids | | | | | | | | | | | | 2 | Incx | 2040 | 1 |
| | | Detection of mcr-1 Gene among Escherichia coli Isolates from Farmed Fish and Characterization of mcr-1-Bearing IncP Plasmids | | | | | | | | | | | | | | 7030 | 1 |
| | | Detection of mcr-1 Gene among Escherichia coli Isolates from Farmed Fish and Characterization of mcr-1-Bearing IncP Plasmids | | | | | | | | | | | | | | 156 | 1 |
| **146** | 10.1093/jac/dkx489 | Emergence of blaCTX-M-55 associated with fosA, rmtB and mcr gene variants in Escherichia coli from various animal species in France | 2010-2013 | France | Europe | N.A | N.A | P. *Escherichia coli* | 21 | 1 | | | 10 | 1 | IncP | 2930 | 1 |
| | | | 2010-2013 | France | Europe | N.A | N.A | P. *Escherichia coli* | | | 10 | mcr3 | 1 | 1 | IncP | 540 | 1 |
| **147** | 10.1128/JCM.01932-17 | Rapid Increase in Prevalence of Carbapenem-Resistant Enterobacteriaceae (CRE) and Emergence of Colistin Resistance | 2014-2016 | China | Asia | Human | N.A | P. *Escherichia coli* | 1311 | 1 | | | 1 | | | | |

| | | | | | | | | | | | | | | | | |
|---|---|---|---|---|---|---|---|---|---|---|---|---|---|---|---|---|
| | | Gene mcr-1 in CRE in a Hospital in Henan, China | | | | | | | | | | | | | | |
| **148** | 10.1093/jac/dky038 | Occurrence of the mobile colistin resistance gene mcr-3 in Escherichia coli from household pigs in rural areas | 2015 | China | Asia | Swine | Fecal | Non P. Escherichia coli | | 71 | | | 71 | 2 | IncP | | |
| | | Occurrence of the mobile colistin resistance gene mcr-3 in Escherichia coli from household pigs in rural areas | 2015 | China | Asia | Swine | Fecal | Non P. Escherichia coli | | | 5 | mcr3 | 5 | 2 | IncHI2 | | |
| **149** | 10.1128/AAC.01304-18 | The Colistin Resistance Gene, mcr-1, is Prevalent in Commensal E. coli Isolated from Lebanese Pre-harvest Poultry in Lebanon | 2017-2018 | Lebanon | Asia | Poultry | Fecal | Non P. Escherichia coli | 90 | 88 | | | 88 | | | | |
| **150** | 10.1016/j.ijantimicag.2017.08.007 | Investigation of potential risk factors for the occurrence of Escherichia coli isolates from German fattening pig farms harbouring the mcr-1 colistin–resistance gene | 2011-2012 | Germany | Europe | Swine | Fecal | Non P. Escherichia coli | 216 | 26 | | | 26 | | | | |
| **151** | 10.1111/apm.12834 | The first human report of mobile colistin resistance gene, mcr-1, in Finland | 2016 | Finland | Europe | Human | Fecal | P. *Escherichia coli* | | 1 | | | 1 | 1 | IncX4 | 93 | 1 |
| **152** | 10.1016/j.ijantimicag.2018.03.022 | Co-occurrence of mcr-1, mcr-4 and mcr-5 genes in multidrug-resistant ST10 Enterotoxigenic and Shiga toxin-producing Escherichia coli in Spain (2006-2017) | 2006-2017 | spain | Europe | Swine | N.A | P. *Escherichia coli* | 186 | 37 | 102 | mcr4 | 139 | | | | |
| | | Co-occurrence of mcr-1, mcr-4 and mcr-5 genes in multidrug-resistant ST10 Enterotoxigenic and Shiga toxin-producing Escherichia coli in Spain (2006-2017) | 2006-2017 | spain | Europe | Swine | N.A | P. *Escherichia coli* | | | 5 | mcr5 | 5 | | | | |
| **153** | 10.2147/IDR.S171164 | Co-production of mCR-1 and nDm-5 in Escherichia coli isolated from a colonization case of inpatient | 2016 | China | Asia | Human | N.A | P. *Escherichia coli* | 88 | 1 | | | 1 | 1 | IncX4 | 46 | 1 |
| **154** | 10.1093/jac/dkx538 | Identification of novel variants of the colistin resistance gene mcr-3 in Aeromonas spp. from the national resistance monitoring programme GERM-Vet and from diagnostic submissions | 2005-2011 | Germany | Europe | Fish | N.A | *Aeromonas* | 479 | | 4 | mcr3 | 4 | | | | |
| **155** | 10.1016/j.jgar.2018.06.007 | Prevalence of colistin resistance gene mcr-1 in colistin-resistant Escherichia coli and Klebsiella pneumoniae isolated from humans in Thailand | 2014-2017 | Thailand | Europe | Human | N.A | P. *Escherichia coli* | 13 | 11 | | | 11 | | | | |
| | | Prevalence of colistin resistance gene mcr-1 in colistin-resistant Escherichia coli and Klebsiella pneumoniae isolated from humans in Thailand | 2014-2017 | Thailand | Europe | Human | N.A | *Klebsiella pneumoniae* | 213 | 4 | | | 4 | | | | |
| **156** | 10.1093/jac/dky292 | Molecular epidemiology of isolates with multiple mcr plasmids from a pig farm in Great Britain: the effects of colistin withdrawal in the short and long term | 2015-2017 | United Kingdom | Europe | Swine | Fecal | Non P. Escherichia coli | | 224 | | | 224 | | | | |

| | | | | | | | | | | | | | | | | | |
|---|---|---|---|---|---|---|---|---|---|---|---|---|---|---|---|---|---|
| | | Molecular epidemiology of isolates with multiple mcr plasmids from a pig farm in Great Britain: the effects of colistin withdrawal in the short and long term | 2015-2017 | United Kingdom | Europe | Swine | Fecal | Non P. Escherichia coli | | | 7 | mcr3 | 7 | 7 | IncP | | |
| **157** | 10.1016/j.scitotenv.2018.05.387 | Emergence of mcr-1 plasmid-mediated colistin-resistant Escherichia coli isolates from seawater | 2016 | Algeria | Africa | Seawater | Water | Non P. Escherichia coli | 246 | 2 | | | 2 | | | | |
| **158** | 10.1016/j.ijid.2018.02.006 | Microbiological surveillance of plasmid mediated colistin resistance in human Enterobacteriaceae isolates in Romagna (Northern Italy): August 2016–July 2017 | 2016-2017 | Italy | Europe | Human | N.A | P. *Escherichia coli* | 90 | 26 | | | 26 | | | 617 | 1 |
| | | Microbiological surveillance of plasmid mediated colistin resistance in human Enterobacteriaceae isolates in Romagna (Northern Italy): August 2016–July 2017 | | | | | | | | | | | | | | 744 | 1 |
| | | Microbiological surveillance of plasmid mediated colistin resistance in human Enterobacteriaceae isolates in Romagna (Northern Italy): August 2016–July 2017 | | | | | | | | | | | | | | 73 | 1 |
| | | Microbiological surveillance of plasmid mediated colistin resistance in human Enterobacteriaceae isolates in Romagna (Northern Italy): August 2016–July 2017 | | | | | | | | | | | | | | 410 | 1 |
| | | Microbiological surveillance of plasmid mediated colistin resistance in human Enterobacteriaceae isolates in Romagna (Northern Italy): August 2016–July 2017 | | | | | | | | | | | | | | 624 | 1 |
| | | Microbiological surveillance of plasmid mediated colistin resistance in human Enterobacteriaceae isolates in Romagna (Northern Italy): August 2016–July 2017 | | | | | | | | | | | | | | 10 | 1 |
| | | Microbiological surveillance of plasmid mediated colistin resistance in human Enterobacteriaceae isolates in Romagna (Northern Italy): August 2016–July 2017 | | | | | | | | | | | | | | 354 | 1 |
| | | Microbiological surveillance of plasmid mediated colistin resistance in human Enterobacteriaceae isolates in Romagna (Northern Italy): August 2016–July 2017 | | | | | | | | | | | | | | 216 | 1 |
| | | Microbiological surveillance of plasmid mediated colistin resistance in human Enterobacteriaceae isolates in Romagna (Northern Italy): August 2016–July 2017 | | | | | | | | | | | | | | 96 | 1 |

| | | | | | | | | | | | | | | | | | |
|---|---|---|---|---|---|---|---|---|---|---|---|---|---|---|---|---|---|
| | | Microbiological surveillance of plasmid mediated colistin resistance in human Enterobacteriaceae isolates in Romagna (Northern Italy): August 2016–July 2017 | | | | | | | | | | | | | | 224 | 1 |
| | | Microbiological surveillance of plasmid mediated colistin resistance in human Enterobacteriaceae isolates in Romagna (Northern Italy): August 2016–July 2017 | | | | | | | | | | | | | | 69 | 1 |
| | | Microbiological surveillance of plasmid mediated colistin resistance in human Enterobacteriaceae isolates in Romagna (Northern Italy): August 2016–July 2017 | | | | | | | | | | | | | | 457 | 1 |
| | | Microbiological surveillance of plasmid mediated colistin resistance in human Enterobacteriaceae isolates in Romagna (Northern Italy): August 2016–July 2017 | | | | | | | | | | | | | | 131 | 1 |
| **159** | 10.1089/mdr.2018.0110 | Prevalence of Extended-Spectrum Beta-Lactamase-Producing Gram-Negative Bacilli and Emergence of mcr-1 Colistin Resistance Gene in Lebanese Swine Farms | 2017 | Lebanon | Asia | Swine | Fecal | Non P. Escherichia coli | 111 | 23 | | | 23 | | | | |
| **160** | 10.1186/s12879-018-2987-y | Prospective study on human fecal carriage of Enterobacteriaceae possessing mcr-1 and mcr-2 genes in a regional hospital in Hong Kong | 2016 | China | Asia | Human | Fecal | P. *Escherichia coli* | | 14 | | | 14 | 3 | IncK | 442 | 1 |
| | | Prospective study on human fecal carriage of Enterobacteriaceae possessing mcr-1 and mcr-2 genes in a regional hospital in Hong Kong | | | | | | | | | | | | 3 | IncX4 | 88 | 1 |
| | | Prospective study on human fecal carriage of Enterobacteriaceae possessing mcr-1 and mcr-2 genes in a regional hospital in Hong Kong | | | | | | | | | | | | 1 | IncFII | 155 | 1 |
| | | Prospective study on human fecal carriage of Enterobacteriaceae possessing mcr-1 and mcr-2 genes in a regional hospital in Hong Kong | | | | | | | | | | | | 2 | IncP | 10 | 1 |
| | | Prospective study on human fecal carriage of Enterobacteriaceae possessing mcr-1 and mcr-2 genes in a regional hospital in Hong Kong | | | | | | | | | | | | 2 | Chromsomal | 34 | 1 |
| | | Prospective study on human fecal carriage of Enterobacteriaceae possessing mcr-1 and mcr-2 genes in a regional hospital in Hong Kong | | | | | | | | | | | | 2 | Incx | 226 | 1 |
| | | Prospective study on human fecal carriage of Enterobacteriaceae possessing mcr-1 and mcr-2 genes in a regional hospital in Hong Kong | | | | | | | | | | | | 1 | IncHI2 | 5995 | 1 |

| | | | | | | | | | | | | | | | | | |
|---|---|---|---|---|---|---|---|---|---|---|---|---|---|---|---|---|---|
| **161** | 10.1016/j.jgar.2017.09.014 | Potential transmission opportunity of CTX-M-producing Escherichia coli on a large-scale chicken farm in Vietnam | 2013-2014 | Vietnam | Asia | Poultry | N.A | P. *Escherichia coli* | 33 | 10 | | | 10 | | | | |
| | | Potential transmission opportunity of CTX-M-producing Escherichia coli on a large-scale chicken farm in Vietnam | 2013-2014 | Vietnam | Asia | Human | N.A | P. *Escherichia coli* | 20 | 1 | | | 1 | | | | |
| **162** | 10.1016/j.ijid.2018.05.011 | Emergence of mcr-1 and mcr-3 variants coding for plasmid-mediated colistin resistance in Escherichia coli isolates from food- producing animals in South Korea | 2014-2017 | South Korea | Asia | Swine | Fecal | P. *Escherichia coli* | 9 | 3 | | | 3 | | | | |
| | | Emergence of mcr-1 and mcr-3 variants coding for plasmid-mediated colistin resistance in Escherichia coli isolates from food- producing animals in South Korea | 2014-2017 | South Korea | Asia | Swine | Fecal | P. *Escherichia coli* | | | 2 | mcr3 | 2 | | | | |
| **163** | 10.3389/fmicb.2018.01217 | Molecular Epidemiology of mcr-Encoded Colistin Resistance in Enterobacteriaceae From Food-Producing Animals in Italy Revealed Through the EU Harmonized Antimicrobial Resistance Monitoring | 2014-2015 | Italy | Europe | Swine | Fecal | Non P. Escherichia coli | 678 | 47 | | | 47 | | | | |
| | | Molecular Epidemiology of mcr-Encoded Colistin Resistance in Enterobacteriaceae From Food-Producing Animals in Italy Revealed Through the EU Harmonized Antimicrobial Resistance Monitoring | 2014-2015 | Italy | Europe | Swine | Fecal | Non P. Escherichia coli | | | 1 | mcr4 | 1 | | | | |
| | | Molecular Epidemiology of mcr-Encoded Colistin Resistance in Enterobacteriaceae From Food-Producing Animals in Italy Revealed Through the EU Harmonized Antimicrobial Resistance Monitoring | 2014-2015 | Italy | Europe | N.A | Fecal | Non P. Escherichia coli | 861 | 86 | | | 86 | | | | |
| | | Molecular Epidemiology of mcr-Encoded Colistin Resistance in Enterobacteriaceae From Food-Producing Animals in Italy Revealed Through the EU Harmonized Antimicrobial Resistance Monitoring | 2014-2015 | Italy | Europe | Bovine | Fecal | Non P. Escherichia coli | | | 4 | mcr3 | 4 | | | | |
| | | Molecular Epidemiology of mcr-Encoded Colistin Resistance in Enterobacteriaceae From Food-Producing Animals in Italy Revealed Through the EU Harmonized Antimicrobial Resistance Monitoring | 2014-2015 | Italy | Europe | Bovine | Fecal | Non P. Escherichia coli | | | 1 | mcr4 | 1 | | | | |
| | | Molecular Epidemiology of mcr-Encoded Colistin Resistance in Enterobacteriaceae From Food-Producing Animals in Italy Revealed Through the EU Harmonized Antimicrobial Resistance Monitoring | 2014-2015 | Italy | Europe | Turkey | N.A | *Salmonella enterica* | 236 | 3 | | | 3 | | | | |

| | | | | | | | | | | | | | | | | | |
|---|---|---|---|---|---|---|---|---|---|---|---|---|---|---|---|---|---|
| **164** | 10.1038/s41426-018-0124-z | Emergence of a novel mobile colistin resistance gene, mcr-8,inNDM-producing Klebsiella pneumoniae | 2015-2017 | China | Asia | Swine | Fecal | *Klebsiella pneumoniae* | | | 1 | mcr8 | 1 | 1 | IncP | 42 | 1 |
| **165** | 10.1016/j.ijantimicag.2018.01.023 | The prevalence of colistin resistance in Escherichia coli and Klebsiella pneumoniae isolated from food animals in China: coexistence of mcr-1 and blaNDM with low fitness cost | 2016 | China | Asia | N.A | N.A | Non P. Escherichia coli | 373 | 100 | | | 100 | 14 | IncX | | |
| | | | 2016 | China | Asia | N.A | Fecal | *Klebsiella pneumoniae* | 54 | 2 | | | 2 | | | | |
| **166** | 10.1016/j.ijantimicag.2017.12.030 | Isolation of an IncP-1 plasmid harbouring mcr-1 from a chicken isolate of Citrobacter braakii in China | 2015 | China | Asia | Poultry | Fecal | *Citrobacter braakii* | 12 | 5 | | | 5 | | | | |
| **167** | 10.3389/fmicb.2018.00815 | A Novel mcr-1 Variant Carried by an IncI2-Type Plasmid Identified From a Multidrug Resistant Enterotoxigenic Escherichia coli | 2014 | China | Asia | Human | Fecal | P. *Escherichia coli* | 1 | 5 | | mcr1.9 | 5 | 6 | Chromsomal | | |
| **168** | 10.1099/jmm.0.000793 | A clinical isolate of Escherichia coli co-harbouring mcr-1 and blaNDM-5 in Japan | 2017 | Japan | Asia | Human | Fecal | P. *Escherichia coli* | | 1 | | | 1 | | | 132 | 1 |
| **169** | 10.1016/j.ijantimicag.2018.01.007 | Co-occurrence of mcr-1 in the chromosome and on an IncHI2 plasmid: persistence of colistin resistance in Escherichia coli | 2012 | China | Asia | Swine | Fecal | P. *Escherichia coli* | | 2 | | | 2 | 2 | IncHI2 | 1114 | 1 |
| | | | | | | | | | | | | | | | | 7314 | 1 |
| **170** | 10.1016/j.diagmicrobio.2017.12.015 | Spread of colistin resistance gene mcr-1 in Italy: characterization of the mcr-1.2 allelic variant in a colistin-resistant blood isolate of Escherichia coli | 2015-2017 | Italy | Europe | Human | Blood | P. *Escherichia coli* | | 2 | | mcr1.2 | 2 | 2 | IncX4 | 354 | |
| **171** | 10.1128/AAC.00501-18 | Colocation of the Polymyxin Resistance Gene mcr-1 and a Variant of mcr-3 on a Plasmid in an Escherichia coli Isolate from a Chicken Farm | 2015 | China | Asia | Poultry | Fecal | P. *Escherichia coli* | | 1 | | | 1 | 2 | IncK | | |
| | | Colocation of the Polymyxin Resistance Gene mcr-1 and a Variant of mcr-3 on a Plasmid in an Escherichia coli Isolate from a Chicken Farm | | China | Asia | Poultry | Fecal | P. *Escherichia coli* | | | 1 | mcr3.11 | 1 | 1 | IncF | | |
| **172** | 10.1016/j.ijantimicag.2018.07.007 | Co-occurrence of mcr-3 and bla NDM-5 genes in multidrug-resistant Klebsiella pneumoniae ST709 from a commercial chicken farm in China | 2016-2017 | China | Asia | Poultry | Fecal | *Klebsiella pneumoniae* | 30 | | 1 | mcr3.11 | 1 | | | | |
| **173** | 10.1128/AAC.00404-18 | Prevalence and Genetic Analysis of mcr-3-Positive Aeromonas Species from Humans, Retail Meat, and Environmental Water Samples | 2017 | China | Asia | Human | Fecal | *Aeromonas hydrophila* | | | 2 | mcr3 | 2 | | | | |
| | | Prevalence and Genetic Analysis of mcr-3-Positive Aeromonas Species from Humans, Retail Meat, and Environmental Water Samples | 2017 | China | Asia | Swine | Pork | *Aeromonas hydrophila* | | | 1 | mcr3 | 1 | | | | |
| | | Prevalence and Genetic Analysis of mcr-3-Positive Aeromonas Species | 2017 | China | Asia | Poultry | Chicken meat | *Aeromonas veronii* | | | 3 | mcr3 | 3 | | | | |

| | | | | | | | | | | | | | | | | | |
|---|---|---|---|---|---|---|---|---|---|---|---|---|---|---|---|---|---|
| | | from Humans, Retail Meat, and Environmental Water Samples | | | | | | | | | | | | | | | |
| | | Prevalence and Genetic Analysis of mcr-3-Positive Aeromonas Species from Humans, Retail Meat, and Environmental Water Samples | 2017 | China | Asia | Water | Water | *Aeromonas veronii* | | | 4 | mcr3 | 4 | | | | |
| **174** | 10.1186/s13099-018-0266-5 | Antimicrobial resistance in Enterobacteriaceae from healthy broilers in Egypt: emergence of colistin-resistant and extended-spectrum β-lactamase-producing Escherichia coli | 2016 | Egypt | Africa | Poultry | Fecal | P. *Escherichia coli* | 63 | 5 | | | 5 | | | | |
| **175** | 10.1089/mdr.2017.0026 | First Report of the Plasmid-Mediated Colistin Resistance Gene mcr-1 in Escherichia coli ST405 Isolated from Wildlife in Bejaia, Algeria | 2016 | Algeria | Africa | Barbary macaques | N.A | P. *Escherichia coli* | 1 | 1 | | | 1 | | | | |
| **176** | 10.1093/jac/dky272 | mcr-1 and mcr-2 (mcr-6.1) variant genes identified in Moraxella species isolated from pigs in Great Britain from 2014 to 2015 | 2018 | United Kingdom | Europe | Swine | Fecal | *Moraxella pluranimalium* | | | 1 | mcr6 | 1 | | | | |
| **177** | 10.1093/ofid/ofx115 | Detection of mcr-1-Carrying Escherichia coli Causing BloodstreamInfection in a New York City Hospital: Avian Origins, Human Concerns? Bloodstream Infection in a New York City Hospital: Avian Origins, Human Concerns? | 2015 | United States | North America | Human | Fecal | P. *Escherichia coli* | 1 | 1 | | | 1 | 1 | IncX4 | 117 | 1 |
| **178** | 10.1371/journal.pone.0178598 | Prevalence of colistin resistance gene (mcr-1) containing Enterobacteriaceae in feces of patients attending a tertiary care hospital and detection of a mcr-1 containing, colistin susceptible E. coli. | 2014 | Netherland | Europe | Human | Fecal | P. *Escherichia coli* | 2 | 2 | | | 2 | 2 | IncX4 | 359 | 2 |
| **179** | 10.1111/apm.12720 | First environmental sample containing plasmid-mediated colistin-resistant ESBL-producing Escherichia coli detected in Norway. | 2010 | Norway | Europe | Water | Water | Non P. Escherichia coli | 82 | 2 | | | 2 | | | 5340 | 1 |
| **180** | 10.1111/1348-0421.12549 | Detection of the *mcr-1* gene in colistin resistant *Escherichia coli* from retail meat in japan | 2015 | Japan | Asia | Poultry | Chicken meat | P. *Escherichia coli* | 154 | 7 | | | 7 | 7 | IncI2 | 5430 | 1 |
| | | Detection of the *mcr-1* gene in colistin resistant *Escherichia coli* from retail meat in japan | 2015 | Brazil | South America | Poultry | Chicken meat | P. *Escherichia coli* | | 2 | | | 2 | 2 | IncX4 | 297 | 1 |
| | | Detection of the *mcr-1* gene in colistin resistant *Escherichia coli* from retail meat in japan | 2015 | spain | Europe | Swine | Pork | P. *Escherichia coli* | 55 | 1 | | | 1 | 1 | IncX4 | 10 | 4 |
| | | Detection of the *mcr-1* gene in colistin resistant *Escherichia coli* from retail meat in japan | | | | | | | | | | | | | | 1011 | 1 |
| | | Detection of the *mcr-1* gene in colistin resistant *Escherichia coli* from retail meat in japan | | | | | | | | | | | | | | 117 | 1 |

| | | | | | | | | | | | | | | | | | |
|---|---|---|---|---|---|---|---|---|---|---|---|---|---|---|---|---|---|
| | | Detection of the *mcr-1* gene in colistin resistant *Escherichia coli* from retail meat in japan | | | | | | | | | | | | | | 155 | 1 |
| | | Detection of the *mcr-1* gene in colistin resistant *Escherichia coli* from retail meat in japan | | | | | | | | | | | | | | 48 | 1 |
| | | Detection of the *mcr-1* gene in colistin resistant *Escherichia coli* from retail meat in japan | | | | | | | | | | | | | | 2690 | 1 |
| **181** | 10.1371/journal.pone.0180347 | Molecular characteristics of mcr-1-carrying plasmids and new mcr-1 variant recovered from polyclonal clinical Escherichia coli from Argentina and Canada | 2012-2016 | Argentina | South America | Human | Blood, urine, abdomen | P. *Escherichia coli* | 87 | 5 | | | 5 | 5 | IncI2 | 6 | 1 |
| | | Molecular characteristics of mcr-1-carrying plasmids and new mcr-1 variant recovered from polyclonal clinical Escherichia coli from Argentina and Canada | 2012-2016 | Argentina | South America | Human | Blood, urine, abdomen | P. *Escherichia coli* | | 3 | | mcr1.5 | 3 | 3 | IncI2 | 49 | 1 |
| | | Molecular characteristics of mcr-1-carrying plasmids and new mcr-1 variant recovered from polyclonal clinical Escherichia coli from Argentina and Canada | 2011 | Canada | North America | Human | Blood, urine, abdomen | P. *Escherichia coli* | 1 | 1 | | | 1 | 1 | IncI2 | 624 | 1 |
| | | Molecular characteristics of mcr-1-carrying plasmids and new mcr-1 variant recovered from polyclonal clinical Escherichia coli from Argentina and Canada | | | | | | | | | | | | | | 196 | 1 |
| | | Molecular characteristics of mcr-1-carrying plasmids and new mcr-1 variant recovered from polyclonal clinical Escherichia coli from Argentina and Canada | | | | | | | | | | | | | | 881 | 1 |
| | | Molecular characteristics of mcr-1-carrying plasmids and new mcr-1 variant recovered from polyclonal clinical Escherichia coli from Argentina and Canada | | | | | | | | | | | | | | 614 | 1 |
| | | Molecular characteristics of mcr-1-carrying plasmids and new mcr-1 variant recovered from polyclonal clinical Escherichia coli from Argentina and Canada | | | | | | | | | | | | | | 675 | 1 |
| | | Molecular characteristics of mcr-1-carrying plasmids and new mcr-1 variant recovered from polyclonal clinical Escherichia coli from Argentina and Canada | | | | | | | | | | | | | | 600 | 1 |
| | | | | | | | | | | | | | | | | 615 | 1 |
| | | | | | | | | | | | | | | | | 410 | 1 |
| | | | | | | | | | | | | | | | | 4110 | 1 |

| | | | | | | | | | | | | | | | | | |
|---|---|---|---|---|---|---|---|---|---|---|---|---|---|---|---|---|---|
| **182** | 10.1128/AAC.00017-17 | Occurrence of Plasmid- and Chromosome-Carried mcr-1 in Waterborne Enterobacteriaceae in China. | 2016 | China | Asia | Water | Water | Non P. Escherichia coli | 16 | 16 | | | 16 | 3 | Chromsomal | | |
| | | Occurrence of Plasmid- and Chromosome-Carried mcr-1 in Waterborne Enterobacteriaceae in China. | 2016 | China | Asia | Water | Water | *Citrobacter freundii* | | 2 | | | 2 | 1 | IncI2 | | |
| | | Occurrence of Plasmid- and Chromosome-Carried mcr-1 in Waterborne Enterobacteriaceae in China. | 2016 | China | Asia | Water | Water | *Klebsiella oxytoca* | | 2 | | | 2 | 1 | IncX4 | | |
| | | Occurrence of Plasmid- and Chromosome-Carried mcr-1 in Waterborne Enterobacteriaceae in China. | 2016 | China | Asia | Water | Water | Citrobacter braakii | | 2 | | | 2 | 2 | IncHI2 | | |
| | | Occurrence of Plasmid- and Chromosome-Carried mcr-1 in Waterborne Enterobacteriaceae in China. | 2016 | China | Asia | Water | Water | *Enterobacter cloacae* | | 1 | | | 1 | 2 | IncHI2 | | |
| **183** | 10.12007/j.issn.0258-4646.2018.08.010 | Screening for the mcr-1 Gene in the Members of the *Enterobacteriaceae* and Its Correlation with Colistin Resistant Phenotype | 2013-2015 | China | Asia | Human | N.A | P. *Escherichia coli* | 1421 | 20 | | | 20 | 2 | IncI2 | | |
| | | | 2013-2015 | China | Asia | Human | N.A | *Klebsiella pneumoniae* | 734 | 6 | | | 6 | 1 | IncHI2 | | |
| **184** | 10.13699/j.cnki.1001-6821.2018.14.032 | Study of a clinical extensive drug resistant *Escherichia coli* isolate coexistence of mcr-1 and NDM-6 | 2015 | China | Asia | Human | Urine | P. *Escherichia coli* | | 1 | | | 1 | 1 | | 156 | 1 |
| **185** | 10.7506/spkx1002-6630-201814022 | Drug Resistance Analysis of a Multidrug-Resistant *Proteus mirabilis* Carrying the mcr-1 Gene Isolated from Food-Producing Animals | 2015 | China | Asia | Human | Urine | *Proteus mirabilis* | 361 | 1 | | | 1 | 1 | IncI2 | | |
| **186** | 10.3784/j.issn.1003-9961.2017.05.005 | Polymyxin resistance and mcr-1 prevalence in non-typhoid *Salmonella* isolates in China | 2011-2014 | China | Asia | Human | Fecal | *Salmonella enterica* serovar Typhimurium | 404 | 1 | | | 1 | 1 | | | |
| **187** | 10.13590/j.cjfh.2018.05.002 | Resistance Characteristic Analaysis for Foodborne *Salmonella* Isolates from China, 26 Provinence Study | 2016 | China | Asia | | Food | *Salmonella enterica* serovar Typhimurium | 755 | 1 | | | 1 | | | | |
| | | | | China | Asia | | Food | *Salmonella enterica* serovar Derby | | 1 | | | 1 | | | | |
| **188** | 10.13590/j.cjfh.2017.06.003 | Resistance Analysis of 1070 *Salmonella* strains isolated from food sample in mainland China, 2015 | 2015 | China | Asia | | Food | *Salmonella enterica* serovar London | 1070 | 1 | | | 1 | | | | |
| **189** | 10.16303/j.cnki.1005-4545.2018.03.09 | Isolation and identification of *Escherichia coli* with resistance to colistin from swine of different growth stages | 2016 | China | Asia | Swine | Fecal | Non P. Escherichia coli | 69 | 32 | | | 32 | | IncFIB | | |

| | | | | | | | | | | | | | | | | | |
|---|---|---|---|---|---|---|---|---|---|---|---|---|---|---|---|---|---|
| | | Isolation and identification of *Escherichia coli* with resistance to colistin from swine of different growth stages | 2016 | China | Asia | Swine | Fecal | Non P. Escherichia coli | | 14 | | | 14 | | IncY | | |
| | | Isolation and identification of *Escherichia coli* with resistance to colistin from swine of different growth stages | 2016 | China | Asia | Swine | Fecal | Non P. Escherichia coli | | 7 | | | 7 | | IncFIA | | |
| | | Isolation and identification of *Escherichia coli* with resistance to colistin from swine of different growth stages | 2016 | China | Asia | Swine | Fecal | Non P. Escherichia coli | | 4 | | | 4 | | IncH | | |
| | | Isolation and identification of *Escherichia coli* with resistance to colistin from swine of different growth stages | 2016 | China | Asia | Swine | Fecal | Non P. Escherichia coli | | 3 | | | 3 | | IncFIA | | |
| | | Isolation and identification of *Escherichia coli* with resistance to colistin from swine of different growth stages | 2016 | China | Asia | Swine | Fecal | Non P. Escherichia coli | | 2 | | | 2 | | IncFIC | | |
| | | Isolation and identification of *Escherichia coli* with resistance to colistin from swine of different growth stages | 2016 | China | Asia | Swine | Fecal | Non P. Escherichia coli | | 1 | | | 1 | | IncHI1 | | |
| | | Isolation and identification of *Escherichia coli* with resistance to colistin from swine of different growth stages | 2016 | China | Asia | Swine | Fecal | Non P. Escherichia coli | | 1 | | | 1 | | IncL/M | | |
| **190** | thesis:YZU2017 XiaoweiChen | The analysis of antimicrobial resistance and the plasmid mediated resistant genes in *Salmonella* Typhimurium isolates | 2014-2016 | China | Asia | Human | Fecal | *Salmonella enterica* serovar Typhimurium, | 330 | 1 | | | 1 | 5 | | 34 | 1 |
| **191** | 10.1016/j.vetmic.2018.09.018 | High carriage rate of mcr-1 and antimicrobial resistance profiles of mcr-1-positive Escherichia coli isolates in swine faecal samples collected from eighteen provinces in China | 2016-2017 | China | Asia | Swine | Fecal | Non P. Escherichia coli | 220 | 152 | | | 152 | | | | |
| **192** | 10.2147/IDR.S166726 | Prevalence and molecular characteristics of mcr-1 colistin resistance in Escherichia coli: isolates of clinical infection from a Chinese University Hospital | 2015-2016 | China | Asia | Human | Urine, pus, sputum | P. *Escherichia coli* | 1112 | 6 | | | 6 | 3 | IncX4 | 156 | 1 |
| | | Prevalence and molecular characteristics of mcr-1 colistin resistance in Escherichia coli: isolates of clinical infection from a Chinese University Hospital | | | | | | | | | | | | | | 457 | 1 |
| | | Prevalence and molecular characteristics of mcr-1 colistin resistance in Escherichia coli: isolates of clinical infection from a Chinese University Hospital | | | | | | | | | | | | | | 160 | 1 |

| | | | | | | | | | | | | | | | | | |
|---|---|---|---|---|---|---|---|---|---|---|---|---|---|---|---|---|---|
| | | Prevalence and molecular characteristics of mcr-1 colistin resistance in Escherichia coli: isolates of clinical infection from a Chinese University Hospital | | | | | | | | | | | | | | 6706 | 1 |
| | | Prevalence and molecular characteristics of mcr-1 colistin resistance in Escherichia coli: isolates of clinical infection from a Chinese University Hospital | | | | | | | | | | | | | | 2847 | 1 |
| | | Prevalence and molecular characteristics of mcr-1 colistin resistance in Escherichia coli: isolates of clinical infection from a Chinese University Hospital | | | | | | | | | | | | | | 345 | 1 |
| **193** | 10.1016/j.jgar.2018.09.013 | First report of Escherichia coli carrying the mobile colistin resistance gene mcr-1 in Turkey | 2018 | Turkey | Asia | Poultry | Chicken meat | P. *Escherichia coli* | 4 | 4 | | | 4 | 4 | IncX4 | 1094 | 1 |
| | | First report of Escherichia coli carrying the mobile colistin resistance gene mcr-1 in Turkey | | | | | | | | | | | | | | 3941 | 1 |
| **194** | 10.1016/j.jgar.2018.09.005 | Detection of chromosomal and plasmid mediated mechanisms conferring colistin resistance in Escherichia coli and Klebsiella pneumoniae from Indian food samples | 2017 | India | Asia | Poultry | Chicken meat | P. *Escherichia coli* | 71 | 2 | | | 2 | 1 | IncHI2/HI2A | | |
| | | Detection of chromosomal and plasmid mediated mechanisms conferring colistin resistance in Escherichia coli and Klebsiella pneumoniae from Indian food samples | 2017 | India | Asia | Ovine | Mutton | P. *Escherichia coli* | | 1 | | | 1 | 1 | IncHI2 | | |
| | | Detection of chromosomal and plasmid mediated mechanisms conferring colistin resistance in Escherichia coli and Klebsiella pneumoniae from Indian food samples | | | | | | | | | | | | 1 | IncX1 | | |
| **195** | 10.1089/mdr.2017.0391 | Characterization of Highly Prevalent Plasmids Coharboring mcr-1, oqxAB, and blaCTX-M and Plasmids Harboring oqxAB and blaCTX-M in Escherichia coli Isolates from Food-Producing Animals in China | 2015-2017 | China | Asia | Swine | Fecal | P. *Escherichia coli* | 73 | 23 | | | 23 | 9 | IncHI2 | 3 | 10 |
| | | Characterization of Highly Prevalent Plasmids Coharboring mcr-1, oqxAB, and blaCTX-M and Plasmids Harboring oqxAB and blaCTX-M in Escherichia coli Isolates from Food-Producing Animals in China | 2015-2017 | China | Asia | Poultry | Fecal | P. *Escherichia coli* | 420 | 117 | | | 117 | | | | |
| **196** | 10.3389/fmicb.2018.02395 | Colistin Resistance Mediated by mcr-1 in ESBL-Producing, Multidrug | 2016-2017 | Italy | Europe | Poultry | N.A | *Salmonella enterica* | 320 | 3 | | | 3 | 4 | IncX4 | | |

| | | | | | | | | | | | | | | | | | |
|---|---|---|---|---|---|---|---|---|---|---|---|---|---|---|---|---|---|
| | | Resistant Salmonella Infantis in Broiler Chicken Industry, Italy (2016-2017) | | | | | | serovar Infantis | | | | | | | | | |
| | | Colistin Resistance Mediated by mcr-1 in ESBL-Producing, Multidrug Resistant Salmonella Infantis in Broiler Chicken Industry, Italy (2016-2017) | 2016-2017 | Italy | Europe | Poultry | Chicken meat | *Salmonella enterica* serovar Infantis | | 1 | | | 1 | | | | |
| **197** | 10.1016/j.ijantimicag.2018.08.015 | Clinical characteristics of patients with bacteraemia due to the emergence of mcr-1-harbouring Enterobacteriaceae in humans and pigs in Taiwan | 2017 | China | Asia | Human | Blood | P. *Escherichia coli* | 686 | 6 | | | 6 | | | | |
| | | Clinical characteristics of patients with bacteraemia due to the emergence of mcr-1-harbouring Enterobacteriaceae in humans and pigs in Taiwan | 2017 | China | Asia | Human | blood | *Klebsiella pneumoniae* | 673 | 3 | | | 3 | | | | |
| | | Clinical characteristics of patients with bacteraemia due to the emergence of mcr-1-harbouring Enterobacteriaceae in humans and pigs in Taiwan | 2017 | China | Asia | Human | Blood, Fecal | *Salmonella* | 221 | 1 | | | 1 | | | | |
| | | Clinical characteristics of patients with bacteraemia due to the emergence of mcr-1-harbouring Enterobacteriaceae in humans and pigs in Taiwan | 2017 | China | Asia | Swine | N.A | P. *Escherichia coli* | 16 | 12 | | | 12 | | | | |
| **198** | 10.3201/eid2409.171386 | Spread of mcr-1-Driven Colistin Resistance on Hospital Surfaces, Italy | 2016-2017 | Italy | Europe | Human | N.A | *Acinetobacter Iwoffii* | 300 | 4 | | | 4 | | | | |
| | | Spread of mcr-1-Driven Colistin Resistance on Hospital Surfaces, Italy | 2016-2017 | Italy | Europe | Human | N.A | *Enterobacter agglomerans* | | 4 | | | 4 | | | | |
| | | Spread of mcr-1-Driven Colistin Resistance on Hospital Surfaces, Italy | 2016-2017 | Italy | Europe | Human | N.A | *Klebsiella pneumoniae* | | 6 | | | 6 | | | | |
| | | Spread of mcr-1-Driven Colistin Resistance on Hospital Surfaces, Italy | 2016-2017 | Italy | Europe | Human | N.A | *Pseudomonas aeruginosa* | | 1 | | | 1 | | | | |
| | | Spread of mcr-1-Driven Colistin Resistance on Hospital Surfaces, Italy | 2016-2017 | Italy | Europe | Human | N.A | *Enterobacter cloacae* | | 3 | | | 3 | | | | |
| | | Spread of mcr-1-Driven Colistin Resistance on Hospital Surfaces, Italy | 2016-2017 | Italy | Europe | Human | N.A | *Citrobacter freundii* | | 1 | | | 1 | | | | |
| | | Spread of mcr-1-Driven Colistin Resistance on Hospital Surfaces, Italy | 2016-2017 | Italy | Europe | Human | N.A | P. *Escherichia coli* | | | | | | | | | |
| | | Spread of mcr-1-Driven Colistin Resistance on Hospital Surfaces, Italy | 2016-2017 | Italy | Europe | Human | N.A | *Pseudomonas putida* | | 1 | | | 1 | | | | |
| | | Spread of mcr-1-Driven Colistin Resistance on Hospital Surfaces, Italy | 2016-2017 | Italy | Europe | Human | N.A | *Klebsiella oxytoca* | | 2 | | | 2 | | | | |
| **199** | 10.3389/fmicb.2018.01679 | Simultaneous Carriage of mcr-1 and Other Antimicrobial Resistance Determinants in Escherichia coli From Poultry | 2017 | Argentina | South America | Poultry | Fecal | Non P. Escherichia coli | 304 | 41 | | | 41 | | | | |
| **200** | 10.1038/s41564-018-0205-8 | Anthropogenic and environmental factors associated with high incidence of mcr-1 carriage in humans across China | 2016 | China | Asia | Human | Fecal | Non P. Escherichia coli | 774 | 774 | | | 774 | | | | |
| **201** | 10.3343/alm.2018.38.6.555 | Detection of mcr-1 Plasmids in Enterobacteriaceae Isolates From Human Specimens: Comparison With Those in Escherichia coli Isolates From Livestock in Korea | 2010-2015 | South Korea | Asia | Human | N.A | P. *Escherichia coli* | 565 | 2 | | | 2 | 2 | IncI2 | | |

| | | | | | | | | | | | | | | | | | |
|---|---|---|---|---|---|---|---|---|---|---|---|---|---|---|---|---|---|
| | | | 2010-2015 | South Korea | Asia | Human | N.A | *Enterobacter aerogenes* | 887 | 1 | | | 1 | | | | |
| **202** | 10.1128/AAC.02623-17 | Prevalence and Molecular Characterization of Escherichia coli Clinical Isolates Carrying mcr-1 in a Chinese Teaching Hospital from 2002 to 2016 | 2002-2016 | China | Asia | Human | Blood, urine, sputum, wound, blood drainage | P. *Escherichia coli* | 3434 | 12 | | | 12 | | | 1 | 1 |
| | | Prevalence and Molecular Characterization of Escherichia coli Clinical Isolates Carrying mcr-1 in a Chinese Teaching Hospital from 2002 to 2016 | | | | | | | | | | | | | | 2 | 1 |
| | | Prevalence and Molecular Characterization of Escherichia coli Clinical Isolates Carrying mcr-1 in a Chinese Teaching Hospital from 2002 to 2016 | | | | | | | | | | | | | | 45 | 1 |
| | | Prevalence and Molecular Characterization of Escherichia coli Clinical Isolates Carrying mcr-1 in a Chinese Teaching Hospital from 2002 to 2016 | | | | | | | | | | | | | | 46 | 1 |
| | | Prevalence and Molecular Characterization of Escherichia coli Clinical Isolates Carrying mcr-1 in a Chinese Teaching Hospital from 2002 to 2016 | | | | | | | | | | | | | | 48 | 1 |
| | | Prevalence and Molecular Characterization of Escherichia coli Clinical Isolates Carrying mcr-1 in a Chinese Teaching Hospital from 2002 to 2016 | | | | | | | | | | | | | | 730 | 1 |
| | | Prevalence and Molecular Characterization of Escherichia coli Clinical Isolates Carrying mcr-1 in a Chinese Teaching Hospital from 2002 to 2016 | | | | | | | | | | | | | | 31 | 1 |
| | | Prevalence and Molecular Characterization of Escherichia coli Clinical Isolates Carrying mcr-1 in a Chinese Teaching Hospital from 2002 to 2016 | | | | | | | | | | | | | | 632 | 1 |
| | | Prevalence and Molecular Characterization of Escherichia coli Clinical Isolates Carrying mcr-1 in a Chinese Teaching Hospital from 2002 to 2016 | | | | | | | | | | | | | | 53 | 1 |
| | | Prevalence and Molecular Characterization of Escherichia coli Clinical Isolates Carrying mcr-1 in a Chinese Teaching Hospital from 2002 to 2016 | | | | | | | | | | | | | | 506 | 1 |
| | | | | | | | | | **110155** | **4917** | **274** | | **5191** | | | | |

N.B: The empty cells refer to the detailed extracted data in each study.

Supplemental table 3. Frequency of bacteria harboring the different *mcr* types

| Isolates | *mcr*-1 | *mcr*-2 | *mcr*-3 | *mcr*-4 | *mcr*-5 | *mcr*- 6 | *mcr*-7 | *mcr*-8 | Total |
|---|---|---|---|---|---|---|---|---|---|
| **Enterobacter** | | | | | | | | | |
| **E. aerogenes** | 2 | | | | | | | | 2 |
| **E. cloacae** | 11 | | | | | | | | 11 |
| **E. agglomerans** | 4 | | | | | | | | 4 |
| **Acinetobacter** | | | | | | | | | |
| **A. Iwoffii** | 4 | | | | | | | | 4 |
| **N.A** | 1 | | | | | | | | 1 |
| **Aeromonas** | | | | | | | | | |
| **A. hydrophila** | | | 3 | | 1 | | | | 4 |
| **A. veronii** | | | 17 | | | | | | 17 |
| **N.A** | | | 12 | | | | | | **12** |
| **Citrobacter** | | | | | | | | | |
| **C. freundii** | 4 | | | | | | | | 4 |
| **C. braakii** | 3 | | | | | | | | 3 |
| **Cronobacter** | **2** | | | | | | | | **2** |
| **C. sakazakii** | 2 | | | | | | | | 2 |
| **Klebsiellae** | | | | | | | | | |
| **K. oxytoca** | 4 | | | | | | | | 4 |
| **K. pneumoniae** | 126 | | 1 | | | | 1 | 1 | 129 |
| **K. variicola** | 3 | | | | | | | | **3** |
| **Kluyvera** | | | | | | | | | |
| **K. ascorbata** | 1 | | | | | | | | 1 |
| **Moraxella** | | | | | | | | | |
| **M. pluranimalium** | 1 | 1 | | | | 1 | | | 3 |
| **Non virulent Escherichia coli** | | | | | | | | | |
| **N.A** | **1978** | | 18 | 2 | | | | | 1998 |
| **P. Escherichia coli** | | | | | | | | | |
| **O157** | 1 | | | | | | | | 1 |
| **O29** | 1 | | | | | | | | **1** |
| **ONT:H32** | 1 | | | | | | | | **1** |
| **ONT:H55** | 3 | | | | | | | | **3** |
| **O9:H4** | | | | | | | | | **0** |
| **O54:H32** | | | | | | | | | **0** |
| **ONT:H9** | 1 | | | | | | | | 1 |
| **N.A** | 2615 | 6 | 65 | 124 | 5 | | | | 2815 |
| **Proteus** | | | | | | | | | |
| **P. mirabilis** | 1 | | | | | | | | 1 |
| **Pseudomonas** | | | | | | | | | |
| **P. putida** | 1 | | | | | | | | 1 |
| **P. aeruginosa** | 1 | | | | | | | | 1 |
| **P. alcalifaciens** | 1 | | | | | | | | **1** |
| **Raoultella** | | | | | | | | | |
| **R. ornithinolytica** | 2 | | | | | | | | **2** |
| **R. planticola** | 1 | | | | | | | | 1 |
| **Salmonella enterica** | | | | | | | | | |
| **serovar 1,4,[5],12:i:-** | 1 | | | | | | | | **1** |
| **seorvar Derby** | 2 | 1 | | | | | | | **3** |
| **seorvar Typhimurium** | 93 | | | 1 | | | | | 94 |
| **serovar Indiana** | 1 | | | | | | | | 1 |
| **serovar Infantis** | 4 | | | | | | | | **4** |
| **serovar Paratyphi B** | 2 | | | | 14 | | | | 16 |
| **serovar Paratyphi B var Java** | 4 | | | | | | | | 4 |
| **serovar Paratyphi B var Java phage type Colindale** | 2 | | | | | | | | 2 |
| **serovar Rissen** | 1 | | | | | | | | 1 |
| **serovar Virchow** | 3 | | | | | | | | 3 |
| **serovar London** | 1 | | | | | | | | 1 |
| **N.A** | 8 | | | | | | | | 8 |
| **Shigellae** | | | | | | | | | |
| **Shigella sonnei** | 7 | | | | | | | | 7 |
| **Total** | **4917** | **8** | **116** | **127** | **20** | **1** | **1** | **1** | **5191** |

Supplemental table 4. Numbers of articles mentioned *mcr* -carrying strains isolation methods.

| **Methods of isolation** | **Number of articles** |
|---|---|
| **Targeted PCR** | 98 |
| **Culture on selective media** | 36 |

Supplemental table 4. Country- level frequency of bacteria harboring *mcr* genes

| | CHI | LBN | JPN | VIE | THI | LAO | PAK | KOR | MAS | IND | TUR | SGP | BRN | CAM | KSA | OMA | UAE | ITA | UK | ESP | FRA | POR | GER | BEL | SUI | NED | DEN | NOR | EST | AUT | FIN | LTU | HUN | POL | TUN | RSA | EGY | ALG | BRA | ARG | COL | VEN | BOL | ECU | US | CAN | AUS |
|---|---|---|---|---|---|---|---|---|---|---|---|---|---|---|---|---|---|---|---|---|---|---|---|---|---|---|---|---|---|---|---|---|---|---|---|---|---|---|---|---|---|---|---|---|---|---|---|
| **P. *E. coli*** | | | | | | | | | | | | | | | | | | | | | | | | | | | | | | | | | | | | | | | | | | | | | | | |
| O157 | | | | | | | | | | | | | | | | | | | | | | | | | | | | | | | | | | | | | | | | | | | | | 1 | | |
| O29 | | | | | | | | | | | | | | | | | | | | | | | | | | | | | | | | | | | | | | 1 | | | | | | | | | |
| ONT:H32 | | | | | | | | | | | | | | | | | | | | | | | | | | | | | | | | | | | | | | | **1** | | | | | | | | |
| ONT:H5, O9:H4, O54:H32 | | | | | | | | | | | | | | | | | | | | | | | | | | | | | | | | | | | | | | | **3** | | | | | | | | |
| ONT:H9 | | | | | | | | | | | | | | | | | | | | | | | | | | | | | | | | | | | | | | | **1** | | | | | | | | |
| N.A | **1937** | | **99** | **15** | **14** | **9** | **1** | **8** | **7** | **3** | **4** | **4** | **2** | **1** | **1** | **1** | **1** | **69** | **12** | **212** | **117** | **94** | **4** | **19** | **17** | **11** | | **1** | **3** | **1** | **1** | **1** | **1** | **1** | **39** | **26** | **11** | **2** | **19** | **13** | **8** | **2** | | **1** | **6** | **4** | **2** |
| **Salmonella enterica** | | | | | | | | | | | | | | | | | | | | | | | | | | | | | | | | | | | | | | | | | | | | | | | |
| serovar 1,4,[5],12:i:- | **1** | | | | | | | | | | | | | | | | | | | | | | | | | | | | | | | | | | | | | | | | | | | | | | |
| serovar Paratyphi B | **2** | | | | | | | | | | | | | | | | | | | | | | **14** | | | | | | | | | | | | | | | | | | | | | | | | |
| seorvar Derby | **1** | | | | | | | | | | | | | | | | | | | | | | | | | | | | | | | | | | | | | | | | | | | | | | |
| seorvar Typhimurium | **77** | | | | | | | | | | | | | | | | | **1** | **2** | **3** | | **1** | | **2** | | | | | | | | | | | | | | | | | **3** | | | | | | |
| serovar Indiana | **1** | | | | | | | | | | | | | | | | | | | | | | | | | | | | | | | | | | | | | | | | | | | | | | |
| serovar Infantis | | | | | | | | | | | | | | | | | | **4** | | | | | | | | | | | | | | | | | | | | | | | | | | | | | |
| Serovar Paratyphi B var Java | | | | | | | | | | | | | | | | | | | **4** | | | | | | | | | | | | | | | | | | | | | | | | | | | | |
| serovar Paratyphi B var Java phage type Colindale | | | | | | | | | | | | | | | | | | | **2** | | | | | | | | | | | | | | | | | | | | | | | | | | | | |
| serovar Rissen | | | | | | | | | | | | | | | | | | | | **1** | | | | | | | | | | | | | | | | | | | | | | | | | | | |
| serovar Typh.variant copenhagen | | | | | | | | | | | | | | | | | | | **1** | | | | | | | | | | | | | | | | | | | | | | | | | | | | |
| serovar London | **1** | | | | | | | | | | | | | | | | | | | | | | | | | | | | | | | | | | | | | | | | | | | | | | |
| serovar Virchow | | | | | | | | | | | | | | | | | | | **3** | | | | | | | | | | | | | | | | | | | | | | | | | | | | |
| N.A | **5** | | | | | | | | | | | | | | | | | **3** | **1** | | | | | **1** | | | | | | | | | | | | | | | | | | | | | | | |
| **Citrobacter** | | | | | | | | | | | | | | | | | | | | | | | | | | | | | | | | | | | | | | | | | | | | | | | |
| C. freundii | **3** | | | | | | | | | | | | | | | | | **1** | | | | | | | | | | | | | | | | | | | | | | | | | | | | | |
| C. braakii | **7** | | | | | | | | | | | | | | | | | | | | | | | | | | | | | | | | | | | | | | | | | | **1** | | | | |
| ***Enterobacter*** | | | | | | | | | | | | | | | | | | | | | | | | | | | | | | | | | | | | | | | | | | | | | | | |
| E. aerogenes | **1** | | | | | | | 1 | | | | | | | | | | | | | | | | | | | | | | | | | | | | | | | | | | | | | | | |
| E. cloacae | **8** | | | | | | | | | | | | | | | | | **3** | | | | | | | | | | | | | | | | | | | | | | | | | | | | | |
| E. agglomerans | | | | | | | | | | | | | | | | | | **4** | | | | | | | | | | | | | | | | | | | | | | | | | | | | | |

| | | | | | | | | | | | | | | | | | | | | | | |
|---|---|---|---|---|---|---|---|---|---|---|---|---|---|---|---|---|---|---|---|---|---|---|
| **Acinetobacter** | | | | | | | | | | | | | | | | | | | | | | |
| A. Iwoffii | | | | | | | | **4** | | | | | | | | | | | | | | |
| N.A | **1** | | | | | | | | | | | | | | | | | | | | | |
| **Aeromonas** | | | | | | | | | | | | | | | | | | | | | | |
| A. hydrophila | **4** | | | | | | | | | | | | | | | | | | | | | |
| A. veronii | **17** | | | | | | | | | | | | | | | | | | | | | |
| N.A | **8** | | | | | | | | | | | | **4** | | | | | | | | | |
| **Cronobacter** | | | | | | | | | | | | | | | | | | | | | | |
| C. sakazakii | **2** | | | | | | | | | | | | | | | | | | | | | |
| ***Klebsiellae*** | | | | | | | | | | | | | | | | | | | | | | |
| K. oxytoca | **2** | | | | | | | | | | | | | | | | | | | | | |
| K. *pneumoniae* | **63** | | **5** | | **6** | | **4** | **2** | | **1** | **1** | **32** | | | | | | **1** | | **1** | **2** | |
| K. *variicola* | **3** | | | | | | | **8** | | | | | | | | | | | | | | |
| **Kluyvera ascorbata** | 1 | | | | | | | | | | | | | | | | | | | | | |
| **Moraxella pluranimalium** | | | | | | | | | **1** | **2** | | | | | | | | | | | | |
| **Non p. *E. coli*** | **1215** | **111** | **39** | | **71** | **8** | | **139** | **231** | **23** | **34** | | **64** | **3** | **5** | **2** | **2** | **10** | **41** | | | |
| **Proteus mirabilis** | 1 | | | | | | | | | | | | | | | | | | | | | |
| **Pseudomonas** | | | | | | | | | | | | | | | | | | | | | | |
| P. aeruginosa | | | | | | | | **1** | | | | | | | | | | | | | | |
| P. alcalifaciens | 1 | | | | | | | | | | | | | | | | | | | | | |
| P.putida | | | | | | | | **1** | | | | | | | | | | | | | | |
| **Raoultella** | | | | | | | | | | | | | | | | | | | | | | |
| R Ornithinolytica | **2** | | | | | | | | | | | | | | | | | | | | | |
| R. planticola | 1 | | | | | | | | | | | | | | | | | | | | | |
| ***Shigella sonnei*** | **6** | | | **1** | | | | | | | | | | | | | | | | | | |

Countries: (CHI) China, (JPN) Japan, (LBN) Lebanon, (VIE) Vietnam, (THA) Thailand, (LAO) Laos, (PAK) Pakistan, (MAS) Malaysia, (KOR) South Korea, (IND) India, (TUR) Turkey, (SGP) Singapore, (BRN) Bahrain, (CAM) Cambodia, (KSA) Kingdom of Saudi Arabia, (OMA) Oman, (UAE) United Arab of Emirates, (UK) United Kingdom, (ESP) Spain, (ITA) Italy, (FRA) France, (POR) Portugal, (GER) Germany, (BEL) Belgium, (SUI) Switzerland, (NED) Netherland, (DEN) Denmark, (NOR) Norway, (EST) Estonia, (AUT) Austria, (FIN) Finland, (POL) Poland, (LTU) Lithuania, (HUN) Hungary, (TUN) Tunisia, (RSA) South Africa, (EGY) Egypt, (ALG) Algeria, (BRA) Brazil, (ARG) Argentina, (COL) Colombia, (VEN) Venezuela, (BOL) Bolivia, (ECU) Ecuador, (U.S) United States, (CAN) Canada, (AUS) Australia

Supplemental table 5. **Estimated clinical *mcr* -carrying isolates**

| Continents | Countries | Fecal | Blood | Urine | Others | Total | Diseased | Asymptomatic |
|---|---|---|---|---|---|---|---|---|
| **Asia** | **China** | 1438 | 32 | 7 | 234 | 1711 | 966 | 781 |
| | **Japan** | 97 | | | | 97 | 97 | |
| | **Thailand** | 2 | | 1 | 17 | 20 | 20 | |
| | **Laos** | 6 | | | | 6 | 6 | |
| | **South Korea** | 1 | | | 3 | 4 | 4 | |
| | **India** | | | | 4 | 4 | 4 | |
| | **Singapore** | | | 2 | 2 | 4 | 4 | |
| | **Vietnam** | 1 | | | 2 | 3 | 3 | |
| | **Bahrain** | | | | 2 | 2 | 2 | |
| | **Cambodia** | 1 | | | | 1 | 1 | |
| | **KSA** | | | | 1 | 1 | 1 | |
| | **Malaysia** | 1 | | 1 | | 1 | 1 | |
| | **Oman** | | 1 | | | 1 | 1 | |
| | **UAE** | | | | 1 | 1 | 1 | |
| **Europe** | **Italy** | 2 | 2 | | 58 | 62 | 62 | |
| | **Portugal** | | 1 | | 25 | 26 | 26 | |
| | **Spain** | 15 | | | 3 | 18 | 18 | |
| | **UK** | 12 | | | | 12 | 12 | |
| | **Switzerland** | 9 | | | | 9 | 9 | |
| | **Netherland** | 8 | | | | 8 | 8 | |
| | **Denmark** | 4 | | | | 4 | 4 | |
| | **France** | 1 | | | | 1 | 1 | |
| | **Belgium** | | | | 1 | 1 | 1 | |
| | **Norway** | | | | 1 | 1 | 1 | |
| | **Austria** | 1 | | | | 1 | 1 | |
| | **Finland** | 1 | | | | 1 | 1 | |
| | **Poland** | | | 1 | | 1 | 1 | |
| | **Hungary** | | 1 | | | 1 | 1 | |
| **Africa** | **South Africa** | | 4 | 14 | 8 | 26 | 26 | |
| | **Egypt** | | | | 1 | 1 | 1 | |
| | **Algeria** | | | | 1 | 1 | 1 | |
| **South America** | **Colombia** | | | | 12 | 12 | 12 | |
| | **Argentina** | | | | 8 | 8 | 8 | |
| | **Brazil** | | | | 3 | 3 | 3 | |
| | **Venezuela** | 1 | | | | 1 | 1 | |
| | **Ecuador** | | | | 1 | 1 | 1 | |
| **North America** | **U. S** | 4 | 1 | 2 | | 7 | 7 | |
| | **Canada** | | | | 2 | 2 | 2 | |
| **Oceania** | **Australia** | | | | 2 | 2 | 2 | |
| **Total** | | 1605 (77%) | 42(2%) | 28(1.3%) | 392(19.7%) | 2067 | 1286(62%) | 781(38%) |

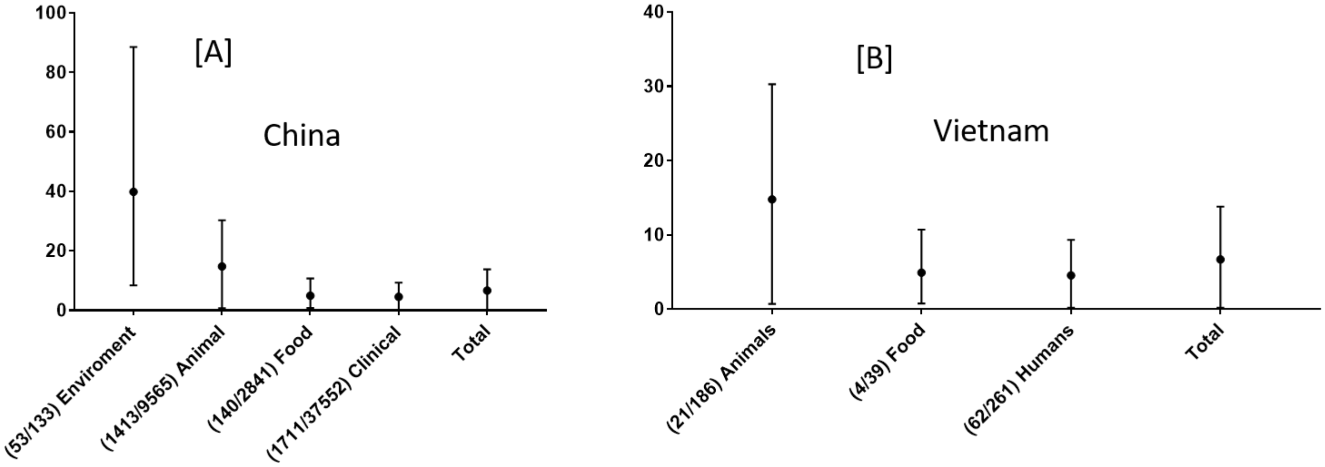


Supplemental figure 1. Prevalence of *mcr* genes sources in China and Vietnam

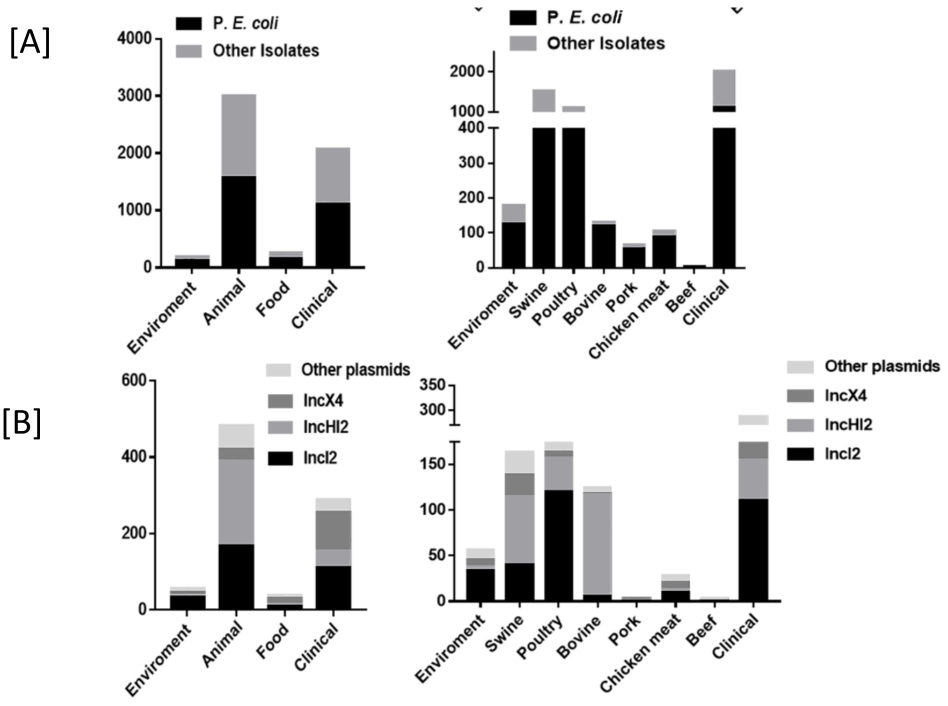


Supplemental figure 2. harboring bacteria and carrying plasmids of *mcr* genes.

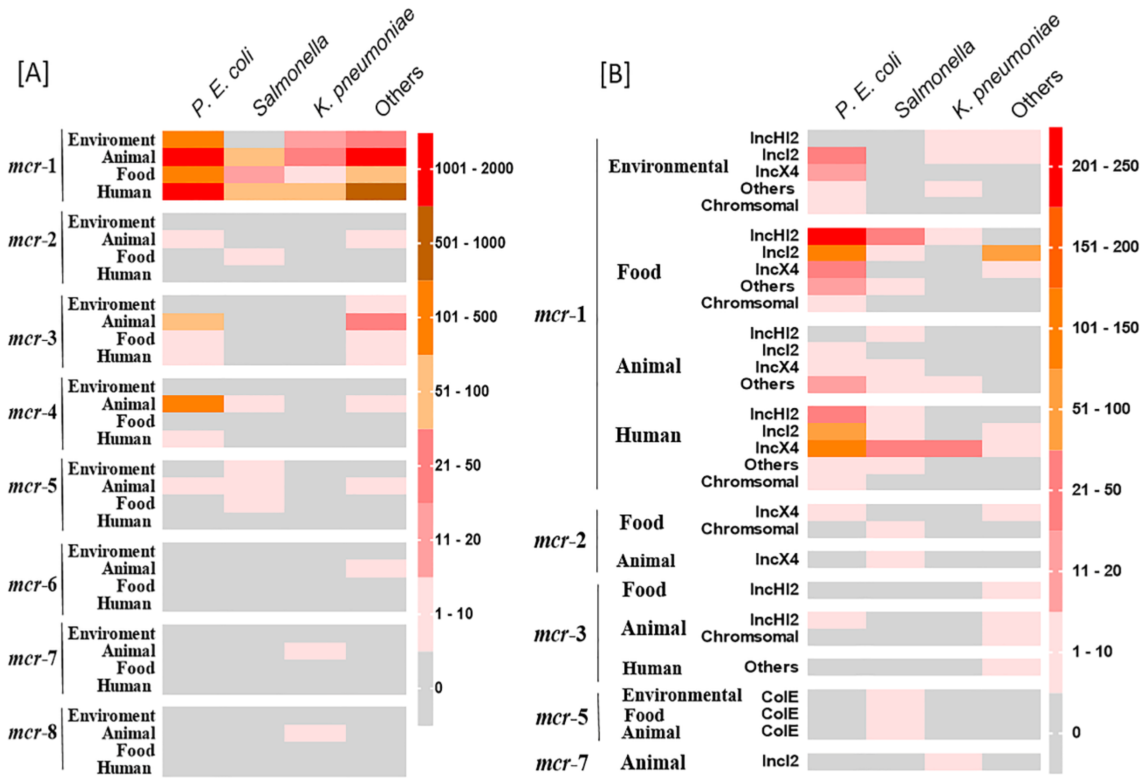


Supplemental figure 3. The frequency and distribution of various types of mcr genes.

N.B: Grey cells are blank.

## Supplemental references:

1. Fernandes MR, Sellera FP, Esposito F, Sabino CP, Cerdeira L, Lincopan N. Colistin-Resistant mcr-1-Positive Escherichia coli on Public Beaches, an Infectious Threat Emerging in Recreational Waters. 2017; **61**(7).
2. Zhao F, Feng Y, Lu X, McNally A, Zong Z. Remarkable Diversity of Escherichia coli Carrying mcr-1 from Hospital Sewage with the Identification of Two New mcr-1 Variants. *Frontiers in microbiology* 2017; **8**: 2094.
3. Caltagirone M, Nucleo E, Spalla M, et al. Occurrence of Extended Spectrum β-Lactamases, KPC-Type, and MCR-1.2-Producing Enterobacteriaceae from Wells, River Water, and Wastewater Treatment Plants in Oltrepò Pavese Area, Northern Italy. *Frontiers in microbiology* 2017; **8**(2232).
4. Ovejero CM, Delgado-Blas JF, Calero-Caceres W, Muniesa M, Gonzalez-Zorn B. Spread of mcr-1-carrying Enterobacteriaceae in sewage water from Spain. *The Journal of antimicrobial chemotherapy* 2017; **72**(4): 1050-3.
5. Borowiak M, Fischer J, Hammerl JA, Hendriksen RS, Szabo I, Malorny B. Identification of a novel transposon-associated phosphoethanolamine transferase gene, mcr-5, conferring colistin resistance in d-tartrate fermenting Salmonella enterica subsp. enterica serovar Paratyphi B. *The Journal of antimicrobial chemotherapy* 2017.
6. Zhao F, Feng Y, Lu X, McNally A, Zong Z. IncP Plasmid Carrying Colistin Resistance Gene mcr-1 in Klebsiella pneumoniae from Hospital Sewage. *Antimicrobial agents and chemotherapy* 2017; **61**(2).
7. Chen K, Chan EW, Xie M, Ye L, Dong N, Chen S. Widespread distribution of mcr-1-bearing bacteria in the ecosystem, 2015 to 2016. *Euro surveillance : bulletin Europeen sur les maladies transmissibles = European communicable disease bulletin* 2017; **22**(39).
8. Drali R, Berrazeg M, Zidouni LL, et al. Emergence of mcr-1 plasmid-mediated colistin-resistant Escherichia coli isolates from seawater. *The Science of the total environment* 2018; **642**: 90-4.
9. Zhao F, Zong Z. Kluyvera ascorbata Strain from Hospital Sewage Carrying the mcr-1 Colistin Resistance Gene. *Antimicrobial agents and chemotherapy* 2016; **60**(12): 7498-501.
10. Zhou HW, Zhang T, Ma JH, et al. Occurrence of Plasmid- and Chromosome-Carried mcr-1 in Waterborne Enterobacteriaceae in China. *Antimicrobial agents and chemotherapy* 2017; **61**(8).
11. Shen Y, Xu C, Sun Q, et al. Prevalence and Genetic Analysis of mcr-3-Positive Aeromonas Species from Humans, Retail Meat, and Environmental Water Samples. *Antimicrobial agents and chemotherapy* 2018; **62**(9).
12. Chen X, Zhao X, Che J, et al. Detection and dissemination of the colistin resistance gene, mcr-1, from isolates and faecal samples in China. *Journal of medical microbiology* 2017; **66**(2): 119-25.
13. Bai L, Hurley D, Li J, et al. Characterisation of multidrug-resistant Shiga toxin-producing Escherichia coli cultured from pigs in China: co-occurrence of extended-spectrum beta-lactamase- and mcr-1-encoding genes on plasmids. *International journal of antimicrobial agents* 2016; **48**(4): 445-8.
14. Zhang RM. The molecular epidemiology of The NDM and MCR-l positive E. Coli from poultry production chain: China Agricultural University, Beijing, China.; 2017.
15. Wu J, Huang Y, Rao D, Zhang Y, Yang K. Evidence for Environmental Dissemination of Antibiotic Resistance Mediated by Wild Birds. *Frontiers in microbiology* 2018; **9**: 745.
16. Lv L, Cao Y, Yu P, et al. Detection of mcr-1 Gene among Escherichia coli Isolates from Farmed Fish and Characterization of mcr-1-Bearing IncP Plasmids. *Antimicrobial agents and chemotherapy* 2018; **62**(3).
17. Jorgensen SB, Soraas A, Arnesen LS, Leegaard T, Sundsfjord A, Jenum PA. First environmental sample containing plasmid-mediated colistin-resistant ESBL-producing Escherichia coli detected in Norway. *APMIS : acta pathologica, microbiologica, et immunologica Scandinavica* 2017; **125**(9): 822-5.
18. Yu CY, Ang GY, Chin PS, Ngeow YF, Yin WF, Chan KG. Emergence of mcr-1-mediated colistin resistance in Escherichia coli in Malaysia. *International journal of antimicrobial agents* 2016; **47**(6): 504-5.

19. Zurfuh K, Poirel L, Nordmann P, Nuesch-Inderbinen M, Hachler H, Stephan R. Occurrence of the Plasmid-Borne mcr-1 Colistin Resistance Gene in Extended-Spectrum-beta-Lactamase-Producing Enterobacteriaceae in River Water and Imported Vegetable Samples in Switzerland. *Antimicrobial agents and chemotherapy* 2016; **60**(4): 2594-5.
20. Malhotra-Kumar S, Xavier BB, Das AJ, Lammens C, Butaye P, Goossens H. Colistin resistance gene mcr-1 harboured on a multidrug resistant plasmid. *The Lancet Infectious diseases* 2016; **16**(3): 283-4.
21. Xavier BB, Lammens C, Ruhal R, et al. Identification of a novel plasmid-mediated colistin-resistance gene, mcr-2, in Escherichia coli, Belgium, June 2016. *Euro surveillance : bulletin Europeen sur les maladies transmissibles = European communicable disease bulletin* 2016; **21**(27).
22. Carattoli A, Villa L, Feudi C, et al. Novel plasmid-mediated colistin resistance mcr-4 gene in Salmonella and Escherichia coli, Italy 2013, Spain and Belgium, 2015 to 2016. *Eurosurveillance* 2017; **22**(31): 30589.
23. Fernandes MR, Moura Q, Sartori L, et al. Silent dissemination of colistin-resistant Escherichia coli in South America could contribute to the global spread of the mcr-1 gene. *Euro surveillance : bulletin Europeen sur les maladies transmissibles = European communicable disease bulletin* 2016; **21**(17).
24. Li R, Xie M, Zhang J, et al. Genetic characterization of mcr-1-bearing plasmids to depict molecular mechanisms underlying dissemination of the colistin resistance determinant. *The Journal of antimicrobial chemotherapy* 2017; **72**(2): 393-401.
25. Kong LH, Lei CW, Ma SZ, et al. Various Sequence Types of Escherichia coli Isolates Coharboring blaNDM-5 and mcr-1 Genes from a Commercial Swine Farm in China. *Antimicrobial agents and chemotherapy* 2017; **61**(3).
26. Liu Y-Y, Wang Y, Walsh TR, et al. Emergence of plasmid-mediated colistin resistance mechanism MCR-1 in animals and human beings in China: a microbiological and molecular biological study. *The Lancet Infectious Diseases* 2016; **16**(2): 161-8.
27. Liu YY. Emergence of Plasmid-Mediated Colistin Resistance Gene mcr-1 in Enterobacteriacea. . China: South China Agricultural University, Guangzhou, China; 2016.
28. Yu LF. Transfer Mechanism of Colistin Resistance Gene mcr-1 among Escherichia coli Isolates from a Livestock Farm of Shanghai. Guangzhou, China: South China Agricultural University; 2016.
29. Liu Z. Virulence and resistance analysis of the Escherichia coli isolated from diarrhea piglets. . Changchun, China: Jilin University 2017.
30. Tong H, Liu J, Yao X, et al. High carriage rate of mcr-1 and antimicrobial resistance profiles of mcr-1-positive Escherichia coli isolates in swine faecal samples collected from eighteen provinces in China. *Veterinary Microbiology* 2018; **225**: 53-7.
31. Liu B-T, Song F-J, Zou M. Characterization of Highly Prevalent Plasmids Coharboring mcr-1, oqxAB, and blaCTX-M and Plasmids Harboring oqxAB and blaCTX-M in Escherichia coli Isolates from Food-Producing Animals in China. *Microbial Drug Resistance* 2018.
32. Lai CC, Lin YT, Lin YT, et al. Clinical characteristics of patients with bacteraemia due to the emergence of mcr-1-harbouring Enterobacteriaceae in humans and pigs in Taiwan. *International journal of antimicrobial agents* 2018.
33. Sun J, Li XP, Fang LX, et al. Co-occurrence of mcr-1 in the chromosome and on an IncHI2 plasmid: persistence of colistin resistance in Escherichia coli. *International journal of antimicrobial agents* 2018; **51**(6): 842-7.
34. Yin W, Li H, Shen Y, et al. Novel Plasmid-Mediated Colistin Resistance Gene mcr-3 in Escherichia coli. *mBio* 2017; **8**(3).
35. HAN Yixiao ZW, LIANG Bing, FENG Shuzhang, SUN Yang, LIUJun, ZHU Lingwei, JI Xue, GUO Xuejun. Isolation and identification of Escherichia coli with resistance to colistin from swine of different growth stages. *ChinJ Vet Sci* 2018; **38**(3): 481-5.

36. Brauer A, Telling K, Laht M, et al. Plasmid with Colistin Resistance Gene mcr-1 in Extended-Spectrum-beta-Lactamase-Producing Escherichia coli Strains Isolated from Pig Slurry in Estonia. *Antimicrobial agents and chemotherapy* 2016; **60**(11): 6933-6.
37. Falgenhauer L, Waezsada SE, Yao Y, et al. Colistin resistance gene mcr-1 in extended-spectrum beta-lactamase-producing and carbapenemase-producing Gram-negative bacteria in Germany. *The Lancet Infectious diseases* 2016; **16**(3): 282-3.
38. Olaitan AO, Chabou S, Okdah L, Morand S, Rolain JM. Dissemination of the mcr-1 colistin resistance gene. *The Lancet Infectious diseases* 2016; **16**(2): 147.
39. Kieffer N, Aires-de-Sousa M, Nordmann P, Poirel L. High Rate of MCR-1-Producing Escherichia coli and Klebsiella pneumoniae among Pigs, Portugal. *Emerg Infect Dis* 2017; **23**(12): 2023-9.
40. Belaynehe KM, Shin SW, Park KY, et al. Emergence of mcr-1 and mcr-3 variants coding for plasmid-mediated colistin resistance in Escherichia coli isolates from food- producing animals in South Korea. *International journal of infectious diseases : IJID : official publication of the International Society for Infectious Diseases* 2018; **72**: 22-4.
41. Quesada A, Ugarte-Ruiz M, Iglesias MR, et al. Detection of plasmid mediated colistin resistance (MCR-1) in Escherichia coli and Salmonella enterica isolated from poultry and swine in Spain. *Research in veterinary science* 2016; **105**: 134-5.
42. Garcia V, Garcia-Menino I, Mora A, et al. Co-occurrence of mcr-1, mcr-4 and mcr-5 genes in multidrug-resistant ST10 Enterotoxigenic and Shiga toxin-producing Escherichia coli in Spain (2006-2017). *International journal of antimicrobial agents* 2018; **52**(1): 104-8.
43. Anjum MF, Duggett NA, AbuOun M, et al. Colistin resistance in Salmonella and Escherichia coli isolates from a pig farm in Great Britain. *The Journal of antimicrobial chemotherapy* 2016; **71**(8): 2306-13.
44. Randall LP, Horton RA, Lemma F, et al. Longitudinal study on the occurrence in pigs of colistin-resistant Escherichia coli carrying mcr-1 following the cessation of use of colistin. *Journal of applied microbiology* 2018; **125**(2): 596-608.
45. Meinersmann RJ, Ladely SR, Plumblee JR, Cook KL, Thacker E. Prevalence of mcr-1 in the Cecal Contents of Food Animals in the United States. *Antimicrobial agents and chemotherapy* 2017; **61**(2).
46. Delgado-Blas JF, Ovejero CM, Abadia-Patino L, Gonzalez-Zorn B. Coexistence of mcr-1 and blaNDM-1 in Escherichia coli from Venezuela. *Antimicrobial agents and chemotherapy* 2016; **60**(10): 6356-8.
47. Malhotra-Kumar S, Xavier BB, Das AJ, et al. Colistin-resistant Escherichia coli harbouring mcr-1 isolated from food animals in Hanoi, Vietnam. *The Lancet Infectious Diseases* 2016; **16**(3): 286-7.
48. Cui MQ. Study on the transmission mechanism of mcr-1 gene in Salmonella and the microarray assay for the detection of foodborne pathogens, their resistance and virulence genes. Beijing, China Agricultural University; 2017.
49. Wang X, Wang Y, Zhou Y, et al. Emergence of a novel mobile colistin resistance gene, mcr-8, in NDM-producing Klebsiella pneumoniae. *Emerging microbes & infections* 2018; **7**(1): 122.
50. Sun JN. Study on the Drug resistance of Streptococcus in the Main Links of Pig Industry Chain in Western Liaoning province and Its transmission. Jinzhou, China.: Jinzhou Medical University; 2017.
51. Ma S, Sun C, Hulth A, et al. Mobile colistin resistance gene mcr-5 in porcine Aeromonas hydrophila. *The Journal of antimicrobial chemotherapy* 2018.
52. Li J, Hulth A, Nilsson LE, et al. Occurrence of the mobile colistin resistance gene mcr-3 in Escherichia coli from household pigs in rural areas. *The Journal of antimicrobial chemotherapy* 2018; **73**(6): 1721-3.
53. Xu Y, Zhong LL, Srinivas S, et al. Spread of MCR-3 Colistin Resistance in China: An Epidemiological, Genomic and Mechanistic Study. *EBioMedicine* 2018.

54. AbuOun M, Stubberfield EJ, Duggett NA, et al. mcr-1 and mcr-2 (mcr-6.1) variant genes identified in Moraxella species isolated from pigs in Great Britain from 2014 to 2015. *Journal of Antimicrobial Chemotherapy* 2017; **72**(10): 2745-9.
55. Duggett NA, Randall LP, Horton RA, et al. Molecular epidemiology of isolates with multiple mcr plasmids from a pig farm in Great Britain: the effects of colistin withdrawal in the short and long term. *The Journal of antimicrobial chemotherapy* 2018; **73**(11): 3025-33.
56. Kawanishi M, Abo H, Ozawa M, et al. Prevalence of Colistin Resistance Gene mcr-1 and Absence of mcr-2 in Escherichia coli Isolated from Healthy Food-Producing Animals in Japan. *Antimicrobial agents and chemotherapy* 2017; **61**(1).
57. Perrin-Guyomard A, Bruneau M, Houee P, et al. Prevalence of mcr-1 in commensal Escherichia coli from French livestock, 2007 to 2014. *Euro surveillance : bulletin Europeen sur les maladies transmissibles = European communicable disease bulletin* 2016; **21**(6).
58. El Garch F, de Jong A, Bertrand X, Hocquet D, Sauget M. mcr-1- like detection in commensal Escherichia coli and Salmonella spp. from food-producing animals at slaughter in Europe. *Veterinary Microbiology* 2018; **213**: 42-6.
59. Dandachi I, Fayad E, El-Bazzal B, Daoud Z, Rolain JM. Prevalence of Extended-Spectrum Beta-Lactamase-Producing Gram-Negative Bacilli and Emergence of mcr-1 Colistin Resistance Gene in Lebanese Swine Farms. *Microbial drug resistance (Larchmont, NY)* 2018.
60. Hille K, Roschanski N, Ruddat I, et al. Investigation of potential risk factors for the occurrence of Escherichia coli isolates from German fattening pig farms harbouring the mcr-1 colistin-resistance gene. *International journal of antimicrobial agents* 2018; **51**(2): 177-80.
61. Poirel L, Kieffer N, Fernandez-Garayzabal JF, Vela AI, Larpin Y, Nordmann P. MCR-2-mediated plasmid-borne polymyxin resistance most likely originates from Moraxella pluranimalium. *The Journal of antimicrobial chemotherapy* 2017; **72**(10): 2947-9.
62. Bachiri T, Lalaoui R, Bakour S, et al. First Report of the Plasmid-Mediated Colistin Resistance Gene mcr-1 in Escherichia coli ST405 Isolated from Wildlife in Bejaia, Algeria. *Microbial drug resistance (Larchmont, NY)* 2018; **24**(7): 890-5.
63. Dominguez JE, Redondo LM, Figueroa Espinosa RA, et al. Simultaneous Carriage of mcr-1 and Other Antimicrobial Resistance Determinants in Escherichia coli From Poultry. *Frontiers in microbiology* 2018; **9**: 1679.
64. Lima Barbieri N, Nielsen DW, Wannemuehler Y, et al. mcr-1 identified in Avian Pathogenic Escherichia coli (APEC). *PloS one* 2017; **12**(3): e0172997.
65. Liu BT, Song FJ, Zou M, Zhang QD, Shan H. High Incidence of Escherichia coli Strains Coharboring mcr-1 and blaNDM from Chickens. *Antimicrobial agents and chemotherapy* 2017; **61**(3).
66. Shen Z, Wang Y, Shen Y, Shen J, Wu C. Early emergence of mcr-1 in Escherichia coli from food-producing animals. *The Lancet Infectious Diseases* 2016; **16**(3): 293.
67. Wang X, Wang Y, Wang Y, Zhang S, Shen Z, Wang S. Emergence of the colistin resistance gene mcr-1 and its variant in several uncommon species of Enterobacteriaceae from commercial poultry farm surrounding environments. *Vet Microbiol* 2018; **219**: 161-4.
68. Wu C, Wang Y, Shi X, et al. Rapid rise of the ESBL and mcr-1 genes in Escherichia coli of chicken origin in China, 2008-2014. *Emerging microbes & infections* 2018; **7**(1): 30.
69. Alba P, Leekitcharoenphon P, Franco A, et al. Molecular Epidemiology of mcr-Encoded Colistin Resistance in Enterobacteriaceae From Food-Producing Animals in Italy Revealed Through the EU Harmonized Antimicrobial Resistance Monitoring. *Frontiers in microbiology* 2018; **9**: 1217.
70. Xiang R, Liu BH, Zhang AY, et al. Colocation of the Polymyxin Resistance Gene mcr-1 and a Variant of mcr-3 on a Plasmid in an Escherichia coli Isolate from a Chicken Farm. *Antimicrobial agents and chemotherapy* 2018; **62**(6).

71. Moawad AA, Hotzel H, Neubauer H, et al. Antimicrobial resistance in Enterobacteriaceae from healthy broilers in Egypt: emergence of colistin-resistant and extended-spectrum beta-lactamase-producing Escherichia coli. *Gut pathogens* 2018; **10**: 39.
72. Magistrali CF, Curcio L, Luppi A, et al. Mobile colistin resistance genes in Escherichia coli from pigs affected by colibacillosis. *International journal of antimicrobial agents* 2018; **52**(5): 744-6.
73. Azam M, Ehsan I, Sajjad Ur R, Saleemi MK, Javed MR, Mohsin M. Detection of the colistin resistance gene mcr-1 in avian pathogenic Escherichia coli in Pakistan. *Journal of global antimicrobial resistance* 2017; **11**: 152-3.
74. Manageiro V, Clemente L, Graca R, et al. New insights into resistance to colistin and third-generation cephalosporins of Escherichia coli in poultry, Portugal: Novel blaCTX-M-166 and blaESAC genes. *International journal of food microbiology* 2017; **263**: 67-73.
75. Grami R, Mansour W, Mehri W, et al. Impact of food animal trade on the spread of mcr-1-mediated colistin resistance, Tunisia, July 2015. *Euro surveillance : bulletin Europeen sur les maladies transmissibles = European communicable disease bulletin* 2016; **21**(8): 30144.
76. Maamar E, Alonso CA, Hamzaoui Z, et al. Emergence of plasmid-mediated colistin-resistance in CMY-2-producing Escherichia coli of lineage ST2197 in a Tunisian poultry farm. *International journal of food microbiology* 2018; **269**: 60-3.
77. Bui TKN, Bui TMH, Ueda S, Le DT, Yamamoto Y, Hirai I. Potential transmission opportunity of CTX-M-producing Escherichia coli on a large-scale chicken farm in Vietnam. *Journal of global antimicrobial resistance* 2018; **13**: 1-6.
78. Xiang R, Ye X, Tuo H, et al. Co-occurrence of mcr-3 and blaNDM-5 genes in multidrug-resistant Klebsiella pneumoniae ST709 from a commercial chicken farm in China. *International journal of antimicrobial agents* 2018.
79. Yang YQ, Li YX, Lei CW, Zhang AY, Wang HN. Novel plasmid-mediated colistin resistance gene mcr-7.1 in Klebsiella pneumoniae. *The Journal of antimicrobial chemotherapy* 2018.
80. Wang J, Li X, Li J, et al. Complete genetic analysis of a Salmonella enterica serovar Indiana isolate accompanying four plasmids carrying mcr-1, ESBL and other resistance genes in China. *Vet Microbiol* 2017; **210**: 142-6.
81. Wang Y. The analysis of antimicrobial resistance and the plasmid mediated resistant genes in Salmonella Typhimurium isolates: Jilin Agricultural University , Changchun, China; 2016.
82. Webb HE, Granier SA, Marault M, et al. Dissemination of the mcr-1 colistin resistance gene. *The Lancet Infectious Diseases* 2016; **16**(2): 144-5.
83. Carfora V, Alba P, Leekitcharoenphon P, et al. Colistin Resistance Mediated by mcr-1 in ESBL-Producing, Multidrug Resistant Salmonella Infantis in Broiler Chicken Industry, Italy (2016-2017). *Frontiers in microbiology* 2018; **9**: 1880.
84. Lentz SA, de Lima-Morales D, Cuppertino VM, et al. Letter to the editor: Escherichia coli harbouring mcr-1 gene isolated from poultry not exposed to polymyxins in Brazil. *Euro surveillance : bulletin Europeen sur les maladies transmissibles = European communicable disease bulletin* 2016; **21**(26).
85. Liu J, Yang Y, Li Y, et al. Isolation of an IncP-1 plasmid harbouring mcr-1 from a chicken isolate of Citrobacter braakii in China. *International journal of antimicrobial agents* 2018; **51**(6): 936-40.
86. Liu BT, Song FJ, Zou M, Hao ZH, Shan H. Emergence of Colistin Resistance Gene mcr-1 in Cronobacter sakazakii Producing NDM-9 and in Escherichia coli from the Same Animal. *Antimicrobial agents and chemotherapy* 2017; **61**(2).
87. Liakopoulos A, Mevius DJ, Olsen B, Bonnedahl J. The colistin resistance mcr-1 gene is going wild. *The Journal of antimicrobial chemotherapy* 2016; **71**(8): 2335-6.
88. Hmede Z, Kassem, II. The Colistin Resistance Gene, mcr-1, is Prevalent in Commensal E. coli Isolated from Lebanese Pre-harvest Poultry. *Antimicrobial agents and chemotherapy* 2018.

89. Lv J, Mohsin M, Lei S, et al. Discovery of a mcr-1-bearing plasmid in commensal colistin-resistant Escherichia coli from healthy broilers in Faisalabad, Pakistan. *Virulence* 2018; **9**(1): 994-9.
90. Khalifa HO, Ahmed AM, Oreiby AF, Eid AM, Shimamoto T, Shimamoto T. Characterisation of the plasmid-mediated colistin resistance gene mcr-1 in Escherichia coli isolated from animals in Egypt. *International journal of antimicrobial agents* 2016; **47**(5): 413-4.
91. Haenni M, Poirel L, Kieffer N, et al. Co-occurrence of extended spectrum beta lactamase and MCR-1 encoding genes on plasmids. *The Lancet Infectious diseases* 2016; **16**(3): 281-2.
92. Hernandez M, Iglesias MR, Rodriguez-Lazaro D, et al. Co-occurrence of colistin-resistance genes mcr-1 and mcr-3 among multidrug-resistant Escherichia coli isolated from cattle, Spain, September 2015. *Euro surveillance : bulletin Europeen sur les maladies transmissibles = European communicable disease bulletin* 2017; **22**(31).
93. Lupo A, Saras E, Madec JY, Haenni M. Emergence of blaCTX-M-55 associated with fosA, rmtB and mcr gene variants in Escherichia coli from various animal species in France. *The Journal of antimicrobial chemotherapy* 2018; **73**(4): 867-72.
94. Eichhorn I, Feudi C, Wang Y, et al. Identification of novel variants of the colistin resistance gene mcr-3 in Aeromonas spp. from the national resistance monitoring programme GERM-Vet and from diagnostic submissions. *The Journal of antimicrobial chemotherapy* 2018; **73**(5): 1217-21.
95. Ruzauskas M, Vaskeviciute L. Detection of the mcr-1 gene in Escherichia coli prevalent in the migratory bird species Larus argentatus. *The Journal of antimicrobial chemotherapy* 2016; **71**(8): 2333-4.
96. Unger F, Eisenberg T, Prenger-Berninghoff E, et al. Imported reptiles as a risk factor for the global distribution of Escherichia coli harbouring the colistin resistance gene mcr-1. *International journal of antimicrobial agents* 2017; **49**(1): 122-3.
97. Wang R, Liu Y, Zhang Q, et al. The prevalence of colistin resistance in Escherichia coli and Klebsiella pneumoniae isolated from food animals in China: coexistence of mcr-1 and blaNDM with low fitness cost. *International journal of antimicrobial agents* 2018; **51**(5): 739-44.
98. Kuo SC, Huang WC, Wang HY, Shiau YR, Cheng MF, Lauderdale TL. Colistin resistance gene mcr-1 in Escherichia coli isolates from humans and retail meats, Taiwan. *The Journal of antimicrobial chemotherapy* 2016; **71**(8): 2327-9.
99. Nishino Y, Shimojima Y, Suzuki Y, et al. Detection of the mcr-1 gene in colistin resistant Escherichia coli from retail meat in Japan. *Microbiology and immunology* 2017.
100. Garcia-Graells C, De Keersmaecker SCJ, Vanneste K, et al. Detection of Plasmid-Mediated Colistin Resistance, mcr-1 and mcr-2 Genes, in Salmonella spp. Isolated from Food at Retail in Belgium from 2012 to 2015. *Foodborne pathogens and disease* 2017.
101. Yao X, Doi Y, Zeng L, Lv L, Liu JH. Carbapenem-resistant and colistin-resistant Escherichia coli co-producing NDM-9 and MCR-1. *The Lancet Infectious diseases* 2016; **16**(3): 288-9.
102. Ghafur A, Shankar C, GnanaSoundari P, et al. Detection of chromosomal and plasmid mediated mechanisms conferring colistin resistance in Escherichia coli and Klebsiella pneumoniae from Indian food samples. *Journal of global antimicrobial resistance* 2018.
103. Kluytmans-van den Bergh MF, Huizinga P, Bonten MJ, et al. Presence of mcr-1-positive Enterobacteriaceae in retail chicken meat but not in humans in the Netherlands since 2009. *Euro surveillance : bulletin Europeen sur les maladies transmissibles = European communicable disease bulletin* 2016; **21**(9): 30149.
104. Dona V, Bernasconi OJ, Pires J, et al. Heterogeneous Genetic Location of mcr-1 in Colistin-Resistant Escherichia coli Isolates from Humans and Retail Chicken Meat in Switzerland: Emergence of mcr-1-Carrying IncK2 Plasmids. 2017; **61**(11).
105. Kurekci C, Aydin M, Nalbantoglu OU, Gundogdu A. First report of Escherichia coli carrying the mobile colistin resistance gene mcr-1 in Turkey. *Journal of global antimicrobial resistance* 2018; **15**: 169-70.

106. Doumith M, Godbole G, Ashton P, et al. Detection of the plasmid-mediated mcr-1 gene conferring colistin resistance in human and food isolates of Salmonella enterica and Escherichia coli in England and Wales. *The Journal of antimicrobial chemotherapy* 2016; **71**(8): 2300-5.
107. Ling Z, Yin W, Li H, et al. Chromosome-Mediated mcr-3 Variants in Aeromonas veronii from Chicken Meat. *Antimicrobial agents and chemotherapy* 2017; **61**(11).
108. Mulvey MR, Mataseje LF, Robertson J, et al. Dissemination of the mcr-1 colistin resistance gene. *The Lancet Infectious diseases* 2016; **16**(3): 289-90.
109. Yao X. Molecular epidemiology of ESBLs- and Carbapenemases-producing Enterobacteriaceae from retail meat and vegetables. . Guangzhou, China: South China Agricultural University 2016.
110. Luo J, Yao X, Lv L, et al. Emergence of mcr-1 in Raoultella ornithinolytica and Escherichia coli Isolates from Retail Vegetables in China. *Antimicrobial agents and chemotherapy* 2017; **61**(10).
111. Saavedra SY, Diaz L, Wiesner M, et al. Genomic and Molecular Characterization of Clinical Isolates of Enterobacteriaceae Harboring mcr-1 in Colombia, 2002-2016. *Antimicrobial agents and chemotherapy* 2017.
112. Yamaguchi T, Kawahara R, Harada K, et al. The presence of colistin resistance gene mcr-1 and -3 in ESBL producing Escherichia coli isolated from food in Ho Chi Minh City, Vietnam. *FEMS microbiology letters* 2018; **365**(11).
113. HU Yujie LC, WANG Meimei, GAN Xin, XU Jin, LI Fengqin,YANG Dajin, PEI Xiaoyan. Resistance Characteristic Analaysis for Foodborne Salmonella Isolates from China, 26 Provinence Study. *CHINESE JOURNAL OF FOOD HYGIENE* 2018; **30**(5): 456-61.
114. HU Yujie WW, YAN Shaofei , GAN Xin , LYU Hanyang , LIU Chang, YANG Da-jin, LI Feng-qin, XU Jin. Resistance Analysis of 1070 Salmonella strains isolated from food sample in mainland China. *CHINESE JOURNAL OF FOOD HYGIENE* 2017; **26**(6): 647- 52.
115. Tse H, Yuen K-Y. Dissemination of the mcr-1 colistin resistance gene. *The Lancet Infectious Diseases* 2016; **16**(2): 145-6.
116. Sennati S, Di Pilato V, Riccobono E, et al. Citrobacter braakii carrying plasmid-borne mcr-1 colistin resistance gene from ready-to-eat food from a market in the Chaco region of Bolivia. *Journal of Antimicrobial Chemotherapy* 2017; **72**(7): 2127-9.
117. Hartl R, Kerschner H, Lepuschitz S, Ruppitsch W, Allerberger F, Apfalter P. Detection of the mcr-1 Gene in a Multidrug-Resistant Escherichia coli Isolate from an Austrian Patient. *Antimicrobial agents and chemotherapy* 2017; **61**(4).
118. Stoesser N, Mathers AJ, Moore CE, Day NP, Crook DW. Colistin resistance gene mcr-1 and pHNSHP45 plasmid in human isolates of Escherichia coli and Klebsiella pneumoniae. *The Lancet Infectious diseases* 2016; **16**(3): 285-6.
119. Hu YY, Wang YL, Sun QL, et al. Colistin resistance gene mcr-1 in gut flora of children. *International journal of antimicrobial agents* 2017; **50**(4): 593-7.
120. Bi Z, Berglund B, Sun Q, et al. Prevalence of the mcr-1 colistin resistance gene in extended-spectrum beta-lactamase-producing Escherichia coli from human faecal samples collected in 2012 in rural villages in Shandong Province, China. *International journal of antimicrobial agents* 2017; **49**(4): 493-7.
121. Ye H, Li Y, Li Z, et al. Diversified mcr-1-Harbouring Plasmid Reservoirs Confer Resistance to Colistin in Human Gut Microbiota. *mBio* 2016; **7**(2): e00177.
122. Ruppe E, Le Chatelier E, Pons N, Andremont A, Ehrlich SD. Dissemination of the mcr-1 colistin resistance gene. *The Lancet Infectious diseases* 2016; **16**(3): 290-1.
123. Zhong LL, Phan HTT, Shen C, et al. High Rates of Human Fecal Carriage of mcr-1-Positive Multidrug-Resistant Enterobacteriaceae Emerge in China in Association With Successful Plasmid Families. *Clinical infectious diseases : an official publication of the Infectious Diseases Society of America* 2018; **66**(5): 676-85.

124. Chan WS, Au CH, Ho DN, Chan TL, Ma ES, Tang BS. Prospective study on human fecal carriage of Enterobacteriaceae possessing mcr-1 and mcr-2 genes in a regional hospital in Hong Kong. *BMC infectious diseases* 2018; **18**(1): 81.

125. Liu H, Zhu B, Liang B, et al. A Novel mcr-1 Variant Carried by an IncI2-Type Plasmid Identified From a Multidrug Resistant Enterotoxigenic Escherichia coli. *Frontiers in microbiology* 2018; **9**: 815.

126. Wang Y, Tian GB, Zhang R, et al. Prevalence, risk factors, outcomes, and molecular epidemiology of mcr-1-positive Enterobacteriaceae in patients and healthy adults from China: an epidemiological and clinical study. *The Lancet Infectious diseases* 2017; **17**(4): 390-9.

127. Kusumoto M, Ogura Y, Gotoh Y, Iwata T, Hayashi T, Akiba M. Colistin-Resistant mcr-1–Positive Pathogenic Escherichia coli in Swine, Japan, 2007−2014. *Emerging Infectious Diseases* 2016; **22**(7): 1315-7.

128. Tada T, Uechi K, Nakasone I, et al. Emergence of a colistin-resistant Escherichia coli clinical isolate harboring mcr-1 in Japan. *International journal of infectious diseases : IJID : official publication of the International Society for Infectious Diseases* 2017; **63**: 21-2.

129. Tada T, Uechi K, Nakasone I, et al. Emergence of IncX4 plasmids encoding mcr-1 in a clinical isolate of Klebsiella pneumoniae in Japan. *International journal of infectious diseases : IJID : official publication of the International Society for Infectious Diseases* 2018.

130. Uchida H, Tada T, Sugahara Y, Kato A, Miyairi I, Kirikae T. A clinical isolate of Escherichia coli co-harbouring mcr-1 and blaNDM-5 in Japan. *Journal of medical microbiology* 2018; **67**(8): 1047-9.

131. Grondahl-Yli-Hannuksela K, Lonnqvist E, Kallonen T, et al. The first human report of mobile colistin resistance gene, mcr-1, in Finland. *APMIS : acta pathologica, microbiologica, et immunologica Scandinavica* 2018; **126**(5): 413-7.

132. Terveer EM, Nijhuis RHT, Crobach MJT, et al. Prevalence of colistin resistance gene (mcr-1) containing Enterobacteriaceae in feces of patients attending a tertiary care hospital and detection of a mcr-1 containing, colistin susceptible E. coli. *PloS one* 2017; **12**(6): e0178598.

133. von Wintersdorff CJ, Wolffs PF, van Niekerk JM, et al. Detection of the plasmid-mediated colistin-resistance gene mcr-1 in faecal metagenomes of Dutch travellers. *The Journal of antimicrobial chemotherapy* 2016; **71**(12): 3416-9.

134. Kim ES, Chong YP, Park SJ, et al. Detection and genetic features of MCR-1-producing plasmid in human Escherichia coli infection in South Korea. *Diagnostic microbiology and infectious disease* 2017; **89**(2): 158-60.

135. Prim N, Rivera A, Rodriguez-Navarro J, et al. Detection of mcr-1 colistin resistance gene in polyclonal Escherichia coli isolates in Barcelona, Spain, 2012 to 2015. *Euro surveillance : bulletin Europeen sur les maladies transmissibles = European communicable disease bulletin* 2016; **21**(13).

136. Vasquez AM, Montero N, Laughlin M, et al. Investigation of Escherichia coli Harboring the mcr-1 Resistance Gene - Connecticut, 2016. *MMWR Morbidity and mortality weekly report* 2016; **65**(36): 979-80.

137. Macesic N, Green D, Wang Z, et al. Detection of mcr-1-Carrying Escherichia coli Causing Bloodstream Infection in a New York City Hospital: Avian Origins, Human Concerns? *Open forum infectious diseases* 2017; **4**(3): ofx115.

138. Dona V, Bernasconi OJ, Kasraian S, Tinguely R, Endimiani A. A SYBR(R) Green-based real-time PCR method for improved detection of mcr-1-mediated colistin resistance in human stool samples. *Journal of global antimicrobial resistance* 2017; **9**: 57-60.

139. Bernasconi OJ, Kuenzli E, Pires J, et al. Travelers Can Import Colistin-Resistant Enterobacteriaceae, Including Those Possessing the Plasmid-Mediated mcr-1 Gene. 2016; **60**(8): 5080-4.

140. Chen XW. The analysis of antimicrobial resistance and the plasmid mediated resistant genes in Salmonella Typhimurium isolates: Yangzhou University, · Yangzhou, China; 2017.

141. Lu X, Hu Y, Luo M, et al. MCR-1.6, a new MCR variant carried by an IncP plasmid in a colistin-resistant Salmonella enterica serovar Typhimurium isolate from a healthy individual. *Antimicrobial agents and chemotherapy* 2017; **61**(5).
142. Cao Y, Yu, X. J., Han, Y. Y., Li, J., Kan, B., Yan, M. Y. . Polymyxin resistance and mcr-1 prevalence in non-typhoid Salmonella isolates in China. . *DISEASE SURVEILLANCE* 2017; **32**(5): 365-71.
143. Di Pilato V, Arena F, Tascini C, et al. mcr-1.2, a New mcr Variant Carried on a Transferable Plasmid from a Colistin-Resistant KPC Carbapenemase-Producing Klebsiella pneumoniae Strain of Sequence Type 512. *Antimicrobial agents and chemotherapy* 2016; **60**(9): 5612-5.
144. Caspar Y, Maillet M, Pavese P, et al. mcr-1 Colistin Resistance in ESBL-Producing Klebsiella pneumoniae, France. *Emerg Infect Dis* 2017; **23**(5): 874-6.
145. Shenoy ES, Pierce VM, Walters MS, et al. Transmission of Mobile Colistin Resistance (mcr-1) by Duodenoscope. *Clinical infectious diseases : an official publication of the Infectious Diseases Society of America* 2018.
146. Ma Q, Huang Y, Wang J, et al. Multidrug-resistant Shigella sonnei carrying the plasmid-mediated mcr-1 gene in China. *International journal of antimicrobial agents* 2018; **52**(1): 14-21.
147. Shen Y, Zhou H, Xu J, et al. Anthropogenic and environmental factors associated with high incidence of mcr-1 carriage in humans across China. *Nature microbiology* 2018; **3**(9): 1054-62.
148. Pham Thanh D, Thanh Tuyen H, Nguyen Thi Nguyen T, et al. Inducible colistin resistance via a disrupted plasmid-borne mcr-1 gene in a 2008 Vietnamese Shigella sonnei isolate. *The Journal of antimicrobial chemotherapy* 2016; **71**(8): 2314-7.
149. Yanat B, Machuca J, Yahia RD, Touati A, Pascual A, Rodriguez-Martinez JM. First report of the plasmid-mediated colistin resistance gene mcr-1 in a clinical Escherichia coli isolate in Algeria. *International journal of antimicrobial agents* 2016; **48**(6): 760-1.
150. Ellem JA, Ginn AN, Chen SC, Ferguson J, Partridge SR, Iredell JR. Locally Acquired mcr-1 in Escherichia coli, Australia, 2011 and 2013. *Emerg Infect Dis* 2017; **23**(7): 1160-3.
151. Tijet N, Faccone D, Rapoport M, et al. Molecular characteristics of mcr-1-carrying plasmids and new mcr-1 variant recovered from polyclonal clinical Escherichia coli from Argentina and Canada. 2017; **12**(7): e0180347.
152. Huang TD, Bogaerts P, Berhin C, Hoebeke M, Bauraing C, Glupczynski Y. Increasing proportion of carbapenemase-producing Enterobacteriaceae and emergence of a MCR-1 producer through a multicentric study among hospital-based and private laboratories in Belgium from September to November 2015. *Euro surveillance : bulletin Europeen sur les maladies transmissibles = European communicable disease bulletin* 2017; **22**(19).
153. Fernandes MR, McCulloch JA, Vianello MA, et al. First Report of the Globally Disseminated IncX4 Plasmid Carrying the mcr-1 Gene in a Colistin-Resistant Escherichia coli Sequence Type 101 Isolate from a Human Infection in Brazil. 2016; **60**(10): 6415-7.
154. Sonnevend A, Ghazawi A, Alqahtani M, et al. Plasmid-mediated colistin resistance in Escherichia coli from the Arabian Peninsula. *International journal of infectious diseases : IJID : official publication of the International Society for Infectious Diseases* 2016; **50**: 85-90.
155. Quan J, Li X, Chen Y, et al. Prevalence of mcr-1 in Escherichia coli and Klebsiella pneumoniae recovered from bloodstream infections in China: a multicentre longitudinal study. *The Lancet Infectious diseases* 2017; **17**(4): 400-10.
156. Du H, Chen L, Tang YW, Kreiswirth BN. Emergence of the mcr-1 colistin resistance gene in carbapenem-resistant Enterobacteriaceae. *The Lancet Infectious diseases* 2016; **16**(3): 287-8.
157. Zhang R, Huang Y, Chan EW, Zhou H, Chen S. Dissemination of the mcr-1 colistin resistance gene. *The Lancet Infectious diseases* 2016; **16**(3): 291-2.
158. Yu H, Qu F, Shan B, et al. Detection of the mcr-1 Colistin Resistance Gene in Carbapenem-Resistant Enterobacteriaceae from Different Hospitals in China. 2016; **60**(8): 5033-5.

159. Wong SCY, Tse H, Chen JHK, Cheng VCC, Ho P-L, Yuen K-Y. Colistin-Resistant Enterobacteriaceae Carrying the mcr-1 Gene among Patients in Hong Kong. *Emerging infectious diseases* 2016; **22**(9): 1667-9.
160. Zhang X, Wang, Y. C., Sui, Y., Yin, J. Analysis of carbapenemases genes andcolistin resistance gene mcr-1 in carbapenem-resistant Enterobacteriaceae. . *Chin J Clin Infect Dis* 2016; **9**(5): 444-7.
161. Qi XM, Wang, X. J., Chen, H. B., Li, S. G., Zhao, C. J., Wang, Q., Wang, Z. W., ... Wang, H. Molecular mechanisms of colistin-resistant Klebsiella pneumoniae and Escherichia coli isolates. . *Chin J Lab Med* 2016; **39**(5): 618-24.
162. Zhou Y, Li G, Zhao S, et al. Detection and epidemiology of mcr-1-positive Escherichia coli strains in Dongyang area of Zhejiang Province. *Chinese Journal of Microbiology and Immunology* 2018; **38**(1): 41-6.
163. Li Y, Sun QL, Shen Y, et al. Rapid Increase in Prevalence of Carbapenem-Resistant Enterobacteriaceae (CRE) and Emergence of Colistin Resistance Gene mcr-1 in CRE in a Hospital in Henan, China. *Journal of clinical microbiology* 2018; **56**(4).
164. Feng S, Shen C, Chen H, et al. Co-production of MCR-1 and NDM-5 in Escherichia coli isolated from a colonization case of inpatient. *Infection and drug resistance* 2018; **11**: 1157-61.
165. Cao L, Li X, Xu Y, Shen J. Prevalence and molecular characteristics of mcr-1 colistin resistance in Escherichia coli: isolates of clinical infection from a Chinese University Hospital. *Infect Drug Resist* 2018; **11**: 1597-603.
166. Caselli E, D'Accolti M, Soffritti I, Piffanelli M, Mazzacane S. Spread of mcr-1-Driven Colistin Resistance on Hospital Surfaces, Italy. *Emerg Infect Dis* 2018; **24**(9): 1752-3.
167. Lu H, Wang C, Dong G, et al. Prevalence and Molecular Characterization of Escherichia coli Clinical Isolates Carrying mcr-1 in a Chinese Teaching Hospital from 2002 to 2016. *Antimicrobial agents and chemotherapy* 2018; **62**(9).
168. Sun Q, Hu Y, Zhou H, et al. Alkaline Peptone Water-Based Enrichment Method for mcr-3 From Acute Diarrheic Outpatient Gut Samples. *Frontiers in medicine* 2018; **5**: 99.
169. Wang QH, Liu, X. J., Hu, J. R., Chu, Y. Z. Screening for the mcr-1 Gene in the Members of the Enterobacteriaceae and Its Correlation with Colistin Resistant Phenotype. *Journal of China Medical University* 2018; **47**(8): 713-6.
170. Huang X, Sun, P. F., Xia, W. Y., Tang, C. J., Ni, F., Liu, G. Y. Study of a clinical extensive drug resistant Escherichia coli isolate coexistence of mcr-1 and NDM-6. *Chin J Clin Pharmacol* 2018; **34**(14): 1696-9.
171. Ortega-Paredes D, Barba P, Zurita J. Colistin-resistant Escherichia coli clinical isolate harbouring the mcr-1 gene in Ecuador. *Epidemiology and infection* 2016; **144**(14): 2967-70.
172. Elnahriry SS, Khalifa HO, Soliman AM, et al. Emergence of Plasmid-Mediated Colistin Resistance Gene mcr-1 in a Clinical Escherichia coli Isolate from Egypt. 2016; **60**(5): 3249-50.
173. Juhasz E, Ivan M, Pinter E, Pongracz J, Kristof K. Colistin resistance among blood culture isolates at a tertiary care centre in Hungary. *Journal of global antimicrobial resistance* 2017; **11**: 167-70.
174. Del Bianco F, Morotti M, Pedna MF, Farabegoli P, Sambri V. Microbiological surveillance of plasmid mediated colistin resistance in human Enterobacteriaceae isolates in Romagna (Northern Italy): August 2016-July 2017. *International journal of infectious diseases : IJID : official publication of the International Society for Infectious Diseases* 2018; **69**: 96-8.
175. Simoni S, Morroni G, Brenciani A, et al. Spread of colistin resistance gene mcr-1 in Italy: characterization of the mcr-1.2 allelic variant in a colistin-resistant blood isolate of Escherichia coli. *Diagnostic microbiology and infectious disease* 2018; **91**(1): 66-8.
176. Principe L, Piazza A, Mauri C, et al. Multicenter prospective study on the prevalence of colistin resistance in Escherichia coli: relevance of mcr-1-positive clinical isolates in Lombardy, Northern Italy. *Infect Drug Resist* 2018; **11**: 377-85.
177. Solheim M, Bohlin J, Ulstad CR, et al. Plasmid-mediated colistin-resistant Escherichia coli detected from 2014 in Norway. *International journal of antimicrobial agents* 2016; **48**(2): 227-8.

178. Mohsin J, Pal T, Petersen JE, et al. Plasmid-Mediated Colistin Resistance Gene mcr-1 in an Escherichia coli ST10 Bloodstream Isolate in the Sultanate of Oman. *Microbial drug resistance (Larchmont, NY)* 2017.
179. Izdebski R, Baraniak A, Bojarska K, et al. Mobile MCR-1-associated resistance to colistin in Poland. *The Journal of antimicrobial chemotherapy* 2016; **71**(8): 2331-3.
180. Poirel L, Aires-de-Sousa M, Kudyba P, Kieffer N, Nordmann P. Screening and Characterization of Multidrug-Resistant Gram-Negative Bacteria from a Remote African Area, Sao Tome and Principe. *Antimicrobial agents and chemotherapy* 2018; **62**(9).
181. Tacão M, Tavares RdS, Teixeira P, et al. mcr-1 and bla(KPC-3) in Escherichia coli Sequence Type 744 after Meropenem and Colistin Therapy, Portugal. *Emerging Infectious Diseases* 2017; **23**(8): 1419-21.
182. Sanchez-Benito R, Iglesias MR, Quijada NM, et al. Escherichia coli ST167 carrying plasmid mobilisable mcr-1 and blaCTX-M-15 resistance determinants isolated from a human respiratory infection. *International journal of antimicrobial agents* 2017; **50**(2): 285-6.
183. Prim N, Turbau M, Rivera A, Rodriguez-Navarro J, Coll P, Mirelis B. Prevalence of colistin resistance in clinical isolates of Enterobacteriaceae: A four-year cross-sectional study. *The Journal of infection* 2017; **75**(6): 493-8.
184. Ortiz de la Tabla V, Ortega A, Bunuel F, Perez-Vazquez M, Marcos B, Oteo J. Detection of the high-risk clone ST131 of Escherichia coli carrying the colistin resistance gene mcr-1 and causing acute peritonitis. *International journal of antimicrobial agents* 2017; **49**(1): 115-6.
185. Teo JW, Chew KL, Lin RT. Transmissible colistin resistance encoded by mcr-1 detected in clinical Enterobacteriaceae isolates in Singapore. *Emerging microbes & infections* 2016; **5**(8): e87.
186. Poirel L, Kieffer N, Brink A, Coetze J, Jayol A, Nordmann P. Genetic Features of MCR-1-Producing Colistin-Resistant Escherichia coli Isolates in South Africa. *Antimicrobial agents and chemotherapy* 2016; **60**(7): 4394-7.
187. Coetzee J, Corcoran C, Prentice E, et al. Emergence of plasmid-mediated colistin resistance (MCR-1) among Escherichia coli isolated from South African patients. *South African medical journal = Suid-Afrikaanse tydskrif vir geneeskunde* 2016; **106**(5): 35-6.
188. Newton-Foot M, Snyman Y, Maloba MRB, Whitelaw AC. Plasmid-mediated mcr-1 colistin resistance in Escherichia coli and Klebsiella spp. clinical isolates from the Western Cape region of South Africa. *Antimicrobial resistance and infection control*, 2017. (accessed 2017).
189. Yoon EJ, Hong JS, Yang JW, Lee KJ, Lee H, Jeong SH. Detection of mcr-1 Plasmids in Enterobacteriaceae Isolates From Human Specimens: Comparison With Those in Escherichia coli Isolates From Livestock in Korea. *Annals of laboratory medicine* 2018; **38**(6): 555-62.
190. Eiamphungporn W, Yainoy S, Jumderm C, Tan-Arsuwongkul R, Tiengrim S, Thamlikitkul V. Prevalence of colistin resistance gene mcr-1 in colistin-resistant Escherichia coli and Klebsiella pneumoniae isolated from humans in Thailand. *Journal of global antimicrobial resistance* 2018.
191. Paveenkittiporn W, Kerdsin A, Chokngam S, Bunthi C, Sangkitporn S, Gregory CJ. Emergence of plasmid-mediated colistin resistance and New Delhi metallo-beta-lactamase genes in extensively drug-resistant Escherichia coli isolated from a patient in Thailand. *Diagnostic microbiology and infectious disease* 2017; **87**(2): 157-9.
192. Le J, Turner N, Deshpande LM, Davis AP, Castanheira M. Case report of transient mcr-1-haboring Escherichia coli with concurrent Staphylococcus aureus bacteremia in Long Beach, California. *Diagnostic microbiology and infectious disease* 2017.
193. Kline KE, Shover J, Kallen AJ, Lonsway DR, Watkins S, Miller JR. Investigation of First Identified mcr-1 Gene in an Isolate from a U.S. Patient - Pennsylvania, 2016. *MMWR Morbidity and mortality weekly report* 2016; **65**(36): 977-8.

194. Mediavilla JR, Patrawalla A, Chen L, et al. Colistin- and Carbapenem-Resistant Escherichia coli Harboring mcr-1 and blaNDM-5, Causing a Complicated Urinary Tract Infection in a Patient from the United States. *mBio* 2016; **7**(4).
195. Tada T, Nhung PH, Shimada K, et al. Emergence of colistin-resistant Escherichia coli clinical isolates harboring mcr-1 in Vietnam. *International journal of infectious diseases : IJID : official publication of the International Society for Infectious Diseases* 2017; **63**: 72-3.
196. Torpdahl M, Hasman H, Litrup E, Skov RL, Nielsen EM, Hammerum AM. Detection of mcr-1-encoding plasmid-mediated colistin-resistant Salmonella isolates from human infection in Denmark. *International journal of antimicrobial agents* 2017; **49**(2): 261-2.
197. Aires CAM, da Conceicao-Neto OC, Tavares EOTR, Dias CF, Montezzi LF, Picao RC. Emergence of the Plasmid-Mediated mcr-1 Gene in Clinical KPC-2-Producing Klebsiella pneumoniae Sequence Type 392 in Brazil. 2017; **61**(7).
198. Srijan A, Margulieux KR, Ruekit S, et al. Genomic Characterization of Nonclonal mcr-1-Positive Multidrug-Resistant Klebsiella pneumoniae from Clinical Samples in Thailand. *Microbial drug resistance (Larchmont, NY)* 2018; **24**(4): 403-10.
199. Singh S, Pathak A, Kumar A, et al. Emergence of Chromosome-Borne Colistin Resistance Gene mcr-1 in Clinical Isolates of Klebsiella pneumoniae from India. *Antimicrobial agents and chemotherapy* 2018; **62**(2).
200. Mendes AC, Novais A, Campos J, et al. mcr-1 in Carbapenemase-Producing Klebsiella pneumoniae with Hospitalized Patients, Portugal, 2016-2017. *Emerg Infect Dis* 2018; **24**(4): 762-6.
201. Zhang Defu AH, Zhang Jian, Zhao Yuzong, Zhang Rui, Liu Xuefei, Yang Lina, Bai Fengling, Li Chun, Zhou Dongsheng, Li Yujin, Yin Zhe, Li Jianrong. Drug Resistance Analysis of a Multidrug-Resistant Proteus mirabilis Carrying the mcr-1 Gene Isolated from Food-Producing Animals. *Food Science* 2018; **39**(14): 145-50.
202. Teo JWP, Kalisvar M, Venkatachalam I, Ng OT, Lin RTP, Octavia S. mcr-3 and mcr-4 Variants in Carbapenemase-Producing Clinical Enterobacteriaceae Do Not Confer Phenotypic Polymyxin Resistance. *Journal of clinical microbiology* 2018; **56**(3).
